# Diallyl Trisulfide From Garlic Regulates RAB18 Phase Separation to Inhibit Lipophagy and Induce Cuproptosis in Hepatic Stellate Cells for Antifibrotic Effects

**DOI:** 10.1002/advs.202415325

**Published:** 2025-04-11

**Authors:** Haoyuan Tian, Shujiang Sun, Xinran Qiu, Junrui Wang, Yuanyuan Gao, Jianmei Chen, Xiang Han, Zhengyang Bao, Xiaohan Guo, Yuqi Sun, Yuxin Lin, Mengru Hu, Feng Zhang, Zili Zhang, Feixia Wang, Shizhong Zheng, Jiangjuan Shao

**Affiliations:** ^1^ Jiangsu Key Laboratory for Pharmacology and Safety Research of Chinese Materia Media Nanjing University of Chinese Medicine Nanjing 210023 China; ^2^ State Key Laboratory on Technologies for Chinese Medicine Pharmaceutical Process Control and Intelligent Manufacture Nanjing University of Chinese Medicine Nanjing 210023 China; ^3^ College of Electronic and Optical Engineering and College of Flexible Electronics (Future Technology) Nanjing University of Posts and Telecommunications Nanjing 210023 China

**Keywords:** diallyl trisulfide, hepatic stellate cells (HSCs), lipophagy, cuproptosis, phase separation, RAB18

## Abstract

Liver fibrosis, a common pathological process, severely impacts human health, yet effective treatments are lacking. Cuproptosis, a newly discovered form of cell death induced by copper ions, triggers cytotoxic stress through lipoylated protein oligomerization and may offer a novel therapeutic strategy for liver fibrosis. However, the mechanisms underlying cuproptosis in liver fibrosis are not well understood. During liver fibrosis progression, hepatic stellate cells (HSCs) activate, proliferate, and secrete extracellular matrix components, contributing to fibrosis. Activated HSCs also undergo lipophagy, the degradation of lipid droplets. The study shows that Ras‐related protein Rab‐18 (RAB18), a protein involved in lipid metabolism, inhibits lipophagy, upregulates Carnitine palmitoyltransferase 1A (CPT1A), and promotes succinylation of dihydrolipoamide dehydrogenase (DLD) at site K320, triggering cuproptosis in HSCs. Diallyl trisulfides (DATs), a garlic‐derived compound, induces phase separation of RAB18 and promotes mitochondrial‐associated membrane structures (MAMs) formation, further accelerating RAB18 phase separation. DATs selectively protects hepatocytes while activating cuproptosis in HSCs. Interfering with RAB18 expression reverses the DATs‐induced inhibition of lipophagy and cuproptosis. These findings, confirmed in primary cells, human liver stellate cells (LX2), rodent models and clinical samples, suggest that DATs, by targeting RAB18 and inducing its phase separation, subsequently inhibit lipophagy and promote cuproptosis, making it a promising therapeutic approach for liver fibrosis. [Correction added on 02 May 2025, after first online publication: In line 4 of the abstract, “sulfenylated” was updated to “lipoylated”.]

## Graphical abstract

Diallyl trisulfides (DATs) selectively induce cuproptosis in hepatic stellate cells (HSCs) by targeting Ras‐related protein Rab‐18 (RAB18) and regulating lipophagy. DATs promote RAB18 phase separation, enhance mitochondrial‐associated membrane structures (MAMs) formation, and increase succinylation of dihydrolipoamide dehydrogenase (DLD) at K320. This mechanism inhibits lipophagy, activates cuproptosis, offering a potential therapeutic target for liver fibrosis.

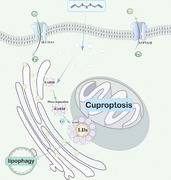

## 1 Introduction

Globally, liver fibrosis has a high prevalence among patients with chronic liver diseases, characterized by the abnormal proliferation of fibrous connective tissue within the liver.^[^ ^1^ ^]^ Copper is one of the essential trace elements in the human body, and when its concentration exceeds the normal physiological threshold, it can trigger a novel form of cell death known as “cuproptosis”.^[^ ^2^ ^]^ In this process, copper ions directly bind to acetylated components in the tricarboxylic acid cycle (TCA cycle), leading to their abnormal oligomerization. This, in turn, results in the loss of iron‐sulfur cluster proteins and protein toxicity stress, ultimately inducing cell death. Our research indicates that during the pathological progression of liver fibrosis, copper ion concentration becomes abnormally elevated, and the activation of hepatic stellate cells (HSCs) plays a critical role in this process. During liver injury, HSCs are activated and secrete large amounts of extracellular matrix components (such as collagen), thereby contributing to the formation of fibrosis. Due to the characteristics exhibited by HSCs in this process, they may be specifically induced to cell death via the copper ion‐mediated death mechanism. Therefore, regulating the copper levels within the liver may not only target cells at specific disease loci but also inhibit the progression of various chronic liver diseases.

A genome‐wide CRISPR‐Cas9 functional knockout screen identified ten key genes involved in cuproptosis, one of which is dihydrolipoamide dehydrogenase (DLD).^[^ ^2^ ^]^ DLD plays a central role in lipoic acid modification, catalyzing the redox reaction of lipoic acid to facilitate its cycling and the generation of active lipoic acid moieties, which are essential for protein lipoic acid modification.^[^ ^3^ ^]^ When intracellular copper ion levels become excessively high, copper ions interact with mitochondrial lipoic acid‐modified proteins, inducing structural changes and even aggregation, leading to the loss of iron‐sulfur cluster proteins and protein toxicity stress, ultimately resulting in cell death.^[^ ^2^ ^]^ Thus, DLD plays a crucial regulatory role in the copper‐induced cell death mechanism by maintaining the stability of the lipoic acid cycle.

HSCs are located in the Disse space of the liver, typically in a quiescent state, exhibiting characteristics of adipocytes and enriched with lipid droplets that store neutral lipids.^[^ ^4^ ^]^ During the progression of liver fibrosis, HSCs are activated and transform into myofibroblast‐like cells, accompanied by a significant reduction or disappearance of lipid droplets, a process known as “lipophagy”.^[^ ^5^ ^]^ Studies have shown that Ras‐related protein Rab‐18 (RAB18) plays a key role in both the formation and degradation of lipid droplets, with its expression level positively correlating with the number of lipid droplets in the cells. Therefore, abnormal regulation of RAB18 may influence the accumulation of lipid droplets, modulating the activation state of HSCs and thereby contributing to the progression of liver fibrosis. Fluctuations in lipid droplet numbers not only affect lipid metabolism but may also disrupt intracellular metal ion homeostasis, suggesting that lipid droplets may play an important role in metal ion‐dependent cell death, such as cuproptosis. Targeting the regulation of RAB18 to promote lipid droplet accumulation and inhibit HSCs via the cuproptosis pathway could offer a novel therapeutic strategy for liver fibrosis. Additionally, liquid–liquid phase separation (LLPS) of proteins is equally crucial in lipid droplet regulation, as it forms membrane‐less structures or microenvironmental compartments that modulate lipid droplet numbers to maintain cellular homeostasis. Membrane‐bound structures such as the endoplasmic reticulum and Golgi apparatus are key nodes in lipid droplet metabolism, and their dysfunction may lead to abnormal accumulation of copper ions within the cell, triggering cuproptosis. However, the specific mechanisms connecting lipophagy and cuproptosis remain unclear, and further research is needed to clarify their roles and effects in liver fibrosis.

Garlic, a plant with both medicinal and dietary uses, not only plays a prominent role in antioxidant, anti‐inflammatory, and hepatoprotective activities but also has no significant side effects, making it widely used due to its remarkable health benefits and safety.^[^ ^6^ ^]^ This makes garlic a high‐safety natural remedy that helps enhance immune function, reduce cardiovascular disease risk, and provides support for the prevention and treatment of chronic liver diseases. Modern pharmacological studies have shown that sulfur‐containing compounds in garlic can effectively alleviate oxidative stress in chronic liver diseases and protect hepatocytes from free radical damage.^[^ ^7^ ^]^ Allicin, the primary active component in garlic, collaborates with copper ions to produce a significant antimicrobial effect, a mechanism that involves promoting phospholipid peroxidation and enhancing selective copper ion accumulation in the cell membrane.^[^ ^8^ ^]^ However, due to the chemical instability of allicin, it decomposes into various active sulfur compounds, including diallyl disulfides (DADs) and diallyl trisulfides (DATs).^[^ ^9^ ^]^ DATs, one of the major active metabolites of allicin, exhibit multiple biological activities such as antioxidant, anti‐inflammatory, antimicrobial, and anticancer effects. In liver disease research, DATs have shown protective effects on the liver, alleviating oxidative stress and inflammation, and reducing the risk of liver fibrosis.^[^ ^10^ ^]^ It can also inhibit cell apoptosis and enhance antioxidant capacity, significantly mitigating alcohol‐induced hepatic steatosis and suppressing collagen deposition, thus alleviating liver damage and fibrosis induced by carbon tetrachloride (CCl₄).^[^ ^11^ ^]^ DATs also promote the generation of hydrogen sulfide (H₂S), inhibit HSCs activation induced by oxidative stress, and subsequently block the progression of fibrosis.^[^ ^12^ ^]^ Studies have shown that both DADs and DATs deplete intracellular glutathione (GSH) and release H₂S, with DATs depleting GSH and releasing H₂S at a faster rate compared to DADs.^[^ ^13^ ^]^ Since GSH is a metal ion chelator, its depletion leads to an increase in intracellular copper ion concentration, which in turn exacerbates cuproptosis.^[^ ^14^ ^]^ Therefore, DATs show extensive application prospects in the treatment of liver fibrosis, particularly in regulating hepatic stellate cell activity through the mechanism of cuproptosis.

In this study, we employed a range of technical approaches for a comprehensive analysis. First, we assessed the correlation between RAB18 expression and the progression of liver fibrosis by analyzing clinical liver pathology samples. Next, we conducted bioinformatics analysis using the Gene Expression Omnibus (GEO) clinical dataset and extracted primary cells for further examination. Additionally, we performed proteomics, transcriptomics, and protein mass spectrometry analyses to comprehensively explore the role of RAB18 in the disease mechanism. To further validate our findings, we employed atomic force microscopy to observe the behavior of RAB18 during phase separation. Through these multifaceted analyses, we thoroughly investigated the mechanism by which DATs target the RAB18 protein, inducing phase separation, inhibiting lipophagy, and enhancing the succinylation of dihydrolipoamide dehydrogenase (DLD) by recruiting Carnitine palmitoyltransferase 1A (CPT1A), thereby increasing DLD enzyme activity and ultimately inducing cuproptosis. Notably, we also discovered that after phase separation of RAB18, it promotes the coupling between the endoplasmic reticulum and mitochondria, driving the formation of mitochondrial‐associated membrane structures (MAMs), which in turn accelerates the phase separation of RAB18. This complex interaction ultimately results in the suppression of lipophagy and the initiation of cuproptosis. The findings of this study provide new insights into the relationship between lipophagy and cuproptosis, revealing their significant roles in liver fibrosis and offering a novel therapeutic strategy for the treatment of liver fibrosis.

### 1.1 Potential of DATs in Inducing Cuproptosis in Liver Fibrosis: Insights from GEO Clinical Data and Proteomics Analysis

Cuproptosis is a form of programmed cell death triggered by the excessive accumulation of copper ions in the body. However, the specific regulatory mechanisms of cuproptosis during the pathological progression of liver fibrosis remain to be further explored. Under normal physiological conditions, copper ions from food are absorbed in the small intestine, enter the liver via the portal vein, and bind to metal‐binding proteins such as metallothioneins to prevent the toxicity of free copper.^[^ ^15^ ^]^ The liver plays a central role in copper metabolism by binding copper to ceruloplasmin in plasma, facilitating its safe transport to peripheral tissues, while excess copper is excreted through bile to maintain copper homeostasis.^[^ ^16^ ^]^ Therefore, the liver plays a crucial role in regulating copper metabolism and excretion, making chronic liver diseases an excellent model for studying the mechanisms of cuproptosis.

**Figure 1:**
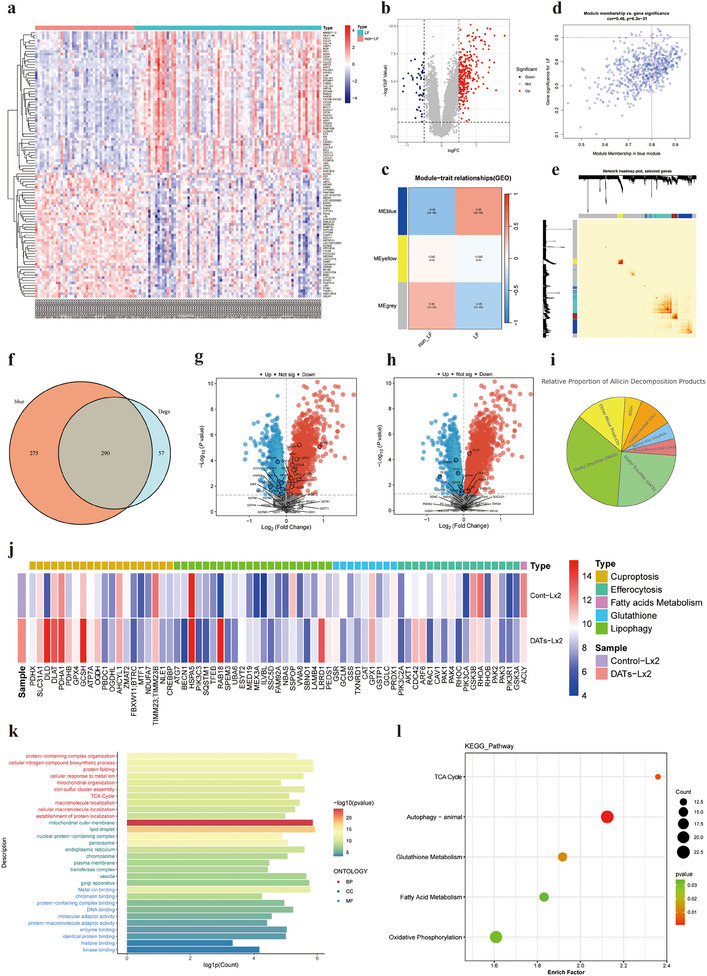
Potential of DATs in inducing cuproptosis in liver fibrosis: insights from GEO clinical data and proteomics analysis. a) A marked difference in gene expression profiles between fibrotic and normal tissues is observed, with the heatmap and volcano plot, b) illustrating the significance and expression trends of differentially expressed genes (DEGs). c) Weighted gene coexpression network analysis (WGCNA) identified a blue module significantly associated with liver fibrosis progression. d) Expression levels within the blue module showed a robust correlation with the severity of fibrosis, with statistical significance determined via the Kruskal–Wallis test. e) A network heatmap depicts the intricate interactions between genes within the blue module. f) The Venn diagram demonstrates substantial overlap between the blue module and DEGs, highlighting 290 shared genes potentially implicated in the progression of liver fibrosis. g) Gene set enrichment analysis (GSEA) identified key metabolic pathways associated with cuproptosis, particularly GSH metabolism and the tricarboxylic acid (TCA) cycle, with h) critical changes in the expression of genes involved in these pathways. i) Allicin, an unstable bioactive compound, rapidly decomposes under environmental conditions, predominantly forming DADS and DATs. j) Proteomic profiling of human hepatic stellate cells (LX2) treated with DATs revealed significant alterations in proteins related to copper metabolism, suggesting that DATs may induce cuproptosis in HSCs. Notable changes were observed in genes and proteins linked to copper metabolism, GSH metabolism, fatty acid metabolism, and lipophagy pathways. k) Gene Ontology (GO) analysis demonstrated significant enrichment of differentially expressed genes in protein metabolic processes and metal ion binding, in line with the central mechanisms of cuproptosis. l) KEGG pathway analysis further revealed significant enrichment of autophagy‐related pathways, underscoring the role of autophagy in maintaining intracellular copper homeostasis and regulating copper metabolic balance.

To investigate the potential role of cuproptosis in liver fibrosis, we conducted a comprehensive bioinformatics analysis of the GSE84044 clinical dataset from the GEO database. First, differential expression gene (DEG) analysis was performed using DESeq2 or edgeR to identify genes that were significantly differentially expressed at various stages of fibrosis. We applied *P*‐value and false discovery rate (FDR) corrections to ensure statistical significance. Volcano plots and heatmaps were used to display the significance and expression patterns of these genes, revealing gene expression differences between different samples (**Figure** **1**a,b). Subsequently, we constructed a gene coexpression network using weighted gene coexpression network analysis (WGCNA) to identify gene modules that were highly correlated with the progression of liver fibrosis. The blue module, in particular, was significantly enriched with key genes closely related to fibrosis progression (Figure 1c). Further analysis indicated that the expression levels of these genes in the blue module were significantly correlated with the severity of fibrosis (Figure 1d). A network heatmap revealed the interaction relationships of genes within the blue module (Figure 1e). To further validate the overlap between the differentially expressed genes (DEGs) and the key gene modules identified by WGCNA, we performed a Venn diagram analysis. The results showed a significant overlap between the blue module and the DEGs, containing a total of 290 genes that may play a crucial role in the progression of liver fibrosis (Figure 1f). To explore the functions of these differential genes and the biological pathways they are involved in, we conducted gene set enrichment analysis (GSEA), focusing on metabolic pathways related to copper death, especially changes in key genes in the GSH metabolism and TCA cycle (Figure 1g,h). The analysis indicated that core genes of the GSH metabolism pathway were significantly dysregulated in liver fibrosis patients, particularly those involved in the TCA cycle and closely related to copper death, such as DLD, Succinate Dehydrogenase Complex Subunit B (SDHB), Dihydrolipoamide Acetyltransferase (DLAT) and Dihydrolipoamide Succinyltransferase (DLST), all of which showed significant disruption. These aberrant expressions may be closely linked to the elevated copper levels in the livers of fibrosis patients, suggesting that the copper death mechanism could play a critical role in the pathological progression of liver fibrosis.

Allicin is an extremely unstable active compound primarily composed of DADs and DATs.^[^ ^17^ ^]^ It rapidly decomposes under exposure to air, light, or higher temperatures, generating a series of sulfur‐containing compounds. Among these, DADS and DATs are the two major products formed during the decomposition of allicin,^[^ ^9^ ^]^ accounting for the highest proportions of the decomposition products (Figure 1i). Studies have shown that both DADS and DATs effectively deplete intracellular GSH, with DATs demonstrating a faster rate of GSH consumption and hydrogen sulfide (H₂S) release compared to DADs.^[^ ^13^ ^]^ This characteristic endows DATs with strong therapeutic potential in liver disease research. Since GSH acts as a metal ion chelator and reduces the concentration of free metal ions in the cell, its depletion may render the cell more susceptible to cuproptosis, a form of copper‐dependent cell death.

We further treated the human hepatic stellate cell line LX2 with DATs and conducted a proteomic analysis, focusing on the expression of copper death‐related proteins. The experimental results showed that DATs treatment significantly altered the gene and protein expression of multiple metabolic pathways in LX2 cells, particularly in pathways related to copper death, GSH metabolism, fatty acid metabolism, and lipophagy. Specifically, copper death‐related genes (such as DLD, PDHX, and DLAT) and their corresponding proteins were significantly upregulated in the DATs‐treated group (DATs‐LX2), suggesting that DATs might exert its effect in LX2 cells by activating the copper death pathway. Moreover, significant changes in genes and proteins related to GSH metabolism, such as Glutathione‐Disulfide Reductase (GSR) and Glutamate‐Cysteine Ligase Modifier Subunit (GCLM), suggest that this metabolic pathway may be involved in the antifibrotic effects of DATs. Additionally, the differential expression of genes and proteins associated with fatty acid metabolism and lipophagy indicates that DATs might influence the function of HSCs through the regulation of lipid metabolism pathways (Figure 1j). To further explore the molecular mechanisms underlying the antifibrotic action of DATs, we performed Gene Ontology (GO) and Kyoto Encyclopedia of Genes and Genomes (KEGG) analyses on the proteomic data. GO analysis revealed that the differentially expressed genes were significantly enriched in processes such as protein metabolism and metal ion binding, which are closely related to the core mechanisms of copper death that depend on the accumulation of intracellular copper ions and abnormal protein modification (Figure 1k). KEGG pathway analysis further indicated that the differentially expressed genes were significantly enriched in the autophagy pathway, suggesting that the autophagy pathway may play a key role in maintaining intracellular copper ion homeostasis and regulating copper metabolic balance (Figure 1l).

To further investigate the enrichment of related pathways and signaling proteins, we conducted an in‐depth GSEA analysis of the proteomic data. The results confirmed that the expression of proteins related to autophagy and copper metabolism pathways exhibited significant changes (Figure S10f,g, Supporting Information). Through the integration of GO and KEGG analyses, we hypothesize that DATs may regulate these key metabolic and cell death pathways to induce cuproptosis in HSCs, thus playing a role in the progression of liver fibrosis. This provides important theoretical support for further understanding the potential mechanisms of DATs in the treatment of fibrosis.

### 1.2 DATs Suppress HSCs Growth via Copper‐Induced Death Pathway

**Figure 2:**
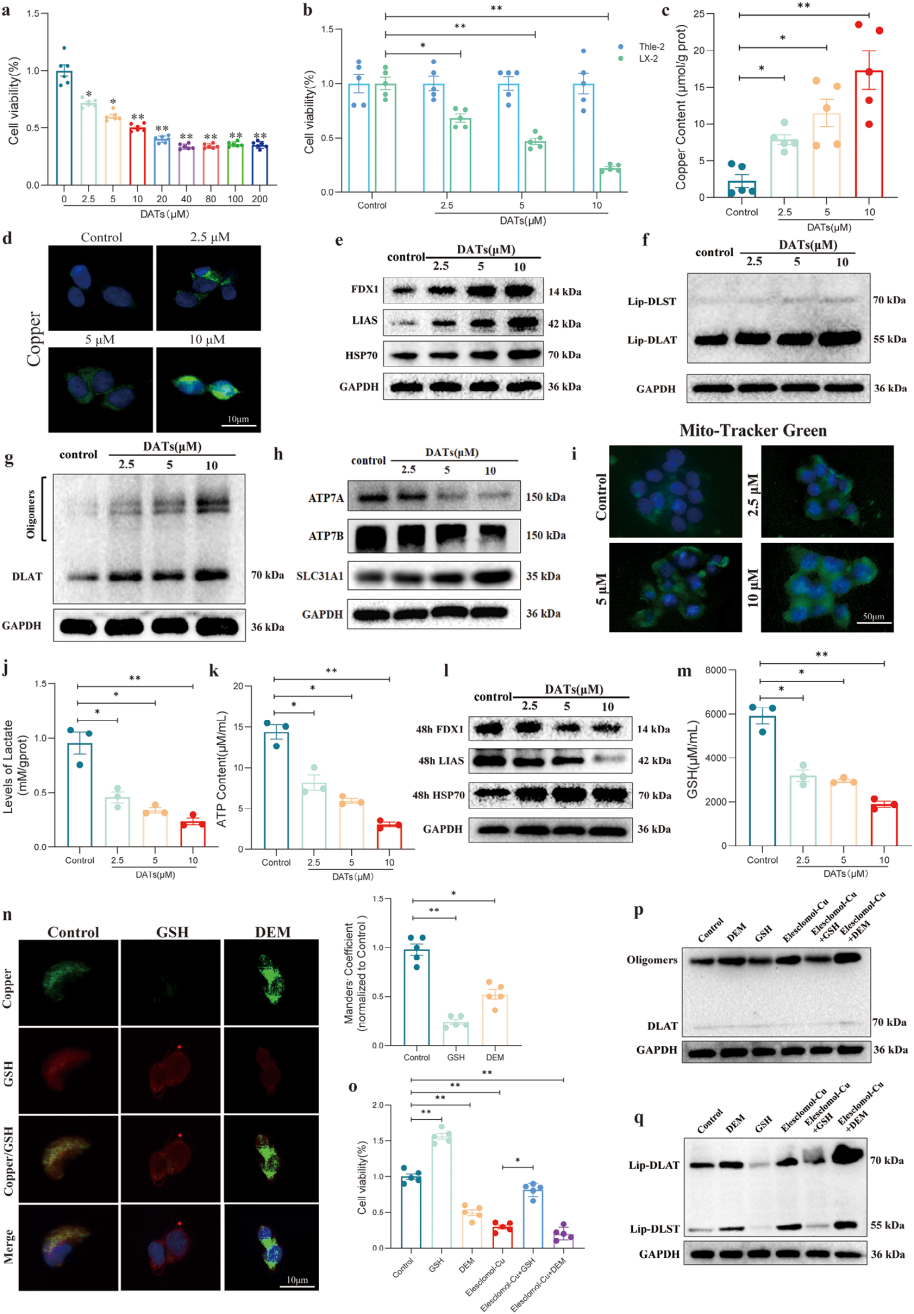
DATs suppress HSCs growth via copper‐induced death pathway. a) LX2 cells were treated with DATs (0–200 µm) for 24 h to determine the optimal concentration for DATs intervention (*n* = 5). b) LX2 and Thle2 cells were treated with DATs (0–10 µm) for 24 h. DATs significantly inhibited the viability of LX2 cells, while no significant effect was observed on the normal liver cells, Thle2 (*n* = 5). c) LX2 cells were treated with DATs (0–10 µm) for 24 h and copper ion content within the cells was assessed using a copper ion detection kit (*n* = 3). d) LX2 cells were treated with DATs (0–10 µm) for 24 h. Fluorescence images of the cells stained with *N*‐aminofluorescein, a fluorescent probe highly selective and sensitive to Cu^2+^, were captured (*n* = 3). Scale bar: 50 µm. e) Immunoblotting was performed to evaluate the expression levels of FDX1, LIAS, and HSP70 proteins in LX2 cells treated with DATs (0–10 µm) for 24 h and the protein expression was quantified through grayscale analysis (*n* = 3). f) Immunoblotting was performed to assess the expression levels of Lip‐DLST and Lip‐DLAT proteins in LX2 cells treated with DATs (0–10 µm) for 24 h, followed by quantification using grayscale analysis (*n* = 3). g) Immunoblotting was conducted to examine the oligomerization of DLAT protein in LX2 cells treated with DATs (0–10 µm) for 24 h and the results were quantified by grayscale analysis (*n* = 3). h) Immunoblotting was used to analyze the expression levels of SLC31A1, ATP7A, and ATP7B in LX2 cells treated with DATs (0–10 µm) for 24 h, with quantification by grayscale analysis (*n* = 3). i) LX2 cells were treated with DATs (0–10 µm) for 24 h. Fluorescence images of cells stained with Mito‐Tracker Green were obtained (*n* = 3). Scale bar: 50 µm. j) LX2 cells were treated with DATs (0–10 µm) for 24 h, and lactate levels and ATP. k) Content were measured using lactate or ATP detection kits (*n* = 3). l) Immunoblotting was performed to assess the expression levels of FDX1, LIAS, and HSP70 proteins in LX2 cells treated with DATs (0–10 µm) for 48 h and protein expression was quantified through grayscale analysis (*n* = 3). m) LX2 cells were treated with DATs (0–10 µm) for 24 h and GSH content in the cells was measured using a GSH detection kit (*n* = 3). n) Immunofluorescence imaging and analysis were performed on LX2 cells treated with DEM (200 µm) or GSH (1 mm) for 24 h, to observe the colocalization of Copper (green) and GSH (red) (*n* = 5). Scale bar: 10 µm. o) CCK8 assay was performed to evaluate the viability of LX2 cells treated with DEM (200 µm) or GSH (1 mm) for 24 h, in the presence or absence of Elesclomol‐Cu (10 nm) (*n* = 5). p) Immunoblotting was performed to assess the expression levels of Lip‐DLST and Lip‐DLAT in LX2 cells treated with DEM (200 µm) or GSH (1 mm) for 24 h, in the presence or absence of Elesclomol‐Cu (10 nm) (*n* = 3). q) Immunoblotting was used to examine the expression levels of oligomerized DLAT protein in LX2 cells treated with DEM (200 µm) or GSH (1 mm) for 24 h, in the presence or absence of Elesclomol‐Cu (10 nm) (*n* = 3). Data are presented as mean ± Standard Deviation (SD), with *p*‐values calculated using one‐way ANOVA. ns, not significant; *p < 0.05, **p < 0.01. [Correction added on 02 May 2025, after first online publication: In the Figure 2n caption, “*n* = 3” was updated to “*n* = 5”.]

To further explore the molecular mechanism by which DATs induce cuproptosis in HSCs to inhibit their proliferation, we first performed a cell proliferation assay using the Cell Counting Kit‐8 (CCK8) to screen for the optimal concentration of DATs in LX2 cells. The appropriate concentration for DATs intervention was determined (**Figure** **2**a). Subsequently, we applied this concentration to treat normal liver cells (Thle‐2) and conducted control experiments. The results showed that DATs significantly inhibited the viability of LX2 cells at this concentration, while no significant effect on the viability of normal liver cells was observed (Figure 2b). To further investigate the selective effects of DATs on LX2 cells, we measured the level of cuproptosis in both Thle‐2 and LX2 cells after DATs intervention, using 10 nm Elesclomol‐Cu as a positive control. The experimental results showed that DATs (at a concentration of 10 µm) effectively induced cuproptosis in LX2 cells, while no significant cuproptosis was observed in normal liver cells (Thle‐2) (Figure S2a,b, Supporting Information). The results indicate that DATs exhibit a highly selective targeting effect on HSCs, which may be related to the unique properties of garlic, a food and medicinal plant from which DATs are derived. The selective effects of DATs arise from differences in metabolic patterns, copper homeostasis, antioxidant capacity, and signaling pathways between HSCs and normal liver cells. This is likely because HSCs exhibit higher mitochondrial activity, an imbalance in copper metabolism, and weaker antioxidant defenses, making them more susceptible to cuproptosis induced by DATs. In contrast, normal liver cells are able to effectively resist the toxic effects of DATs through flexible metabolic regulation, efficient copper efflux mechanisms, and strong antioxidant defenses, thus allowing them to survive. To further elucidate the mechanism of cuproptosis triggered by DATs, we measured the intracellular copper ion content in LX2 cells under gradient dosing conditions. The experimental results showed that DATs significantly increased the intracellular copper ion content (Figure 2c). Additionally, fluorescence probe detection for copper ions further confirmed the increase in copper ion concentration in LX2 cells (Figure 2d). Furthermore, biomarker analysis for cuproptosis revealed that with increasing doses of DATs, the expression levels of key proteins such as Ferredoxin 1 (FDX1), Lipoic Acid Synthetase (LIAS), and Heat Shock Protein 70 (HSP70) were significantly upregulated (Figure 2e and Figure S2c, Supporting Information). Similarly, the expression of proteins associated with lipoic acid modification, a hallmark of cuproptosis, was also significantly increased (Figure 2f and Figure S2d, Supporting Information). Nondenaturing gel electrophoresis results showed that the excessive intracellular copper ions induced by DATs significantly increased the oligomerization of the DLAT protein (Figure 2g and Figure S2e, Supporting Information). Additionally, the expression of the copper ion influx protein SLC31A1 was significantly upregulated, while the expression of the copper ion efflux proteins ATP7A and ATP7B was significantly downregulated (Figure 2h and Figure S2f, Supporting Information). These findings suggest that DATs induce cuproptosis in LX2 cells by regulating copper ion homeostasis and the function of related proteins.

Previous studies have shown that cuproptosis tends to occur in cells that rely on mitochondrial respiration. Therefore, this study specifically evaluated changes in mitochondrial quantity, ATP content, and lactate levels after DATs treatment. The experimental results indicated that DATs treatment significantly increased the number of mitochondria (Figure 2i). At the same time, glycolysis was inhibited, and both lactate levels and Adenosine Triphosphate (ATP) content were significantly downregulated (Figure 2j,k). This metabolic shift may create a favorable environment for the occurrence of cuproptosis and further reveal the underlying mechanisms behind the highly selective targeting of HSCs by DATs. To further clarify the potential time‐dependent nature of cuproptosis, we compared the expression of related proteins at 24 and 48 h after DATs treatment. The results showed that the expression of FDX1 and LIAS increased at 24 h but significantly decreased at 48 h (Figure 2l and Figure S2g, Supporting Information). This suggests a time‐dependent regulation of cuproptosis‐related proteins during the process, providing further insight into the dynamics of the copper‐induced cell death mechanism.

In GSH‐depleted cells, the concentration of metal ions typically increases, which may promote cell death induced by metal ions. To further elucidate the mechanism of cuproptosis triggered by DATs, we measured the GSH content in LX2 cells under gradient dosing conditions. The experimental results showed that DATs significantly reduced the intracellular GSH content (Figure 2m). Laser confocal microscopy for copper ion and GSH fluorescence colocalization revealed a significant negative correlation between copper ion concentration and GSH levels (Figure 2n).

To further investigate the relationship between cell viability, GSH, and cuproptosis, we performed a cell proliferation assay. The results showed that an increase in GSH concentration significantly enhanced cell viability, whereas GSH depletion (using DEM treatment) led to a significant decrease in cell viability. During the decrease in cell viability induced by Elesclomol‐Cu, the addition of GSH significantly reversed the effect; however, when Elesclomol‐Cu was combined with Diethyl Maleate (DEM) treatment, cell viability decreased significantly more than when Elesclomol‐Cu was used alone (Figure 2o). To explore the role of GSH in cellular cuproptosis, we used Elesclomol‐Cu (10 nm), exogenous GSH (1 mm), or the GSH depleting agent DEM (200 µm), and analyzed the relationship between GSH and cuproptosis by detecting the expression of lipoic acid‐modified proteins and the oligomerization of DLAT. The experimental results showed that the addition of exogenous GSH significantly inhibited cuproptosis, while the use of the GSH depleting agent DEM significantly enhanced the cuproptosis levels. Specifically, the cuproptosis effect induced by Elesclomol‐Cu was significantly reversed by the addition of GSH, whereas the cuproptosis level increased significantly after cotreatment with Elesclomol‐Cu and DEM compared to using Elesclomol‐Cu alone (Figure 2p,q and Figure S2h,i, Supporting Information). These results further highlight the crucial role of GSH in regulating cellular cuproptosis.

In summary, DATs demonstrated selective inhibitory potential against HSCs under pathological conditions, revealing its mechanism of inhibiting HSCs growth through the induction of cuproptosis.

### 1.3 DATs Promote DLD Accumulation and Induce Cuproptosis by Inhibiting Its Ubiquitination

**Figure 3:**
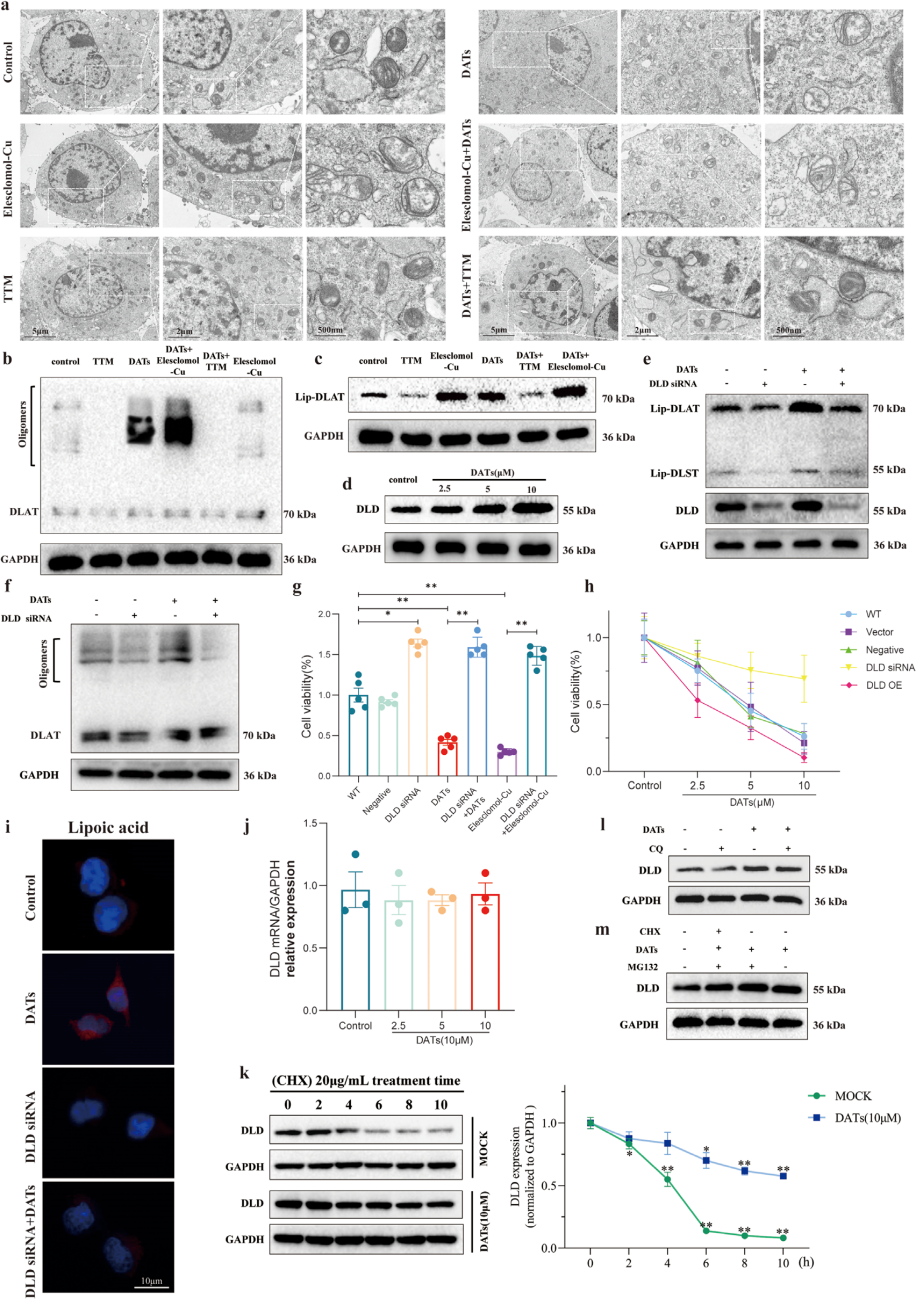
DATs promote DLD accumulation and induce cuproptosis by inhibiting its ubiquitination. a) LX2 cells were treated with Elesclomol‐Cu (10 nm) or TTM (5 µm) for 24 h in the presence or absence of DATs (10 µm). Transmission electron microscopy images were captured. The right image is an enlarged view of the boxed region from the left image. Scale bar: 5 µm. b) Immunoblotting was performed to evaluate the expression levels of oligomerized DLAT protein in LX2 cells treated with Elesclomol‐Cu (10 nm) or TTM (5 µm) for 24 h, in the presence or absence of DATs (10 µm), and the results were quantified using grayscale analysis (*n* = 3). c) Immunoblotting was performed to assess the expression levels of lipoic acid‐modified DLAT protein in LX2 cells treated with Elesclomol‐Cu (10 nm) or TTM (5 µm) for 24 h, in the presence or absence of DATs (10 µm), and the results were quantified using grayscale analysis (*n* = 3). d) Immunoblotting was performed to evaluate the expression levels of DLD protein in LX2 cells treated with DATs (0–10 µm) for 24 h, with quantification using grayscale analysis (*n* = 3). e) Immunoblotting was performed to assess the expression levels of Lip‐DLST and Lip‐DLAT in LX2 cells treated with DATs (10 µm) and transfected with DLD siRNA or negative control siRNA, followed by quantification through grayscale analysis (*n* = 3). f) Immunoblotting was used to evaluate the expression levels of oligomerized DLAT protein in LX2 cells treated with DATs (10 µm) and transfected with DLD siRNA or negative control siRNA, with quantification by grayscale analysis (*n* = 3). g) CCK8 assay was performed to assess the viability of LX2 cells treated with DATs (10 µm) or Elesclomol‐Cu (10 nm) and transfected with DLD siRNA or negative control siRNA (*n* = 5). h) LX2 cells were treated with DATs (0–10 µm) for 24 h. CCK8 assay was performed to evaluate the viability of cells transfected with DLD siRNA, negative control small interfering RNA (siRNA)/Vector, or DLD Overexpression (OE) (*n* = 5). i) Immunofluorescence images of Lip‐DLST and Lip‐DLAT were captured in LX2 cells treated with DATs (10 µm) and transfected with DLD siRNA or negative control siRNA (*n* = 3). Scale bar: 10 µm. j) Reverse Transcription Quantitative Polymerase Chain Reaction (RT‐qPCR) was performed to evaluate the expression levels of DLD mRNA in LX2 cells treated with DATs (0–10 µm) for 24 h (*n* = 3). k) Immunoblotting was performed to evaluate the DLD protein half‐life in LX2 cells treated with DATs (10 µm) for 24 h, with or without protein synthesis inhibitors, and the results were quantified by grayscale analysis (*n* = 3). l) Immunoblotting was performed to assess the DLD protein expression levels in LX2 cells treated with DATs (10 µm) for 24 h, in the presence or absence of chloroquine (CQ, 5 µm), and the results were quantified by grayscale analysis (*n* = 3). m) Immunoblotting was used to evaluate the DLD protein expression levels in LX2 cells treated with DATs (10 µm) for 24 h, and treated with cycloheximide (CHX, 20 µg mL^−1^), MG132 (10 µm) alone or in combination, with quantification by grayscale analysis (*n* = 3). Data are presented as mean ± SD, with *p*‐values calculated using one‐way analysis of variance (ANOVA). ns, not significant; *p < 0.05, **p < 0.01.

Currently, the ultrastructural characteristics of copper death cells are not well‐defined. Therefore, this study used copper ion carrier drug Elesclomol‐Cu, copper death inhibitor Tetrathiomolybdate (TTM), and DATs, either alone or in combination, to treat cells and explored the morphological features of copper death through transmission electron microscopy (TEM). On one hand, this aimed to verify the potential ultrastructural characteristics of copper death cells, and on the other hand, further clarify whether DATs induce copper death. The experimental results revealed the following characteristics, which are distinct from other known forms of cell death: 1) Mitochondrial abnormalities: Mitochondria were significantly swollen, with separation of the inner and outer membranes (uncoupling). The cristae structure was completely disintegrated, and a large accumulation of dense particles was observed. Additionally, there were abnormal folds of the inner membrane, membrane disruption, and a significant decrease in outer membrane density. 2) Protein aggregation and copper destabilization: Protein aggregates related to copper ion destabilization were detected in both the cytoplasm and mitochondria. 3) Endoplasmic reticulum changes: The endoplasmic reticulum was significantly expanded, with copper‐related particle accumulation observed within the membrane system. 4) Plasma membrane damage: Localized ruptures in the plasma membrane were observed, exhibiting region‐specific damage rather than random destruction. 5) Nuclear structural changes: Chromatin showed irregular aggregation within the cell nucleus, accompanied by nuclear membrane rupture and the diffusion of chromatin into the cytoplasm. The nuclear volume underwent abnormal changes, with protrusions and indentations observed on the nuclear surface (**Figure** **3**a). We further investigated the oligomerization of the key copper death biomarker DLAT protein and the expression levels of lipoic acid‐modified proteins (Figure 3b,c and Figure S3a,b, Supporting Information). The results definitively confirmed that under these conditions, copper death occurred in the cells and provided strong support for the morphological characteristics observed under transmission electron microscopy. In summary, these structural changes are distinct from other forms of cell death, particularly the separation of mitochondrial membranes, protein aggregation, copper particle accumulation in the endoplasmic reticulum, and localized plasma membrane damage. These features are closely related to copper‐induced metabolic disruption and may serve as specific ultrastructural markers of cuproptosis, providing new clues for further exploration of the molecular mechanisms of copper death.

Based on the results of the proteomic analysis, the study found that after DATs treatment, the expression of genes related to lipoic acid synthesis was significantly upregulated, with the expression of DLD protein showing a particularly notable increase. To further elucidate the regulatory role of DLD, we investigated the expression changes of DLD under gradient concentrations. The experimental results indicated that as the DATs concentration increased, the expression level of DLD protein showed a significant upward trend (Figure 3d and Figure S3c, Supporting Information). To further validate the role of DLD in the process of cellular cuproptosis, we used small interfering RNA (siRNA) to downregulate DLD expression and analyzed the levels of lipoic acid‐modified proteins and DLAT protein oligomerization. The results showed that when DLD expression was interfered with, the expression of lipoic acid‐modified proteins and DLAT was significantly reduced. DATs treatment significantly promoted the oligomerization of lipoic acid‐modified proteins and DLAT in the cells, but this increase in expression was significantly reversed after interference with DLD expression (Figure 3e,f and Figure S3d,e, Supporting Information). Using the CCK8 assay to measure cell viability, we further demonstrated that silencing DLD expression could significantly reverse the decrease in cell viability induced by DATs or Elesclomol‐Cu (Figure 3g,h). To further confirm the regulatory role of DLD in copper death, we performed similar experiments in 293T cells, yielding consistent results. The experimental data showed that after treatment with different concentrations of Elesclomol‐Cu, the survival rate of cells overexpressing DLD was significantly lower than that of the wild‐type (WT) and Negative groups, while interference with DLD expression effectively reversed the decrease in cell viability induced by Elesclomol‐Cu (Figure S1h, Supporting Information). Additionally, we further demonstrated that interference with DLD expression significantly reversed the decrease in cell viability caused by Elesclomol‐Cu (Figure S1I, Supporting Information). These results suggest that DLD plays a key regulatory role in the copper death process regulated by DATs. Immunofluorescence analysis further showed that after interference with DLD expression, the level of lipoic acid significantly decreased. DATs treatment significantly increased the intracellular lipoic acid content, and this increase was significantly reversed when DLD expression was interfered with (Figure 3i).

Further investigation revealed that the upregulation of DLD protein might be related to the inhibition of its degradation rather than changes in gene expression levels.^[^ ^18^ ^]^ Gene expression analysis confirmed that after DATs treatment, there was no significant change in DLD mRNA levels (Figure 3j). Next, we evaluated the impact of DATs on the stability of DLD protein. After treatment with the protein synthesis inhibitor cycloheximide (CHX), we found that DATs significantly extended the half‐life of DLD, suggesting that DATs may exert its effect by inhibiting the degradation of DLD (Figure 3k). As is well known, proteins are degraded primarily through two pathways—the ubiquitin‐proteasome system and the autophagy‐lysosome pathway. We used the autophagy inhibitor chloroquine (CQ) in the experiment, and the results showed that CQ had no significant effect on the degradation of DLD (Figure 3l and Figure S3f, Supporting Information). We then used CHX and the proteasome inhibitor MG132 to investigate whether DATs increase DLD accumulation by inhibiting its ubiquitination. As shown in the figure, MG132 enhanced DATs‐mediated DLD expression. However, when CHX was combined with DATs and MG132, the DATs‐mediated upregulation of DLD was significantly weakened (Figure 3m and Figure S3g, Supporting Information). These results suggest that the degradation of DLD primarily occurs through the proteasomal pathway. In summary, DATs regulate the copper death process by stabilizing DLD expression through the inhibition of its ubiquitin‐mediated degradation.

### 1.4 DATs Promote Cuproptosis by Modulating CPT1A to Regulate Site Competition between Succinylation and Ubiquitination of DLD

The dysregulation of the TCA cycle is a hallmark feature of cuproptosis.^[^ ^2^ ^]^ In this process, the intracellular level of succinyl‐CoA significantly increases.^[^ ^19^ ^]^ Succinyl‐CoA is not only an important intermediate in energy metabolism but also plays a role in protein succinylation by providing succinyl groups, thus regulating various pathological processes.^[^ ^20^ ^]^ During the onset and progression of chronic liver diseases, protein succinylation plays a crucial regulatory role in the disease process, with mechanisms including the regulation of key enzyme activities and the mediation of intracellular signaling.^[^ ^21^ ^]^

DLD is a core component of several enzyme complexes, including the pyruvate dehydrogenase complex (PDH), the α‐ketoglutarate dehydrogenase complex (OGDH), and the branched‐chain amino acid dehydrogenase complex.^[^ ^3^ ^]^ In metabolic pathways, the OGDH complex, in which DLD is involved, is responsible for catalyzing the conversion of α‐ketoglutarate to succinyl‐CoA. Succinyl‐CoA not only acts as a key metabolic intermediate but also serves as a donor for succinylation modifications, regulating the function and stability of proteins.^[^ ^3^ ^]^ Based on this, we hypothesize that during DLD expression, DATs may play an important role in the metabolic regulation of cuproptosis and chronic liver diseases by affecting the level of succinylation modification of DLD itself.

**Figure 4:**
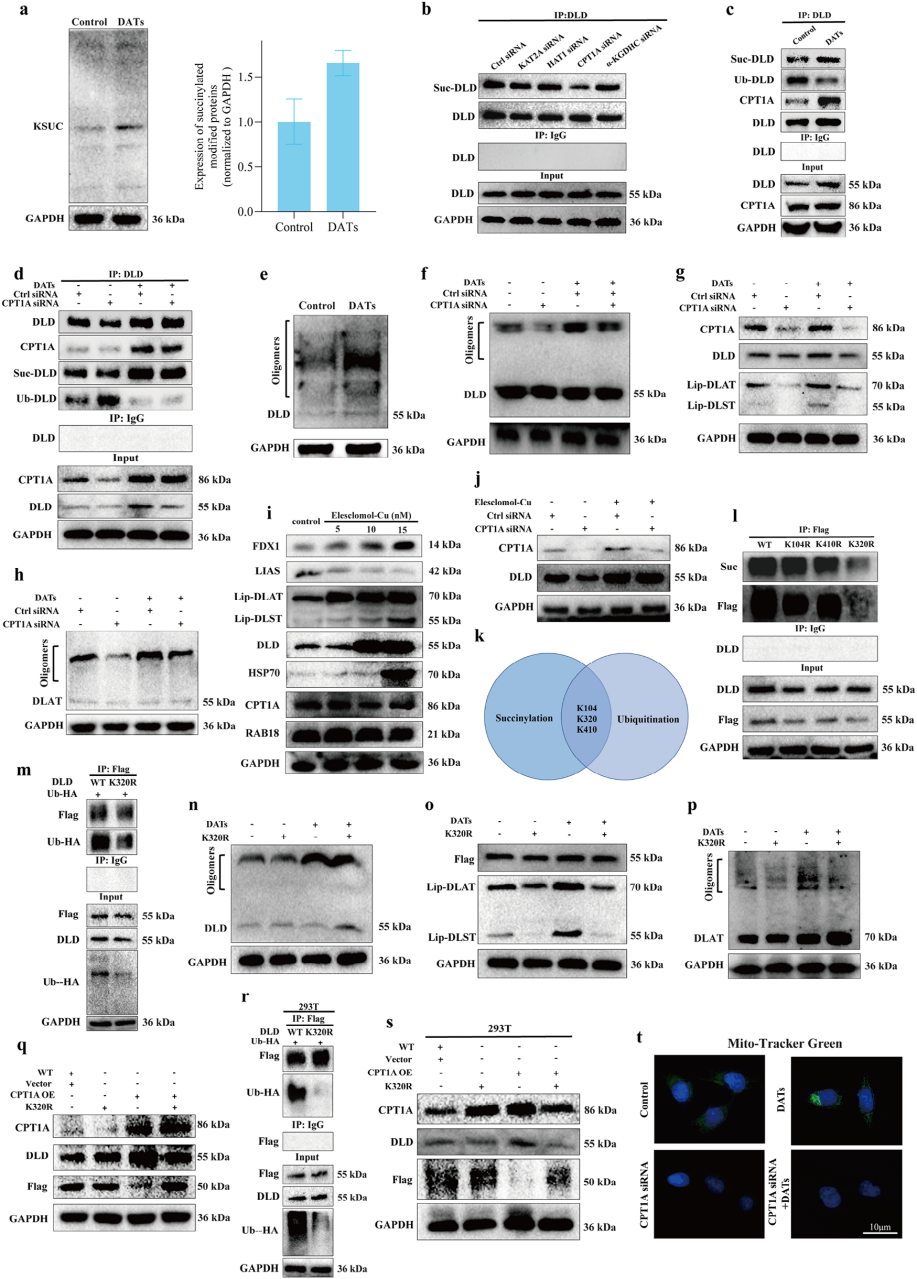
DATs promote cuproptosis by modulating CPT1A to regulate site competition between succinylation and ubiquitination of DLD. a) Immunoblotting was performed to evaluate the expression levels of succinylated proteins in LX2 cells treated with DATs (10 µm) for 24 h, and the results were quantified using grayscale analysis (*n* = 3). b) IP assays were performed to assess the level of DLD protein succinylation in LX2 cells following interference with the expression of four known succinylation‐modifying enzymes (*n* = 3). c) Co‐IP assays were conducted to evaluate the interaction between CPT1A and DLD in LX2 cells treated with DATs (10 µm) for 24 h, and to assess the succinylation and ubiquitination levels of DLD protein (*n* = 3). d) Co‐IP assays were performed to evaluate the interaction between CPT1A and DLD in LX2 cells transfected with CPT1A siRNA or negative control siRNA, treated with DATs (10 µm), and to assess the succinylation and ubiquitination levels of DLD protein (*n* = 3). e) Immunoblotting was performed to assess the expression levels of oligomerized DLD protein in LX2 cells treated with DATs (10 µm) for 24 h and the results were quantified using grayscale analysis (*n* = 3). f) Immunoblotting was used to evaluate the expression levels of oligomerized DLD protein in LX2 cells transfected with CPT1A siRNA or negative control siRNA and treated with DATs (10 µm), followed by grayscale analysis for quantification (*n* = 3). g) Immunoblotting was performed to assess the expression levels of DLD, Lip‐DLST, and Lip‐DLAT in LX2 cells transfected with CPT1A siRNA or negative control siRNA and treated with DATs (10 µm), with quantification through grayscale analysis (*n* = 3). h) Immunoblotting was used to evaluate the expression levels of oligomerized DLAT protein in LX2 cells transfected with CPT1A siRNA or negative control siRNA and treated with DATs (10 µm), followed by grayscale analysis for quantification (*n* = 3). i) Immunoblotting was performed to assess the expression levels of CPT1A, DLD, FDX1, LIAS, HSP70 and other proteins in LX2 cells treated with Elesclomol‐Cu (0–10 nm) for 24 h, with quantification using grayscale analysis (*n* = 3). j) Immunoblotting was performed to evaluate the expression levels of DLD protein in LX2 cells transfected with CPT1A siRNA or negative control siRNA and treated with Elesclomol‐Cu (10 nm), with grayscale analysis for quantification (*n* = 3). k) The GPSuc tool was used to predict multiple succinylation sites on DLD protein, and an intersection analysis with the ubiquitination sites from the iPTMnet and PhosphoSitePlus databases identified K104, K320, and K410 as overlapping sites. l) IP assays were conducted to assess the succinylation modification levels of DLD protein in LX2 cells transfected with FLAG‐tagged DLD‐WT, DLD‐104R, DLD‐320R, or DLD‐410R plasmids (*n* = 3). m) IP assays were used to evaluate the ubiquitination levels of DLD protein in LX2 cells transfected with FLAG‐tagged DLD‐320R and Ub‐HA plasmids (*n* = 3). n) Immunoblotting was performed to assess the expression levels of oligomerized DLD protein in LX2 cells treated with DATs (10 µm) and transfected with FLAG‐tagged DLD‐320R, followed by grayscale analysis for quantification (*n* = 3). o) Immunoblotting was conducted to evaluate the expression levels of Lip‐DLST and Lip‐DLAT in LX2 cells treated with DATs (10 µm) and transfected with FLAG‐tagged DLD‐320R, with grayscale analysis for quantification (*n* = 3). p) Immunoblotting was used to assess the expression levels of oligomerized DLAT protein in LX2 cells treated with DATs (10 µm) and transfected with FLAG‐tagged DLD‐320R, followed by grayscale analysis for quantification (*n* = 3). q) Immunoblotting was performed to assess the expression levels of oligomerized CPT1A and DLD proteins in LX2 cells treated with CPT1A OE and transfected with DLD‐320R, with grayscale analysis for quantification (*n* = 3). r) IP assays were used to assess the ubiquitination levels of DLD protein in 293T cells transfected with FLAG‐tagged DLD‐320R and Ub‐HA plasmids (*n* = 3). s) Immunoblotting was performed to evaluate the expression levels of oligomerized CPT1A and DLD proteins in 293T cells treated with CPT1A OE and transfected with DLD‐320R, followed by grayscale analysis for quantification (*n* = 3). t) Fluorescence images of LX2 cells stained with Mito‐Tracker Green were captured to assess the mitochondrial morphology after treatment with DATs (10 µm) and transfection with CPT1A siRNA or negative control siRNA (*n* = 3). Scale bar: 10 µm. Data are presented as mean ± SD, with *p*‐values calculated using one‐way ANOVA. ns, not significant; *p < 0.05, **p < 0.01.

This study systematically analyzed the impact of DATs intervention on the overall succinylation level of proteins in cells. The results showed that DATs treatment significantly increased the succinylation levels of proteins within the cells, further supporting the occurrence of copper‐induced cell death (**Figure** **4**a). To identify the key enzymes involved in the succinylation of DLD protein, we interfered with the expression of four known succinylation‐modifying enzymes and used immunoprecipitation (IP) experiments to determine that CPT1A is the succinylation modifier of DLD (Figure 4b). Further coimmunoprecipitation (Co‐IP) experiments verified the interaction between CPT1A and DLD proteins and demonstrated that DATs significantly promoted the expression and succinylation modification of DLD by activating CPT1A, while inhibiting its ubiquitination (Figure 4c). Notably, the knockdown of CPT1A significantly weakened DATs‐induced DLD succinylation, suggesting that this modification depends on the succinylation‐modifying function of CPT1A (Figure 4d).

Protein succinylation involves the covalent attachment of succinyl groups to specific lysine residues, significantly altering the protein's conformation, hydrophilicity, and interaction characteristics, thereby promoting protein oligomerization.^[^ ^22^ ^]^ This post‐translational modification not only enhances protein stability and prevents its degradation but also participates in cell signaling, metabolic regulation, and stress responses.^[^ ^23^ ^]^ Moreover, oligomerization plays a crucial role in regulating the activity of key signaling proteins, thus determining the cell's fate in response to environmental stress.^[^ ^22^ ^]^ This study further found that after DATs induced succinylation of DLD protein, the level of oligomerized DLD protein significantly increased (Figure 4e and Figure S4a, Supporting Information). Notably, knockdown of CPT1A expression effectively blocked DLD oligomerization, suggesting that the function of CPT1A is closely related to the formation of DLD oligomers (Figure 4f and Figure S4b, Supporting Information).

To clarify the regulatory role of CPT1A in DLD and its associated protein oligomerization, we analyzed the oligomerization levels of DLD protein, lipoic acid‐modified proteins, and acylated DLAT proteins after CPT1A expression was interfered with. The experimental results showed that CPT1A knockdown significantly reduced the expression of these proteins. Additionally, DATs‐induced oligomerization levels of DLD protein, lipoic acid‐modified proteins, and acylated DLAT proteins were significantly increased. However, after interfering with CPT1A expression, the oligomerization and expression levels of these proteins significantly decreased (Figure 4g,h and Figure S4c,d, Supporting Information). Similarly, through the CCK8 assay to measure cell viability, we further demonstrated that silencing CPT1A expression significantly reversed the decrease in cell viability induced by different concentrations of DATs (Figure S1a, Supporting Information).

Given that previous studies have shown that CPT1A is highly expressed during the pathological process of liver fibrosis, we further explored the relationship between high expression of CPT1A and the inhibition of HSCs viability after DATs treatment. In gradient concentration Elesclomol‐Cu intervention experiments, we observed the upregulation of cuproptosis‐related proteins, confirming the occurrence of copper death. However, the expression level of CPT1A did not show significant changes during this process (Figure 4i and Figure S4e, Supporting Information). Further experiments showed that after interference with CPT1A expression, the expression of DLD protein significantly decreased, but this decrease was significantly reversed after adding Elesclomol‐Cu (Figure 4j and Figure S4f, Supporting Information). Through CCK‐8 assays, we found that Elesclomol‐Cu intervention significantly suppressed the increase in cell viability caused by CPT1A overexpression (Figure S1b, Supporting Information). These results suggest that under copper ion conditions, the overexpression of CPT1A promotes the occurrence of cuproptosis. This phenomenon may be attributed to the targeting effect of cuproptosis on the TCA cycle. CPT1A, as the rate‐limiting enzyme in fatty acid β‐oxidation, plays a key role in maintaining cellular function. Cuproptosis disrupts the TCA cycle, interfering with the final energy supply stage of fatty acid β‐oxidation. This study provides new insights to address the discrepancies reported in previous literature.

Succinylation and ubiquitination are key post‐translational modifications of proteins, and these two modifications may compete at specific sites. Succinylation depends on the succinyl group provided by succinyl‐CoA to regulate protein function, stability, and interactions,^[^ ^20^ ^]^ while ubiquitination involves the covalent attachment of ubiquitin molecules to proteins, marking them for degradation or involvement in signaling processes. When the lysine residues of the same protein serve as targets for both succinylation and, these two modifications may compete with each other, determining the fate of the protein.^[^ ^24^ ^]^ For example, succinylation may hinder the occurrence of ubiquitination, thereby extending the protein's half‐life and preventing its degradation. In contrast, when ubiquitination predominates, the protein is more likely to be tagged for degradation.

Given that previous studies have shown that DATs inhibit the ubiquitin‐mediated degradation of DLD protein, we hypothesized that DATs may involve competition between succinylation and ubiquitination at specific sites during the regulation of DLD protein expression and accumulation. To test this hypothesis, we used a succinylation prediction tool (GPSuc) to predict the potential succinylation sites on DLD protein. The results indicated that multiple potential succinylation modification sites were present on the DLD protein. We then queried the ubiquitination sites of DLD in the ubiquitination site databases (iPTMnet and PhosphoSitePlus) and performed an intersection analysis with the predicted succinylation modification sites. Ultimately, we identified K104, K320, and K410 as overlapping sites (Figure 4k). Further, we transfected LX2 cells with FLAG‐tagged wild‐type DLD (DLD‐WT), DLD‐104R, DLD‐320R, and DLD‐410R overexpression plasmids, and performed IP with FLAG antibody to assess the exogenously expressed DLD. The results showed that overexpression of DLD‐320R significantly reduced the succinylation level of DLD (Figure 4l). When both DLD‐WT or DLD‐320R plasmids and HA‐tagged wild‐type ubiquitin (Ub) plasmids were cotransfected, the results indicated that compared to cells transfected with DLD‐WT, the ubiquitination level of DLD (detected using HA‐tag antibody) was significantly lower in cells transfected with the DLD‐320R plasmid (Figure 4m). These results suggest that K320 is a critical competitive site for succinylation and ubiquitination modification of DLD. Additionally, overexpression of DLD‐320R effectively inhibited DLD oligomerization, revealing the crucial role of the K320 site in the oligomer formation of DLD after CPT1A regulation of its succinylation modification. After transfection with the DLD‐320R overexpression plasmid, the oligomerization levels of lipoic acid‐modified proteins, DLD protein, and DLAT protein were significantly reduced (Figure 4n–p and Figure S4g–i, Supporting Information), further confirming the importance of this mutation in regulating cuproptosis and the oligomerization of these proteins. To explore the effect of the DLD‐320R mutation on DATs treatment, we subjected DLD‐320R mutant cells to DATs intervention. The results showed that compared to the DATs treatment group alone, the DLD‐320R mutation combined with DATs treatment significantly reduced the oligomerization levels and expression of relevant proteins (Figure 4n–p and Figure S4g–i, Supporting Information). To clarify whether CPT1A regulates DLD succinylation modification and its oligomer formation via the K320 site, we mutated the K320 site on DLD in the presence of CPT1A overexpression. The results showed that CPT1A overexpression led to an increase in DLD protein levels, but this effect was significantly reversed after the K320 site mutation, and the expression of DLD protein decreased (Figure 4q and Figure S4j, Supporting Information). These results reveal the central role of the K320 site in the functional regulation of DLD mediated by CPT1A.

Through CCK8 assays to measure cell viability, the results showed that after treatment with different concentrations of DATs, the cell survival rate in the K320R group was significantly higher than that in the WT and Negative groups, indicating that the K320R mutant effectively reversed the decrease in cell viability induced by DATs (Figure S1c, Supporting Information). Furthermore, we further demonstrated that overexpression of DLD‐320R significantly reversed the decrease in cell viability induced by both DATs and Elesclomol‐Cu (Figure S1d, Supporting Information). To further confirm the central role of the K320 site in CPT1A‐mediated regulation of DLD, we performed similar experiments in 293T cells, which supported this conclusion (Figure 4r,s and Figure S4k, Supporting Information).

Given that DATs have been shown to increase mitochondrial numbers in cells, we further investigated whether this process is related to CPT1A expression. Using a mitochondrial fluorescent probe, we found that interference with CPT1A expression significantly reduced the number of mitochondria in the cells. Notably, DATs‐induced increases in mitochondrial numbers were significantly reversed after interference with CPT1A expression (Figure 4t). These results further confirm the crucial role of CPT1A in the copper death process. Since cuproptosis is more likely to occur in cells reliant on mitochondrial respiration, CPT1A not only promotes DLD succinylation and enhances DLD expression but also provides a favorable metabolic environment for the occurrence of copper death.

In summary, DATs enhance CPT1A expression, promote the succinylation modification of DLD protein at the K320 site, and inhibit its ubiquitination, thereby stabilizing its expression levels. This mechanism plays a key regulatory role in copper‐induced cell death

### 1.5 Inhibition of Lipophagy Promotes DATs‐Regulated CPT1A‐Induced Copper Death in HSCs

In the Disse space of the liver, HSCs are rich in lipid droplets in their quiescent state, which serve as the primary storage organ for neutral lipids, maintaining the balance and homeostasis of intracellular lipid metabolism.^[^ ^25^ ^]^ However, during liver fibrosis, HSCs are abnormally activated under the stimulation of oxidative stress and inflammatory factors, transforming into an activated state with a myofibroblast‐like phenotype. In this process, the number of lipid droplets significantly decreases or disappears, a phenomenon known as “lipophagy”.^[^ ^5^ ^]^ Lipophagy refers to the degradation of lipid droplets through the autophagic pathway, converting stored lipids into energy and materials required for metabolism.^[^ ^26^ ^]^ In addition to supporting metabolic needs, lipophagy may also play a key role in maintaining the dynamic balance of intracellular copper ions.

Although the role of lipophagy in lipid metabolism has been reported, its specific relationship with cuproptosis has not been systematically studied. The increase in lipid droplets may promote the accumulation of metal ions and lead to copper ion imbalance, which in turn triggers oxidative stress and cell damage. This suggests that the level of lipophagy may play a key role in the regulation of cuproptosis.

**Figure 5:**
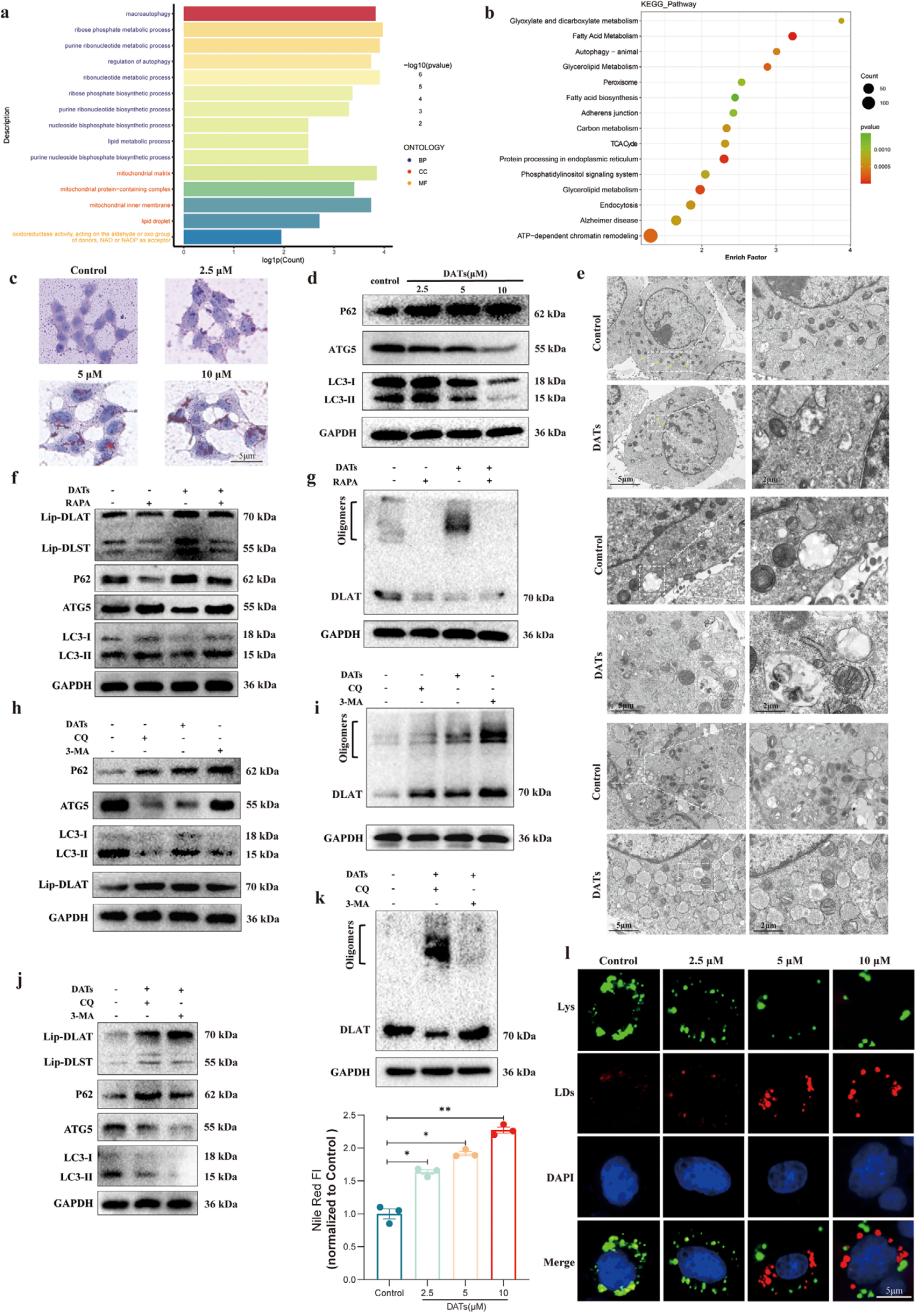
Inhibition of lipophagy promotes DATs‐regulated CPT1A‐induced copper death in HSCs. a,b) Co‐IP‐MS was performed to systematically identify the protein interaction network associated with CPT1A in LX2 cells, followed by GO and KEGG analysis (*n* = 3). c) Oil Red O staining was conducted to assess the lipid droplet content in LX2 cells treated with DATs (0–10 µm) for 24 h (*n* = 3). Scale bar: 5 µm. d) Immunoblotting was used to evaluate the expression levels of ATG5, LC3‐II, and p62 in LX2 cells treated with DATs (0–10 µm) for 24 h, followed by grayscale analysis for quantification (*n* = 3). e) Transmission electron microscopy images of LX2 cells treated with DATs (10 µm) for 24 h were captured. The right image is an enlarged view of the boxed region from the left image. Scale bar: 5 µm. f) Immunoblotting was performed to evaluate the expression levels of Lip‐DLST, Lip‐DLAT, P62, ATG5, and LC3 proteins in LX2 cells treated with DATs (10 µm) and Rapamycin intervention, followed by grayscale analysis for quantification (*n* = 3). g) Immunoblotting was conducted to assess the expression levels of oligomerized DLAT protein in LX2 cells treated with DATs (10 µm) and rapamycin intervention, with grayscale analysis for quantification (*n* = 3). h,i) Immunoblotting was performed to assess the expression levels of Lip‐DLST, Lip‐DLAT, P62, ATG5, LC3, and oligomerized DLAT proteins in LX2 cells treated with DATs (10 µm), in the presence or absence of CQ (5 µm) or 3‐MA (10 µm) for 24 h, followed by grayscale analysis for quantification (*n* = 3). j,k) Immunoblotting was conducted to evaluate the expression levels of Lip‐DLST, Lip‐DLAT, P62, ATG5, LC3, and oligomerized DLAT protein in LX2 cells treated with DATs (10 µm), in the presence or absence of CQ (5 µm) or 3‐MA (10 µm) for 24 h, followed by grayscale analysis for quantification (*n* = 3). l) Immunofluorescence images were obtained to observe the colocalization of Lysosomes (green) and LDs (red) in LX2 cells treated with DATs (0–10 µm) for 24 h (*n* = 3). Scale bar: 5 µm. Data are presented as mean ± SD, with *p*‐values calculated using one‐way ANOVA. ns, not significant; *p < 0.05, **p < 0.01.

In our proteomic analysis, we found that DATs treatment significantly altered pathways in LX2 cells related to cuproptosis, GSH metabolism, fatty acid metabolism, and lipophagy. Since CPT1A is a key enzyme in fatty acid metabolism, we hypothesized that it might play an important role in the process of lipophagy. To further validate this hypothesis, we systematically identified the protein network interacting with CPT1A using co‐immunoprecipitation combined with mass spectrometry (Co‐IP‐MS). The analysis revealed that CPT1A interacts with various key proteins involved in mitochondrial metabolism, lipid metabolism, and cellular stress responses. GO enrichment analysis showed that these interacting proteins were primarily enriched in key processes such as autophagy (macroautophagy), lipid droplet metabolism, and the mitochondrial inner membrane (**Figure** **5**a). KEGG pathway analysis further revealed that these proteins significantly participate in metabolic pathways such as fatty acid metabolism, autophagy, triglyceride metabolism, and peroxisome pathways (Figure 5b). Notably, the enrichment of autophagy‐ and lipid droplet metabolism‐related proteins suggests that CPT1A may play an important role in the lipophagy process and influence the occurrence of cuproptosis by regulating lipid homeostasis.

To explore the effect of DATs on lipophagy, we used Oil Red O staining to detect changes in lipid droplet content in LX2 cells after DATs treatment. The results showed that DATs treatment significantly increased intracellular lipid droplet accumulation, suggesting that it inhibited the degradation of lipid droplets (Figure 5c). Additionally, Western blot analysis further revealed that with increasing concentrations of DATs, the expression levels of autophagy‐related markers such as Autophagy‐related protein 5 (ATG5), Microtubule‐associated protein 1A/1B‐light chain 3 (LC3‐II/I), and Sequestosome‐1 (p62) changed significantly, indicating that DATs significantly inhibited autophagic activity in LX2 cells (Figure 5d and Figure S5a, Supporting Information). Next, we used transmission electron microscopy to observe changes in the distribution of lipid droplets in autophagosomes and lysosomes after DATs treatment, and performed statistical quantification of the results to further verify the relationship between changes in autophagy levels and lipid droplet accumulation. The results showed that after DATs treatment, the number of lysosomes containing lipid droplets significantly decreased, the number of autophagosomes also decreased, and the number of lipid droplets in the endoplasmic reticulum increased significantly (Figure 5e and Figure S5b,c, Supporting Information). To further investigate the role of lipophagy in regulating cuproptosis, we treated cells with the Rapamycin and DATs, either separately or in combination. The experimental results showed that after autophagy activation, the expression of key cuproptosis markers—lipoic acid‐modified proteins—was significantly reduced, and the oligomerization level of DLAT protein also decreased, indicating that the cuproptosis process was inhibited (Figure 5f,g and Figure S5d,e, Supporting Information). When both treatments were combined, the inhibitory effect of DATs on autophagy and its induced cuproptosis effect were successfully reversed. These results suggest that lipophagy may play an important regulatory role in cuproptosis by modulating lipoic acid modification and DLAT protein oligomerization. Correspondingly, we further treated cells with the autophagy inhibitors (CQ or 3‐MA), either alone or in combination with DATs. The results showed that autophagy inhibition led to a significant increase in the expression of lipoic acid‐modified proteins and DLAT oligomerization levels, and cuproptosis was significantly enhanced. Moreover, when combined with DATs, this effect on cuproptosis was further amplified (Figure 5h–k and Figure S5f–i, Supporting Information). These findings indicate that the inhibition of lipophagy promotes copper ion accumulation and DLAT oligomerization, ultimately significantly activating cuproptosis.

To further explore the specific regulatory mechanism of DATs on lipophagy, we used Nile Red dye to label lipid droplets and employed Lysotracker Green dye for lysosomal staining. We then observed the colocalization of lipid droplets and lysosomes under different concentrations of DATs treatment using confocal laser scanning microscopy. The degree of colocalization between the two fluorescent markers was quantitatively analyzed using the Manders’ coefficient. As the DATs concentration increases, the degree of colocalization between lipid droplets and lysosomes gradually decreases, while the number of lipid droplets in the cells significantly increases (Figure 5l). To determine the cause of lipid droplet accumulation, we further used DATs in combination with the autophagy activator rapamycin and the lipolysis activator Lys‐γ3‐MSH (human) Trifluoroacetic Acid (TFA) to analyze changes in lipid droplet numbers. The results showed that DATs treatment could not reverse the reduction of lipid droplets caused by the lipolysis activator, but it effectively reversed the decrease in lipid droplet numbers induced by the autophagy activator (Figure S5j, Supporting Information). This suggests that DATs primarily promote lipid droplet accumulation by inhibiting lipophagy, rather than interfering with lipolysis. In summary, DATs enhance the effect of cuproptosis by inhibiting lipophagy, which promotes the expression of lipoic acid‐modified proteins and the oligomerization of DLAT, thereby amplifying the cuproptosis effect.

### 1.6 MAMs Structure Key in DATs‐Regulated RAB18 Inhibition of Lipophagy and HSCs Cuproptosis

**Figure 6:**
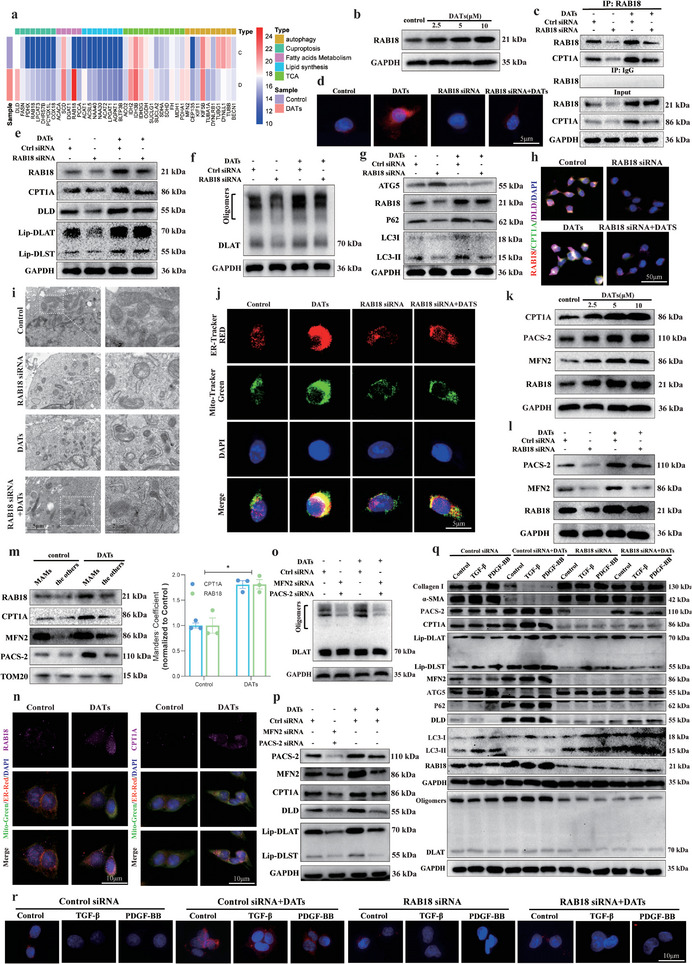
MAMs structure key in DATs‐regulated RAB18 inhibition of lipophagy and HSCs cuproptosis. a) Co‐IP‐MS was performed to systematically identify the protein interaction network associated with CPT1A in LX2 cells treated with DATs (10 µm), followed by a heatmap representation (*n* = 3). b) Immunoblotting was performed to evaluate the expression levels of RAB18 protein in LX2 cells treated with DATs (0–10 µm) for 24 h and the results were quantified using grayscale analysis (*n* = 3). c) Co‐IP assays were conducted to assess the interaction between CPT1A and RAB18 in LX2 cells transfected with CPT1A siRNA or negative control siRNA and treated with DATs (10 µm) for 24 h (*n* = 3). d) Fluorescence images were captured to assess the number of lipid droplets in LX2 cells treated with DATs (10 µm) for 24 h and transfected with RAB18 siRNA or negative control siRNA (*n* = 3). Scale bar: 5 µm. e) Immunoblotting was performed to evaluate the expression levels of CPT1A, DLD, Lip‐DLST, and Lip‐DLAT proteins in LX2 cells treated with DATs (10 µm) for 24 h and transfected with RAB18 siRNA or negative control siRNA, followed by grayscale analysis for quantification (*n* = 3). f) Immunoblotting was used to assess the expression levels of oligomerized DLAT protein in LX2 cells treated with DATs (10 µm) for 24 h and transfected with RAB18 siRNA or negative control siRNA, followed by grayscale analysis for quantification (*n* = 3). g) Immunoblotting was conducted to evaluate the expression levels of ATG5, P62, and LC‐3 proteins in LX2 cells treated with DATs (10 µm) for 24 h and transfected with RAB18 siRNA or negative control siRNA, followed by grayscale analysis for quantification (*n* = 3). h) Fluorescence images were obtained to observe the colocalization of RAB18, DLD, and CPT1A in LX2 cells treated with DATs (10 µm) for 24 h and transfected with RAB18 siRNA or negative control siRNA (*n* = 3). Scale bar: 50 µm. i) Transmission electron microscopy images of LX2 cells treated with DATs (10 µm) for 24 h and transfected with RAB18 siRNA or negative control siRNA were captured. The right image is an enlarged view of the boxed region from the left image. Scale bar: 5 µm. j) Immunofluorescence images were taken to observe the colocalization of Mito‐Tracker (green) and Endoplasmic Reticulum (ER)‐Tracker (red) in LX2 cells treated with DATs (10 µm) for 24 h and transfected with RAB18 siRNA or negative control siRNA (*n* = 3). Scale bar: 5 µm. k) Immunoblotting was performed to assess the expression levels of CPT1A, PACS‐2, MFN2, and RAB18 proteins in LX2 cells treated with DATs (0–10 µm) for 24 h and the results were quantified using grayscale analysis (*n* = 3). l) Immunoblotting was conducted to evaluate the expression levels of PACS‐2 and MFN2 proteins in LX2 cells treated with DATs (10 µm) for 24 h and transfected with RAB18 siRNA or negative control siRNA (*n* = 3). m) Immunoblotting was performed to assess the expression levels of CPT1A, PACS‐2, MFN2, and RAB18 proteins in MAMs structures isolated by gradient centrifugation from LX2 cells treated with DATs (0–10 µm) for 24 h (*n* = 3). n) Fluorescence images were obtained to observe the colocalization of RAB18 (purple), CPT1A (purple), Mito‐Tracker (green), and ER‐Tracker (red) in LX2 cells treated with DATs (10 µm) for 24 h (*n* = 3). Scale bar: 5 µm. o) Immunoblotting was performed to evaluate the expression levels of oligomerized DLAT protein in LX2 cells treated with DATs (10 µm) for 24 h and transfected with MFN2/PACS‐2 siRNA or negative control siRNA (*n* = 3). p) Immunoblotting was conducted to assess the expression levels of CPT1A, PACS‐2, MFN2, DLD, Lip‐DLST, and Lip‐DLAT proteins in LX2 cells treated with DATs (10 µm) for 24 h and transfected with MFN2/PACS‐2 siRNA or negative control siRNA (*n* = 3). q) Immunoblotting was performed to evaluate the expression levels of RAB18, ATG5, P62, LC‐3, CPT1A, PACS‐2, MFN2, DLD, α‐Smooth Muscle Actin (SMA), Collagen I, Lip‐DLST, Lip‐DLAT, and oligomerized DLAT proteins in LX2 cells activated with TGF‐β/PDGF‐BB and treated with DATs (10 µm) for 24 h (*n* = 3). r) Fluorescence images were taken to assess the number of lipid droplets in LX2 cells activated with Transforming Growth Factor‐β (TGF‐β)/Platelet‐Derived Growth Factor‐BB (PDGF‐BB) HEPES 4‐(2‐Hydroxyethyl) piperazine‐1‐ethanesulfonic acid and treated with DATs (10 µm) for 24 h, in the presence or absence of RAB18 siRNA or negative control siRNA (*n* = 3). Scale bar: 5 µm. Data are presented as mean ± SD, with *p*‐values calculated using one‐way ANOVA. ns, not significant; *p < 0.05, **p < 0.01.

To further investigate the key targets of DATs in inhibiting lipophagy and inducing cuproptosis in HSCs, we first used quantitative proteomics to identify proteins that may interact with CPT1A after DATs treatment in LX2 cells. We selected the top 100 proteins ranked by interaction scores as preliminary candidates and then performed functional enrichment analysis on these proteins related to lipophagy and fatty acid metabolism pathways. The analysis revealed that RAB18 was notably enriched, especially in the signaling pathways related to lipophagy and fatty acid metabolism, showing a significant association (**Figure** **6**a). After DATs treatment, the expression level of RAB18 significantly increased, suggesting that it may be involved in the inhibition of lipophagy and the regulation of fatty acid metabolism through its interaction with CPT1A. To further verify this effect, we measured the expression levels of RAB18 under different concentrations of DATs. The results indicated that its expression increased significantly with higher drug concentrations (Figure 6b and Figure S6a, Supporting Information).

Additionally, the analysis revealed that after DATs treatment, the expression of the lipid droplet synthesis‐related protein Acetyl‐CoA Acetyltransferase 1 (ACAT1) significantly increased, likely due to the upregulation of CPT1A, which in turn elevated ACAT1 expression levels. High expression of ACAT1 has been shown to promote lipid droplet synthesis.^[^ ^27^ ^]^ To further verify the relationship between the two, we interfered with CPT1A expression and observed a corresponding decrease in ACAT1 expression, thus confirming the critical role of CPT1A in regulating ACAT1 expression (Figure S10a, Supporting Information). This finding explains the discrepancies in the current literature regarding the high expression of CPT1A and changes in lipid droplet numbers, providing new mechanistic insights.

Through Co‐IP experiments, we confirmed the interaction between RAB18 and CPT1A (Figure 6c). Further interference with RAB18 expression showed changes in the number of lipid droplets (Figure 6d), as well as the expression levels of autophagy‐related proteins (such as LC3, P62, and ATG5) and cuproptosis markers (such as lipoic acid‐modified proteins and oligomerized DLAT proteins) (Figure 6e–g and Figure S6b–d, Supporting Information). The results showed that downregulation of RAB18 significantly reduced the number of lipid droplets, activated autophagy, and inhibited the progression of cuproptosis. The effects of DATs on lipophagy inhibition and cuproptosis induction were significantly reversed after interference with RAB18 expression, indicating the critical role of RAB18 in DATs‐mediated lipophagy inhibition and cuproptosis. Using multiplex fluorescence labeling, we further examined the changes in CPT1A and DLD protein expression after knockdown of RAB18. The results showed that interference with RAB18 expression significantly reduced the fluorescence expression levels of CPT1A and DLD, and DATs treatment failed to restore the expression of these two proteins (Figure 6h). These results further confirm the central role of RAB18 in regulating the expression of CPT1A and DLD proteins.

Combined with bioinformatics analysis, we found that RAB18 is localized to the endoplasmic reticulum, while CPT1A is located on the outer mitochondrial membrane, suggesting that the interaction between the two may mediate inter‐organelle communication. Mitochondria‐associated membranes (MAMs), as bridges between the mitochondria and endoplasmic reticulum, are known to play an important role in regulating lipid metabolism and autophagy.^[^ ^28^ ^]^ MAMs, as key signaling platforms, facilitate the exchange of materials and metabolic reactions between these two organelles.^[^ ^29^ ^]^ However, the relationship between MAMs and cuproptosis has not been fully explored. Based on our experimental results, we propose that the interaction between RAB18 and CPT1A may play a key role in regulating lipophagy and cuproptosis by promoting the formation of MAMs. Transmission electron microscopy revealed that, compared to the DATs‐only group, the DATs‐induced increase in MAMs structures was significantly reversed after interference with RAB18 expression (Figure 6i). Using fluorescence probes for mitochondria and the endoplasmic reticulum, we observed their colocalization through confocal laser scanning microscopy. The results showed that after interference with RAB18, the colocalization of mitochondria and the endoplasmic reticulum significantly decreased, and the DATs‐induced increase in fluorescence colocalization was significantly reversed after RAB18 expression was interfered with (Figure 6j).

To further investigate the role of RAB18 in the formation of MAMs structures, we measured the expression levels of MAMs‐related structural proteins MFN2 and PACS‐2 after DATs treatment with gradient dosing. The results showed that with increasing drug concentrations, the protein expression of Mitofusin 2 (MFN2) and Phosphofurin Acidic Cluster Sorting Protein 2 (PACS‐2) increased in a dose‐dependent manner (Figure 6k and Figure S6e, Supporting Information). After interference with RAB18 expression, the increase in MAMs markers (such as MFN2 and PACS‐2) induced by DATs was significantly reversed (Figure 6l and Figure S6f, Supporting Information). To further confirm whether RAB18 is enriched in the MAMs structure, we separated MAMs by ultracentrifugation and performed protein analysis. In this process, the mitochondrial outer membrane protein TOM20 was used as a reference. Western blot results showed that, compared to the control group, RAB18 and CPT1A were significantly enriched in MAMs, further supporting the functional localization of RAB18 in relation to MAMs (Figure 6m and Figure S6g, Supporting Information). Additionally, confocal laser scanning microscopy confirmed that the colocalization of RAB18 and CPT1A in MAMs structures increased, further supporting the role of RAB18 in MAMs formation (Figure 6n).

Previous studies have shown that downregulation of MFN2 and PACS‐2 expression significantly disrupts the structure of MAMs.^[^ ^30^ ^]^ To further explore the specific function of MAMs in lipophagy and cuproptosis, we interfered with the expression levels of MAMs markers MFN2 and PACS‐2. The results showed that disruption of MAMs structure significantly activated lipophagy and inhibited cuproptosis (Figure 6o,p and Figure S6h–j, Supporting Information).

To further investigate the effect of RAB18 expression on lipophagy and cuproptosis in HSCs, we used common HSCs activators, TGF‐β and PDGF‐BB, and compared the effects of RAB18 interference on lipophagy and cuproptosis with and without activator treatment. The results showed that, regardless of activator treatment, interference with RAB18 expression led to the activation of lipophagy and inhibition of cuproptosis. Notably, the DATs‐induced inhibition of lipophagy and cuproptosis was significantly reversed after interference with RAB18 expression, and the use or nonuse of activators did not significantly alter this process (Figure 6q,r and Figure S6k, Supporting Information).

In this study, we further investigated the mechanism by which RAB18 regulates lipid droplet accumulation in LX2 cells, focusing on its role in modulating lipophagy rather than lipolysis. To this end, following RAB18 knockdown, we treated the cells with either an autophagy inhibitor(CQ) or a lipolysis inhibitor (Atglistatin) to assess their effects on intracellular lipid droplet content. The results demonstrated that Atglistatin treatment did not significantly restore lipid droplet levels, whereas inhibition of autophagy led to a marked increase in lipid droplet accumulation (Figure S10b, Supporting Information), suggesting that RAB18 modulates lipid droplet homeostasis primarily through the suppression of lipophagy. Subsequently, LX2 cells were treated with Atglistatin and/or DATs. DATs treatment alone did not alter the expression of the lipolysis‐associated markers Adipose Triglyceride Lipase (ATGL) and Hormone‐Sensitive Lipase (HSL). Furthermore, the Atglistatin‐induced downregulation of ATGL and HSL protein levels remained unaffected by DATs cotreatment (Figure S10c, Supporting Information).

Additionally, We used the CCK8 assay to measure cell viability in order to further investigate the important role of RAB18 in regulating HSCs activity. The results showed that after treatment with different concentrations of DATs, the cell viability in the RAB18 siRNA group was significantly lower than in the WT and Negative groups, indicating that interference with RAB18 expression effectively reversed the decrease in cell viability induced by DATs (Figure S1e, Supporting Information). Additionally, we further demonstrated that interference with RAB18 expression significantly reversed the decrease in cell viability induced by both DATs and Elesclomol‐Cu (Figure S1f, Supporting Information). To further confirm the central role of RAB18 in regulating lipophagy and cuproptosis, we conducted similar experiments in 293T cells, and the results supported this conclusion (Figure S1j,k, Supporting Information). In 293T cells, by interfering with or overexpressing RAB18, we measured changes in cell viability as well as the expression of CPT1A, DLD, autophagy, and cuproptosis‐related proteins. The results showed that interference with RAB18 led to a decrease in the expression of CPT1A and DLD proteins, while overexpression of RAB18 increased their expression. At the same time, autophagy was activated, and cuproptosis was inhibited (Figure S1l, Supporting Information). These results reveal the crucial role of RAB18 in regulating the processes of autophagy and cuproptosis.

### 1.7 DATs Induce RAB18 Phase Separation to Recruit CPT1A Mediate MAMs Formation Inhibit Lipophagy and Trigger Cuproptosis in HSCs

**Figure 7:**
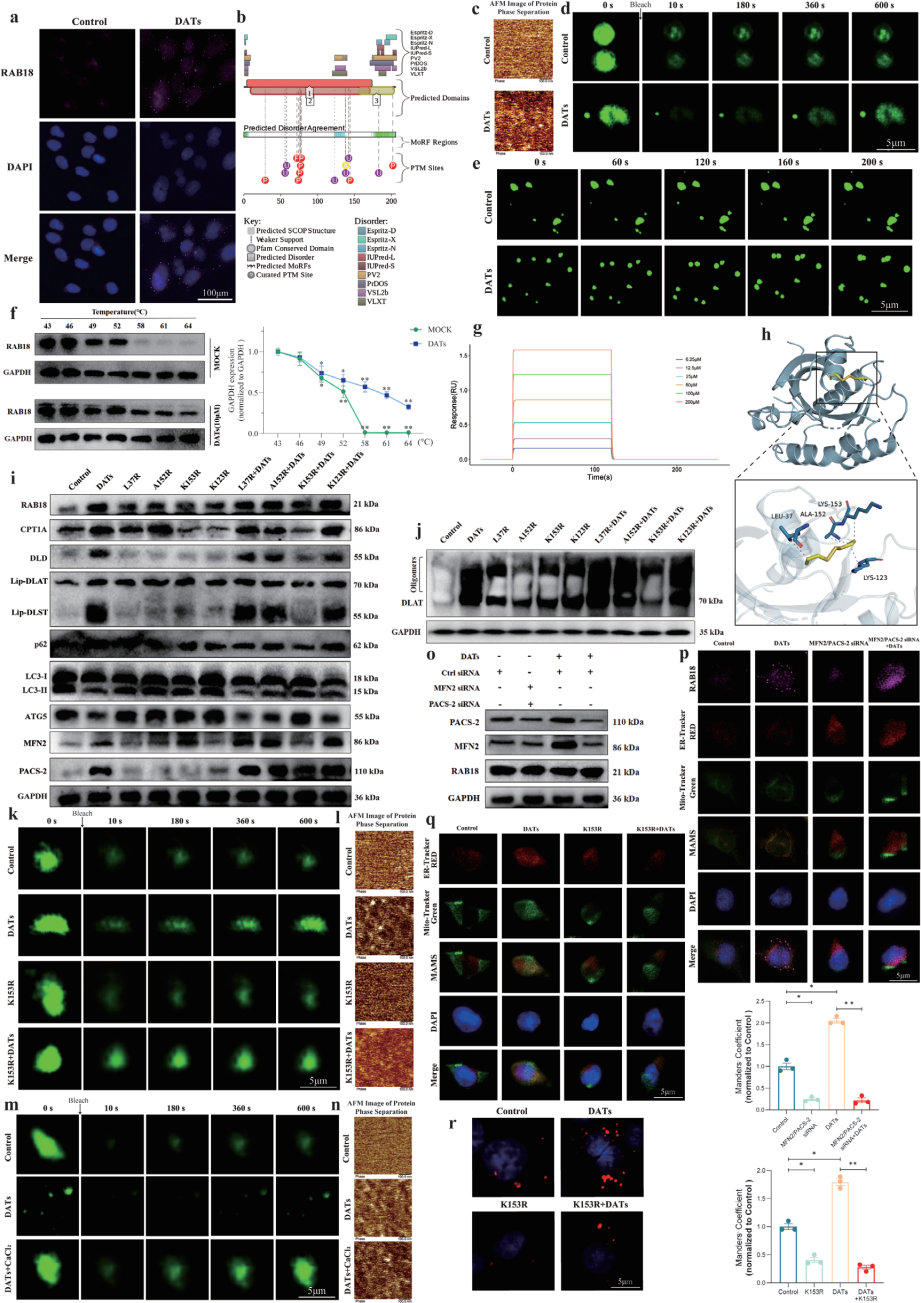
DATs induce RAB18 phase separation to recruit CPT1A mediate MAMs formation inhibit lipophagy and trigger cuproptosis in HSCs. a) Immunofluorescence images of RAB18 in LX2 cells treated with DATs (10 µm) for 24 h (*n* = 3). Scale bar: 100 µm. b) D2P2 database prediction indicates significant disordered regions in RAB18 protein, suggesting its potential for phase separation. c) AFM was used to assess the phase separation behavior of purified RAB18 protein treated with DATs (10 µm) (*n* = 3). d) FRAP experiments were conducted to assess the fluorescence recovery rate of the RAB18 purified protein treated with DATs (10 µm) (*n* = 3). e) Droplet formation assays were conducted to assess the droplet fusion phenomenon of the purified RAB18 protein treated with DATs (10 µm) (*n* = 3). f) Cellular thermal shift assay (CETSA) was performed to evaluate the binding affinity between DATs and RAB18 protein in LX2 cells treated with DATs (10 µm) for 24 h (*n* = 3). g) Surface plasmon resonance (SPR) was used to assess the binding affinity between DATs and purified RAB18 protein (*n* = 3). h) Molecular docking analysis was conducted to evaluate the potential binding sites of RAB18 protein with DATs, specifically LEU‐37, ALA‐152, LYS‐153, and LYS‐123. i,j) Immunoblotting was used to assess the expression levels of RAB18, ATG5, P62, LC‐3, CPT1A, PACS‐2, MFN2, DLD, Lip‐DLST, Lip‐DLAT, and oligomerized DLAT proteins in LX2 cells treated with DATs (10 µm) for 24 h and RAB18 amino acid point mutations, followed by grayscale analysis (*n* = 3). k) FRAP experiments were conducted to assess the fluorescence recovery rate of the RAB18‐K153R purified protein treated with DATs (10 µm) (*n* = 3). l) AFM was used to assess the phase separation behavior of purified RAB18‐K153R protein treated with DATs (10 µm) (*n* = 3). m) FRAP experiments were conducted to assess the fluorescence recovery rate of RAB18 purified protein treated with DATs (10 µm) and CaCl_2_ intervention (*n* = 3). n) AFM was used to assess the phase separation behavior of RAB18 purified protein treated with DATs (10 µm) and CaCl_2_ intervention (*n* = 3). o) Immunoblotting was performed to evaluate the expression levels of MFN2, PACS‐2, and RAB18 proteins in LX2 cells treated with DATs (10 µm) and transfected with MFN2/PACS‐2 siRNA or negative control siRNA (*n* = 3). p) Immunofluorescence images and colocalization analysis of RAB18 (purple), Mito‐Tracker (green), and ER‐Tracker (red) in LX2 cells treated with DATs (10 µm) for 24 h and transfected with MFN2/PACS‐2 siRNA or negative control siRNA (*n* = 3). Scale bar: 5 µm. q) Colocalization analysis of Mito‐Tracker (green) and ER‐Tracker (red) in LX2 cells treated with DATs (10 µm) for 24 h and transfected with RAB18‐K153R or negative control siRNA, followed by immunofluorescence imaging and colocalization analysis (*n* = 3). r) Fluorescence images showing the number of lipid droplets in LX2 cells treated with DATs (10 µm) for 24 h and transfected with RAB18‐K153R or negative control siRNA (*n* = 3). Scale bar: 100 µm. Data are presented as mean ± SD, with *p*‐values calculated using one‐way ANOVA. ns, not significant; *p < 0.05, **p < 0.01.

Through laser confocal microscopy, we observed RAB18 fluorescence staining in cells, and the results showed a distinct droplet‐like distribution of RAB18 protein, suggesting that LLPS may have occurred (**Figure** **7**a). LLPS refers to the process in which specific proteins or nucleic acids spontaneously aggregate through weak interactions to form membrane‐less droplets, a phenomenon similar to oil–water separation.^[^ ^31^ ^]^ This process creates local high‐concentration microenvironments within the cell, which in turn regulate various biological reactions. Intrinsically disordered regions (IDRs) of proteins are key areas that drive LLPS, facilitating protein aggregation and droplet formation through multivalent weak interactions.^[^ ^32^ ^]^ Additionally, the flexibility of these disordered regions allows proteins to rapidly respond to changes in cellular signals, thereby regulating complex intracellular processes.^[^ ^33^ ^]^

In liver disease research, LLPS has been shown to play an important regulatory role in lipid metabolism and cellular stress responses.^[^ ^34^ ^]^ When phase separation is imbalanced, IDRs may lead to abnormal lipid droplet aggregation or inhibition of autophagy, ultimately disrupting liver cell function and accelerating disease progression. Through analysis using the D2P2 database, we predicted that RAB18 protein contains significant disordered regions, indicating its potential ability to undergo phase separation (Figure 7b).

To further validate this prediction, we first captured the phase diagram of RAB18 protein using atomic force microscopy (AFM) to observe its phase separation in a simulated cellular environment. RAB18 protein was dissolved in 50 mm HEPES buffer (pH 7.4) and 150 mm NaCl, with a final protein concentration of 50 µm. AFM results showed that, compared to the control group, the RAB18 protein droplets formed in the DATs‐treated group were more pronounced, with significant phase separation occurring (Figure 7c).

Additionally, we conducted a fluorescence recovery after photobleaching (FRAP)experiment. In this experiment, RAB18 protein was at a final concentration of 50 µm, dissolved in 50 mm 4‐(2‐Hydroxyethyl) piperazine‐1‐ethanesulfonic acid (HEPES) (pH 7.4) and 150 mm NaCl buffer to simulate physiological conditions. Under the action of DATs, RAB18 protein formed droplet‐like structures. To monitor their liquid‐like properties, we performed FRAP with an excitation wavelength of 488 nm and an emission wavelength of 510 nm, using a bleaching time of 0.5 s and real‐time tracking of the recovery of the fluorescence signal after bleaching. Compared to the control group, the RAB18 droplets in the DATs‐treated group showed significant fluorescence recovery after bleaching, with a recovery percentage of ≈80%, while the control group showed slower recovery. This indicates that DATs effectively induce RAB18 droplet formation with typical liquid‐like properties of phase separation (Figure 7d). Furthermore, we observed droplet fusion under the microscope, further supporting the occurrence of LLPS of RAB18 under DATs induction (Figure 7e).

We further evaluated the binding affinity between DATs and RAB18 using Cellular Thermal Shift Assay (CETSA). The results showed that DATs exhibit a strong affinity for RAB18, effectively binding to and stabilizing the conformation of RAB18 (Figure 7f). Subsequently, we employed surface plasmon resonance (SPR) technology to measure the affinity between DATs and RAB18, and the results similarly indicated a high binding affinity between the two (Figure 7g). To gain a deeper understanding of the specific binding mechanism between RAB18 and DATs, we performed molecular docking to analyze the binding sites of RAB18 protein with DATs. The results revealed four potential binding sites for RAB18 and DATs, namely LEU‐37, ALA‐152, LYS‐153, and LYS‐123 (Figure 7h).

To investigate the specific roles of these binding sites in RAB18's regulation of lipophagy and cuproptosis, we conducted site‐directed mutagenesis on these four sites. Functional analysis of the mutated proteins showed that mutations at LEU‐37, ALA‐152, and LYS‐123 did not significantly affect the DATs‐induced inhibition of lipophagy and cuproptosis, suggesting that these sites play a limited role in the interaction between RAB18 and DATs. In contrast, mutation of LYS‐153 significantly reversed the DATs‐induced inhibition of lipophagy and cuproptosis, indicating that LYS‐153 plays a critical role in the binding of DATs to RAB18 and in mediating its biological effects (Figure 7I,j and Figure S7a, Supporting Information).

We further constructed a fluorescently labeled RAB18 protein with the LYS‐153 site mutation and used AFM and FRAP experiments to systematically explore the effect of DATs on RAB18 protein phase separation. The experimental results showed that the LYS‐153 mutation significantly weakened the induction of RAB18 phase separation by DATs, with a noticeable decrease in the fluorescence recovery after bleaching (Figure 7k). Furthermore, the AFM imaging results were consistent with the FRAP experiment, both showing that the induction of RAB18 phase separation by DATs was significantly reversed after the LYS‐153 mutation (Figure 7l).

In our previous research, DATs significantly promoted the formation of MAMs structures, with RAB18 protein localized in these structures. MAMs are key contact regions between the mitochondria and the endoplasmic reticulum, playing a crucial role in regulating cellular calcium homeostasis, lipid metabolism, and signal transduction.^[^ ^30^ ^]^ MAMs structures are enriched with calcium ions, which are released from the endoplasmic reticulum into the mitochondria to participate in the regulation of intracellular calcium homeostasis.^[^ ^35^ ^]^ Metal ions, by modulating electrostatic interactions between proteins, affect the formation and stability of LLPS.^[^ ^36^ ^]^ Therefore, we speculate that the calcium ions in MAMs can enhance the interaction between proteins, increase the local concentration of proteins, and promote droplet formation, thereby further regulating various cellular processes, including lipophagy.

To systematically study the effects of MAMs structure and calcium ions on RAB18 phase separation, we employed AFM and FRAP experiments. AFM imaging via phase diagrams visually demonstrated the formation of RAB18 protein droplets mediated by calcium ions, while the FRAP experiment further confirmed the crucial role of calcium ions in promoting RAB18 protein phase separation. The results showed that at high calcium ion concentrations (1 µm), the phase separation effect of RAB18 was significantly enhanced (Figure 7m,n), suggesting that calcium ions in the MAMs structure promote DATs‐induced RAB18 phase separation by strengthening the interaction between RAB18 proteins. This not only indicates the involvement of calcium ions in regulating the MAMs structure but also highlights their potential impact on cellular functions, such as lipophagy, by modulating RAB18 phase separation.

Subsequently, when we knocked down the expression of MAMs markers PACS‐2 and MFN2, although there was no significant change in the protein expression levels of RAB18 (Figure 7o and Figure S7c, Supporting Information), the formation of fluorescent droplets was significantly reduced (Figure 7p). This suggests that RAB18 phase separation is dependent on a specific MAMs environment. Therefore, the MAMs structure and calcium ions play a key role in RAB18 phase separation, with calcium ions directly promoting the interaction between RAB18 proteins and enhancing the occurrence of phase separation.

To further explore the role of the LYS‐153 site in DATs‐induced MAMs structure formation, lipophagy inhibition, and cuproptosis, we performed mutation analysis at the LYS‐153 site. Laser confocal microscopy results showed that after the LYS‐153 mutation, the DATs‐induced MAMs structure formation was significantly reversed (Figure 7q). Nile Red lipid droplet staining further validated this finding: the LYS‐153 mutation significantly abolished the inhibition of lipophagy by DATs on RAB18, confirming that LYS‐153 plays a key role in DATs‐regulated RAB18 phase separation and its associated functions (Figure 7r).

Additionally, laser confocal microscopy analysis showed that the colocalization of RAB18 with CPT1A and lipid droplets significantly increased (Figure S10d, Supporting Information), which may be related to the protein recruitment mechanism mediated by phase separation. During phase separation, proteins aggregate through multivalent weak interactions, forming dynamic and high‐concentration droplet‐like structures. These droplets not only provide RAB18 with a physically isolated microenvironment but may also serve as directional recruitment sites for other functionally relevant proteins. The phase‐separated droplets of RAB18 may facilitate the recruitment of functional proteins like CPT1A by forming this highly organized structure, and through interactions between proteins, they collaboratively regulate the lipophagy process. It may also attract other yet‐to‐be‐identified proteins into the droplets, further expanding its functional network.

RAB18, by phase‐separating and encapsulating lipid droplets, inhibits their autophagy, and this unique recruitment effect of phase separation promotes the assembly of CPT1A. Their interaction drives the formation and stabilization of MAMs structures. Since MAMs structures are enriched with calcium ions, the stability of these structures further enhances the DATs‐induced RAB18 phase separation, creating a positive feedback loop that amplifies the biological effects of phase separation, further regulating the processes of lipophagy and cuproptosis. In conclusion, LYS‐153 is a key site for DATs to regulate RAB18 expression, induce its phase separation, and inhibit lipophagy, thereby promoting cuproptosis. The calcium ions enriched in the MAMs structure play an important regulatory role in this process, further revealing the mechanistic role of MAMs in DATs‐regulated RAB18 functionality.

Additionally, based on the current results, we can conclude that RAB18 promotes the increase in mitochondrial numbers through the MAMs structure. Further proteomic analysis revealed that after RAB18 overexpression, significant changes occurred in the mitochondrial‐related metabolic pathways within the cells. KEGG analysis indicated that key metabolic pathways, such as oxidative phosphorylation and the TCA cycle, were more active, providing essential metabolic support for cellular cuproptosis (Figure S7b, Supporting Information). To further assess the impact of RAB18 on mitochondrial biosynthesis, we specifically focused on the biological process terms in the GO enrichment analysis related to mitochondria. The results showed that RAB18, by promoting mitochondrial biosynthesis, mitochondrial membrane organization, and cellular copper ion homeostasis, not only increased mitochondrial numbers but also provided the necessary metabolic environment for cuproptosis (Figure S7d, Supporting Information). These findings further highlight the central role of RAB18 in regulating cuproptosis and reveal how it enhances mitochondrial function to provide favorable metabolic support for cellular cuproptosis.

### 1.8 RAB18 Mediates Cuproptosis through Lipophagy Regulation to Promote Liver Fibrosis

**Figure 8:**
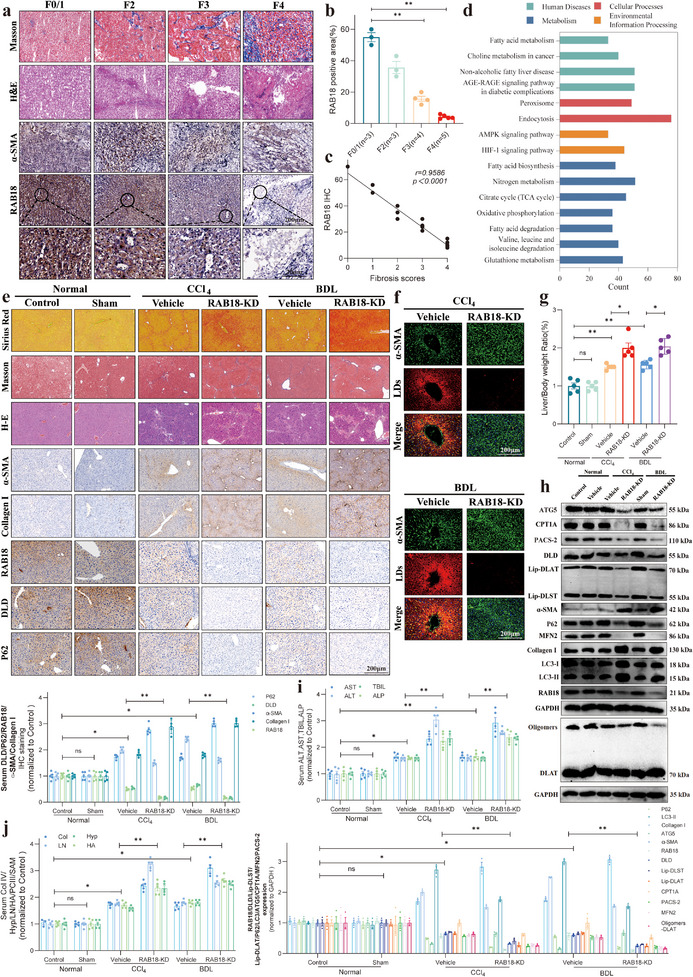
RAB18 mediates cuproptosis through lipophagy regulation to promote liver fibrosis. a) Liver fibrosis staging was performed using the Ishak score. For histopathological analysis, H&E, Masson, and IHC were used to stain a‐SMA and RAB18 in 15 human liver samples (F0/1, 3; F2, 3; F3, 4; F4, 5). Representative images are shown. Scale bar: 200 µm, 20 µm (boxes), *n* = 6/group. b) Quantification of positive RAB18 IHC staining (*n* = 6/group). **P < 0.01; *P < 0.001. c) Correlation analysis between RAB18 IHC and fibrosis scores in human liver fibrosis. Spearman's correlation coefficients (*r*) and *P* values are indicated. d) Transcriptomic analysis was performed to identify gene expression differences between normal mouse liver tissue and fibrotic liver tissue. GO analysis was carried out (*n* = 3). e) H&E staining, Masson, Sirius Red, and α‐SMA, Collagen I, RAB18, DLD, and P62 IHC analysis were performed on liver tissue from control and Sham groups, as well as CCl_4_/BDL‐induced mouse liver fibrosis models (Vehicle and RAB18‐KD groups, *n* = 5). Scale bar: 200 µm. f) Colocalization of α‐SMA (green) and LDs (red) in liver tissue from CCl_4_/BDL‐induced mouse liver fibrosis models (Vehicle and RAB18‐KD groups, *n* = 5) was analyzed using immunofluorescence (*n* = 3). Scale bar: 200 µm. g) Liver weight‐to‐body weight ratio from control and Sham groups, as well as CCl_4_/BDL‐induced mouse liver fibrosis models (Vehicle and RAB18‐KD groups, *n* = 5). h) Immunoblotting was performed to assess the expression levels of α‐SMA, Collagen I, RAB18, ATG5, P62, LC‐3, CPT1A, PACS‐2, MFN2, DLD, Lip‐DLST, Lip‐DLAT, and oligomerized DLAT proteins in liver tissue from normal mice (control and Sham groups) and CCl_4_/BDL‐induced liver fibrosis models (Vehicle and RAB18‐KD groups, *n* = 5), with quantification using grayscale analysis (*n* = 3). i) Serum analysis was conducted to assess the expression levels of Alanine Aminotransferase (ALT), Aspartate Aminotransferase (AST), Total Bilirubin (TBIL), and Alkaline Phosphatase (ALP) in serum collected from normal mice (control and Sham groups) and CCl_4_/BDL‐induced liver fibrosis models (Vehicle and RAB18‐KD groups, *n* = 5). j) Serum analysis was performed to evaluate the expression levels of Collagen (Col), Hydroxyproline (Hyp), Laminin (LN), and Hyaluronic Acid (HA) in serum from normal mice (control and Sham groups) and CCl_4_/BDL‐induced liver fibrosis models (Vehicle and RAB18‐KD groups, *n* = 5). Data are presented as mean ± SD, with *p*‐values calculated using one‐way ANOVA. ns, not significant; *P < 0.05, **P < 0.01.

To further explore the relationship between RAB18 and the pathological progression of liver fibrosis, we analyzed the expression of RAB18 in human liver fibrosis clinical samples. First, through H&E staining, Masson staining, and a‐SMA (HSCs activation marker) immunohistochemistry (IHC) analysis, we observed typical fibrotic features in liver samples from patients with liver fibrosis (**Figure** **8**a). Semi‐quantitative analysis of human liver samples based on the Metavir score showed a significant negative correlation between the expression of RAB18 and the patient's F stage (Figure 8b,c, *r* = 0.9586, *P* < 0.0001) (Figure 8b,c). Next, we investigated the expression of RAB18 in two commonly used experimental liver fibrosis mouse models, including the carbon tetrachloride (CCl_4_)‐induced liver fibrosis model and the bile duct ligation (BDL)‐induced liver fibrosis model. Histological analysis using H&E staining, Masson's trichrome staining, and immunohistochemistry (IHC) for α‐SMA and Collagen I revealed typical fibrotic features in the livers of mice (Figure 8e and Figure S7f, Supporting Information), and the liver weight/body weight ratio significantly increased in both model groups (Figure S8e, Supporting Information). In both liver fibrosis models, the progression of fibrosis was significantly aggravated after knocking down RAB18 expression in the liver using AAV8‐RAB18KD. IHC results indicated a significant negative correlation between the expression of RAB18 and a‐SMA. With the downregulation of RAB18 protein, the expression of DLD and P62 proteins also significantly decreased, indicating that RAB18 plays a key role in the regulation of liver fibrosis by modulating lipophagy and cuproptosis (Figure 8e). Additionally, to verify the liver‐targeting ability of the AAV8 vector and ensure efficient knockdown of RAB18 in liver tissues while avoiding systemic inflammatory responses to improve the stability and reproducibility of experimental results, we assessed RAB18 protein expression in the heart, liver, lungs, kidneys, muscles, and small intestine after AAV8 model construction. The results confirmed the liver‐specific knockdown of RAB18. RAB18 protein was significantly downregulated only in the liver (Figure S8a, Supporting Information).

To further explore the molecular mechanisms behind these pathological changes, we systematically compared the gene expression profiles of liver tissues from normal control mice and model mice based on transcriptomic data, with a particular focus on gene changes related to cuproptosis and lipophagy. The analysis revealed significant changes in the expression of several key genes in the model group, with notable differences in the expression of RAB18 and DLD, suggesting that these genes may play important regulatory roles in the onset and progression of liver fibrosis (Figure S8c, Supporting Information). To further analyze the biological functions of these differentially expressed genes and the signaling pathways they participate in, we performed GO functional enrichment analysis and KEGG signaling pathway analysis. The GO analysis results showed significant enrichment of several biological processes closely related to cuproptosis and lipophagy, indicating that these two mechanisms may play a central role in the pathology of liver fibrosis. The KEGG analysis results were highly consistent with the GO analysis, showing significant enrichment in multiple signaling pathways associated with cuproptosis and autophagy (Figure 8d and Figure S8b, Supporting Information). These findings provide important theoretical support for further exploring the specific mechanisms by which RAB18 and DLD regulate cuproptosis and lipophagy in HSCs.

To further investigate the important role of RAB18 in HSCs, we performed immunofluorescence colocalization analysis using α‐SMA to label HSCs and Nile Red dye to mark lipid droplets. The results showed that in both liver fibrosis models, after knockdown of RAB18, the colocalization of lipid droplets with α‐SMA significantly decreased (Figure 8f and Figure S8g,h, Supporting Information). Meanwhile, in these two models, after RAB18 knockdown, the liver weight/body weight ratio significantly increased (Figure 8g), suggesting that RAB18 knockdown exacerbated liver enlargement. Further analysis revealed that after RAB18 knockdown, the expression of liver fibrosis markers α‐SMA and Collagen I increased, and the expression of ATG5 and LC3‐II also significantly rose. Additionally, the expression of P62, DLD, PACS‐2, MFN2, lipoic acid‐modified proteins, and DLAT oligomerization proteins significantly decreased, indicating that RAB18 knockdown activated autophagy and inhibited cuproptosis, while also disrupting MAMs structures (Figure 8h). The detection of liver injury‐related enzymes (ALT, AST, and ALP) and liver fibrosis markers (IV‐C, PC‐III, HA, SMA, Hyp, and LN) also showed that RAB18 knockdown significantly promoted the progression of liver fibrosis (Figure 8I,j). In summary, these results suggest that RAB18 plays a critical role in the progression of liver fibrosis by regulating lipophagy and cuproptosis. Targeting RAB18 and inhibiting lipophagy to induce cuproptosis can effectively slow down the pathological progression of liver fibrosis.

### 1.9 DATs Target RAB18 to Inhibit Lipophagy and Induce Cuproptosis in HSCs In Vivo

**Figure 9:**
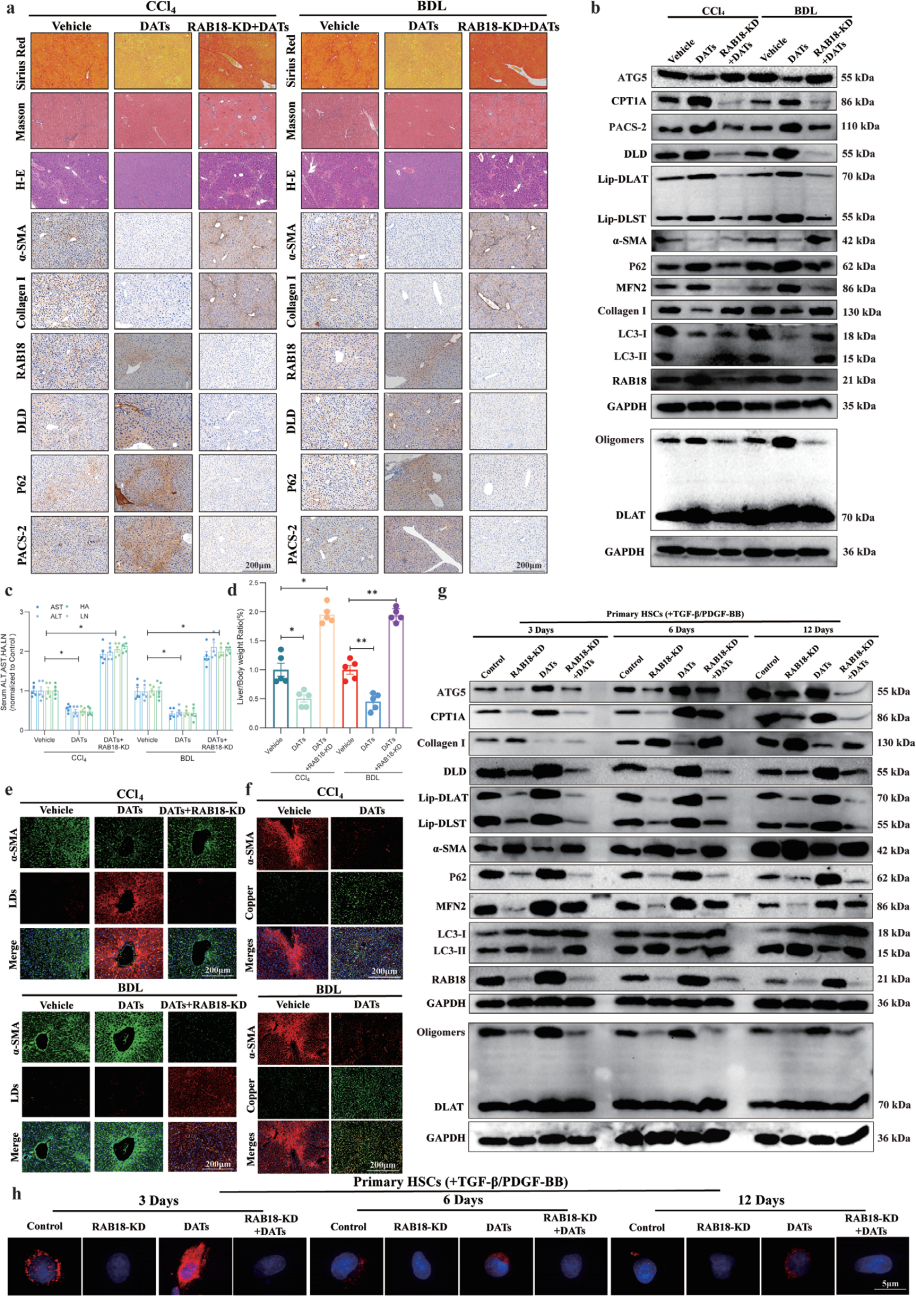
DATs target RAB18 to inhibit lipophagy and induce cuproptosis in HSCs in vivo. a) H&E staining, Masson, Sirius Red, and α‐SMA, Collagen I, RAB18, DLD, PACS‐2, and P62 IHC analysis were performed on liver tissue collected from CCl_4_/BDL‐induced mouse liver fibrosis models treated with Vehicle, DATs, or RAB18‐KD + DATs (*n* = 5). Scale bar: 200 µm. b) Immunoblotting was used to evaluate the expression levels of α‐SMA, Collagen I, RAB18, ATG5, P62, LC‐3, CPT1A, PACS‐2, MFN2, DLD, Lip‐DLST, Lip‐DLAT, and oligomerized DLAT proteins in liver tissue from CCl_4_/BDL‐induced mouse liver fibrosis models treated with Vehicle, DATs, or RAB18‐KD + DATs (*n* = 5), with quantification using grayscale analysis (*n* = 3). c) Serum analysis was conducted to assess the expression levels of AST, ALT, LN, and HA in serum from CCl_4_/BDL‐induced mouse liver fibrosis models treated with Vehicle, DATs, or RAB18‐KD + DATs (*n* = 5). d) The liver weight‐to‐body weight ratio was calculated for liver tissue collected from CCl_4_/BDL‐induced mouse liver fibrosis models treated with Vehicle, DATs, or RAB18‐KD + DATs (*n* = 5). e) Colocalization of α‐SMA (green) and LDs (red) in liver tissue from CCl_4_/BDL‐induced mouse liver fibrosis models treated with Vehicle, DATs, or RAB18‐KD + DATs was analyzed by immunofluorescence (*n* = 5). Scale bar: 200 µm. f) Colocalization of α‐SMA (red) and copper (green) in liver tissue from CCl_4_/BDL‐induced mouse liver fibrosis models treated with vehicle and DATs was analyzed by immunofluorescence (*n* = 5). Scale bar: 200 µm. g) Immunoblotting was performed to assess the expression levels of α‐SMA, Collagen I, RAB18, ATG5, P62, LC‐3, CPT1A, PACS‐2, MFN2, DLD, Lip‐DLST, Lip‐DLAT, and oligomerized DLAT proteins in primary HSCs isolated from control and RAB18‐KD mouse livers and treated with DATs (10 µm) (*n* = 3). h) Fluorescence images were obtained to assess the number of lipid droplets in primary HSCs isolated from control and RAB18‐KD mouse livers and treated with DATs (10 µm) (*n* = 3). Scale bar: 5 µm. Data are presented as mean ± SD, with *p*‐values calculated using one‐way ANOVA. ns, not significant; *P < 0.05, **P < 0.01.

In vitro experiments showed that DATs significantly inhibited HSCs activity by targeting RAB18. To further verify its antifibrotic effects and potential mechanisms in vivo, we constructed two common experimental liver fibrosis models, including the CCl_4_‐induced liver fibrosis model and the BDL‐induced liver fibrosis model. We used the AAV8 vector to specifically knock down RAB18 expression in the liver and performed oral gavage treatment experiments. After DATs treatment, the expression levels of RAB18 were significantly increased, while the expression levels of α‐SMA and Collagen I were correspondingly decreased. H&E staining results showed that DATs treatment significantly improved liver tissue damage in the model group mice. Masson staining, Sirius Red staining, and Hematoxylin staining further indicated that DATs treatment reduced collagen deposition around the central vein(**Figures** **9**a and S7f,I, Supporting Information). Moreover, DATs treatment significantly decreased the expression of α‐SMA, Collagen I, ATG5, and LC3‐II in the liver. At the same time, the expression of P62, DLD, PACS‐2, MFN2, lipoic acid‐modified proteins, and DLAT oligomerized proteins significantly increased, suggesting that DATs exert therapeutic effects on liver fibrosis by inhibiting lipophagy and inducing cuproptosis (Figure 9b and Figure S8f, Supporting Information). Further analysis showed that DATs treatment significantly reduced the expression of liver injury‐related enzymes ALT and AST, as well as liver fibrosis markers HA and LN (Figure 9c). After DATs treatment, the liver weight/body weight ratio in the model mice significantly decreased (Figure 9d), further confirming the antifibrotic effect of DATs.

Additionally, previous studies have shown that DATs exhibit no significant toxicity to human normal liver cells (Thle2). To further assess the potential toxicity of DATs to the liver, we performed oral gavage treatment experiments in normal mice. The results showed no significant differences in H&E staining, Masson staining, Sirius Red staining, and Hematoxylin staining between the DATs treatment group and the control group in normal mice (Figure S9a, Supporting Information). Measurements of liver weight/body weight ratio, liver injury‐related enzymes ALT, AST, and liver fibrosis markers HA and LN also indicated that DATs did not cause significant toxicity in normal mice (Figure S9b,c, Supporting Information), confirming its good safety profile.

In the CCl_4_‐induced liver fibrosis model and the BDL model pretreated with AAV8‐RAB18KD, the antifibrotic effect of DATs was significantly weakened. Specifically, H&E staining results showed increased tissue structural damage, while Masson staining, Sirius Red staining, and Hematoxylin staining results indicated a marked increase in collagen deposition around the central vein. Additionally, the expression of α‐SMA and Collagen I was significantly higher in the AAV8‐RAB18KD pretreatment group than in the DATs‐only treatment group (Figure 9a), suggesting that RAB18 knockdown inhibited the antifibrotic effect of DATs. Western blot results showed that after AAV8‐RAB18KD pretreatment, the therapeutic effect of DATs was significantly reversed. The expression of P62, CPT1A, DLD, lipoic acid‐modified proteins, and oligomerized DLAT proteins significantly decreased, while the expression of autophagy‐related proteins ATG5 and LC3‐II significantly increased (Figure 9b and Figure S8f, Supporting Information). To further assess liver enlargement, we measured the liver weight/body weight ratio. The results indicated that after AAV8‐RAB18KD pretreatment, the liver weight/body weight ratio of mice treated with DATs significantly increased, and the expression of liver injury‐related enzymes ALT and AST, as well as liver fibrosis markers HA and LN, significantly increased (Figure 9c,d). These results further confirm that RAB18 plays a critical role in the antifibrotic effect mediated by DATs, with DATs regulating RAB18 to inhibit lipophagy and induce cuproptosis.

In the CCl_4_‐induced liver fibrosis model, we used GYY4137 as a positive control and set two dosing concentrations, low (15 mg kg^−1^) and high (30 mg kg^−1^), combined with AAV8‐RAB18KD treatment in mice to systematically explore the central role of RAB18 in DATs‐mediated liver fibrosis treatment. The results further validated the important role of RAB18 in this process, consistent with previous conclusions (Figure S9d,e, Supporting Information).

To verify whether DATs inhibit lipophagy and induce cuproptosis by targeting HSCs, we performed immunofluorescence colocalization analysis. The results showed that in the liver tissues of mice from the two liver fibrosis model groups treated with DATs, the colocalization levels of copper ions with lipid droplets (LDs) and α‐SMA were significantly increased (Figure 9e,f and Figure S9f–i, Supporting Information). However, after AAV8‐RAB18KD pretreatment, this effect was significantly reversed, with a notable decrease in the colocalization of LDs and α‐SMA (Figure 9e,f and Figure S9f–I, Supporting Information). At the same time, after DATs treatment, the fluorescence intensity of α‐SMA in the model group significantly decreased. These results further support the hypothesis that DATs regulate RAB18 to inhibit lipophagy and induce cuproptosis in HSCs.

Subsequently, to further explore the specificity and targeting of DATs for HSCs and RAB18, we extracted primary HSCs and cultured them in vitro, adding HSCs activation agents TGF‐β and PDGF‐BB. We then measured the changes in lipophagy and cuproptosis‐related markers at 3, 6, and 12 d. The results showed that as HSCs were activated from their resting state, autophagy levels gradually increased and the number of lipid droplets gradually decreased, consistent with our previous research findings. Additionally, RAB18 expression also gradually declined, indicating that RAB18 expression negatively correlates with the pathological progression, which aligns with our observations in clinical samples. Unexpectedly, we found that during HSCs activation, the level of cuproptosis was suppressed, a finding not previously reported, which may be related to the gradual decline in RAB18 expression. During HSCs activation, DATs upregulated RAB18 expression and increased lipid droplet numbers, significantly reducing the expression of α‐SMA, Collagen I, ATG5, and LC3‐II. Additionally, DATs upregulated the expression of CPT1A, P62, DLD, PACS‐2, MFN2, lipoic acid‐modified proteins, and DLAT oligomerization proteins, thus inhibiting lipophagy and inducing HSCs cuproptosis (Figure 9g,h and Figure S8j, Supporting Information). This effect was significantly reversed by AAV8‐RAB18KD pretreatment at different stages of HSCs activation.

In conclusion, these results suggest that DATs, in the CCl_4_‐induced liver fibrosis mouse model, inhibit lipophagy in HSCs, leading to lipid droplet accumulation and effectively inducing cuproptosis. This process relies on the regulatory role of RAB18, further validating the targeting and specificity of DATs, and highlighting their potential value in antifibrotic therapy.

**Figure 10:**
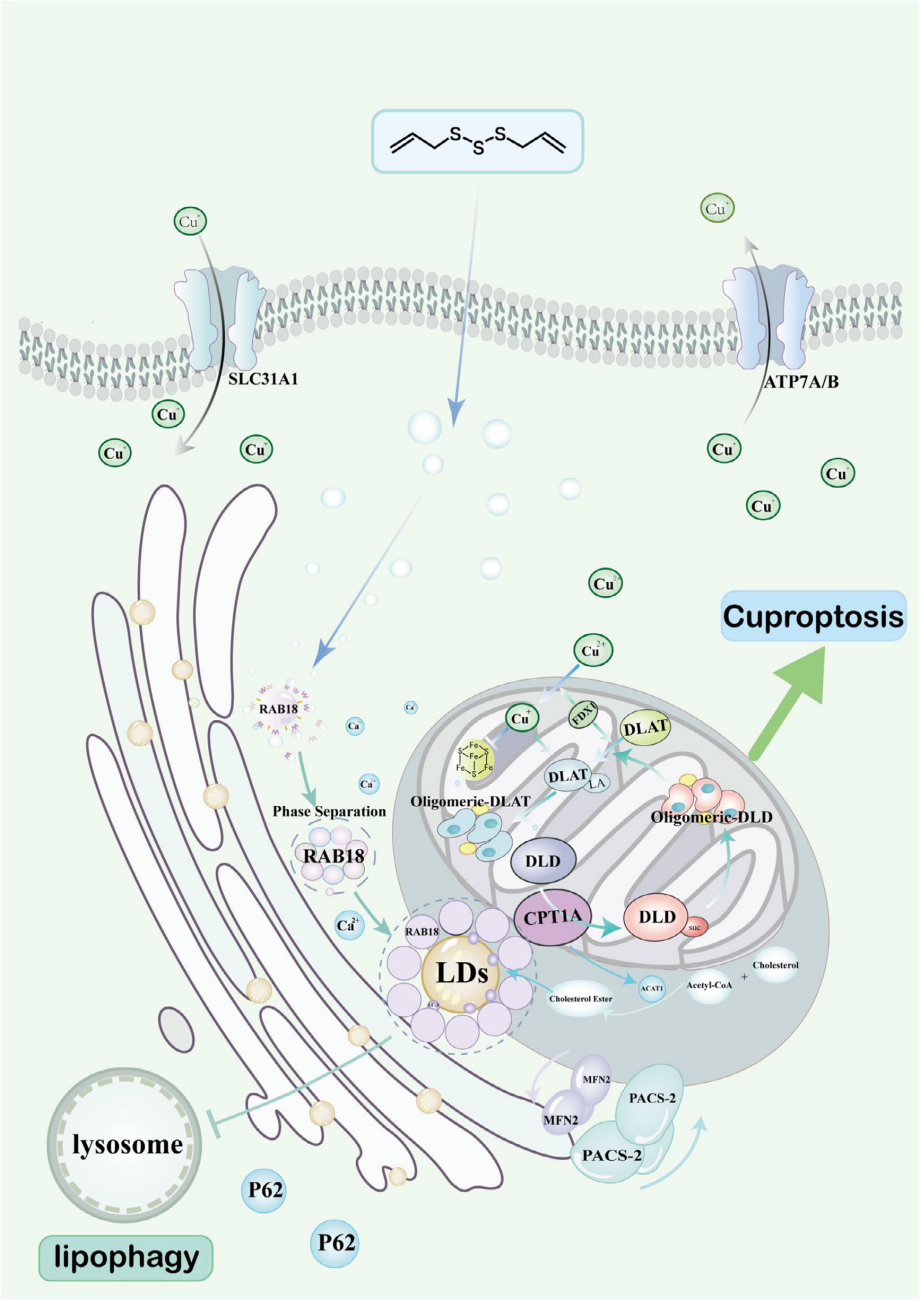
Mechanism Diagram.

This study not only unveils a novel pathway by which DATs induce HSCs cuproptosis through multiple mechanisms in vivo and in vitro to exert antifibrotic effects, but also for the first time confirms that RAB18 LLPS is closely associated with MAMs formation. We provide a detailed analysis of its key role in inhibiting lipophagy and inducing cellular cuproptosis. These findings offer a new perspective on understanding the pathogenesis of liver fibrosis and provide a theoretical basis for developing antifibrotic therapies targeting RAB18 and cuproptosis. This not only expands the application potential of DATs in antifibrotic drugs but also explores a new direction for the development of active ingredients from traditional Chinese medicine (**Figure** **10**).

DATs from garlic extract regulate the LLPS of RAB18, where the phase‐separated RAB18 encapsulates lipid droplets to inhibit lipophagy and recruit CPT1A, promoting the formation of mitochondrial‐associated membrane structures (MAMS). As the formation of MAMS increases, the calcium ion concentration in that region rises, further facilitating the LLPS of RAB18 and creating an amplifying positive feedback loop. Meanwhile, the recruited CPT1A enhances the succinylation of DLD, thereby increasing its enzymatic activity and enhancing the lipoic acid modification of the protein, which induces copper toxicity effects in HSCs for antifibrotic effects.

## 2 Discussion

In traditional Chinese medicine, garlic, as a warming and aromatic herb, is classified as a food‐medicine dual‐purpose herb with functions of expelling wind, activating blood circulation, and promoting qi flow. It is warm in nature and pungent in flavor, entering the liver meridian, where it can warm and unblock the liver channels, promote liver qi circulation, and facilitate the flow of qi and blood. Garlic's ability to activate blood and promote qi flow helps relieve pathological conditions such as liver qi stagnation and blood stasis, which are common in liver fibrosis patients. It is also effective in alleviating symptoms like liver area distension and qi stagnation. Its detoxifying, antiparasitic, and damp‐heat clearing effects support liver detoxification, reducing inflammation caused by toxin accumulation, thereby inhibiting the progression of fibrosis.^[^ ^7^ ^]^

However, garlic's main active compound, allicin, has unstable chemical properties and easily generates a series of reactive sulfur‐containing compounds, such as DADs and DATs. These sulfur‐containing compounds, due to the chemical nature of their sulfur atoms, produce highly reactive oxidative intermediates such as sulfoxides, sulfones, and free radicals during metabolism in the body.^[^ ^37^ ^]^ These oxidative products can react with the thiol group of GSH, significantly depleting intracellular GSH reserves. The sulfur radicals (R−S·) generated during the sulfur ether metabolism process have high electrophilicity and can combine with GSH to form disulfides, further depleting GSH and oxidizing it into glutathione disulfide, thereby lowering the active level of GSH.^[^ ^38^ ^]^ Due to the relatively large atomic radius and low electronegativity of sulfur atoms, sulfur ether metabolites frequently react with GSH, significantly lowering intracellular GSH levels, and may lead to the accumulation of metal ions, particularly copper ions.^[^ ^14^ ^]^

Copper, an essential trace element, plays a critical role in metabolic regulation and maintaining normal physiological functions in the liver.^[^ ^39^ ^]^ However, when copper metabolism becomes dysregulated and leads to elevated copper levels in the body, it can cause significant toxicity to the liver and accelerate the onset of pathological fibrosis.^[^ ^40^ ^]^ Based on analysis from the GEO database, we found that in the liver tissue of patients with liver fibrosis, the GSH synthesis pathway and TCA cycle exhibited significant abnormalities, and genes associated with cuproptosis showed marked expression changes. During the progression of liver fibrosis, activated HSCs, under damage or inflammatory signals, transform into myofibroblast‐like cells that have proliferative capacity and secrete extracellular matrix, leading to excessive collagen deposition and accumulation of the extracellular matrix, which are hallmark features of liver fibrosis.

It is noteworthy that the concentration of copper ions in fibrotic liver tissue is significantly higher than that in normal liver tissue, and this high copper state provides favorable conditions for the copper‐induced cell death mechanism in HSCs. While excess copper in the liver is toxic, with proper regulation, the copper death mechanism can be exploited to selectively induce HSCs death, thereby modulating the progression of fibrosis. Therefore, copper death, as an emerging form of cell death, offers a potential targeted strategy for the treatment of liver fibrosis.

In this study, we targeted HSCs with DATs to inhibit their activation and induce cuproptosis, aiming to explore its potential in treating liver fibrosis. Our results indicate that after DATs administration, the expression of copper death markers FDX1 and LIAS showed a significant time‐dependent change: within the first 24 h, the expression of FDX1 and LIAS gradually increased, while it significantly decreased after 48 h. This phenomenon suggests that DATs may initially activate the cells’ adaptive response, followed by the initiation of a negative feedback regulation mechanism to modulate copper metabolism in HSCs, ultimately exerting therapeutic effects.^[^ ^41^ ^]^

In the early stages of treatment, the upregulation of FDX1 and LIAS expression may be an adaptive response of HSCs to DATs stimulation. As copper‐dependent enzymes, FDX1 and LIAS play key roles in copper transport and the maintenance of mitochondrial function. The copper accumulation induced by DATs might enhance the expression of these enzymes, promoting copper activation and the generation of reactive oxygen species, thereby inducing copper death. The increase in FDX1 and LIAS helps enhance the cell's dependence on copper‐related metabolic activities and aids the cell in coping with oxidative stress in the short term. As DATs administration continues for 48 h, the expression of FDX1 and LIAS gradually decreases, suggesting that HSCs may initiate a negative feedback regulation mechanism to cope with the potential toxicity caused by excessive copper. Prolonged copper accumulation can trigger oxidative stress, damaging cellular lipids, proteins, and DNA, leading to impaired cellular functions and even cell death. To mitigate copper toxicity, HSCs reduce the expression of copper‐dependent enzymes, thereby decreasing copper transport and utilization, thus preventing further accumulation of copper. This regulatory mechanism helps the cell reduce the excessive accumulation of copper ions and prevent further damage from copper toxicity. However, when copper accumulation and oxidative stress damage reach a certain threshold, HSCs may be unable to repair the damage and initiate programmed cell death mechanisms such as apoptosis. In this process, the downregulation of copper‐related enzymes is essentially a self‐protection strategy to reduce copper‐dependent metabolic activity. While this regulation helps alleviate copper toxicity, it may ultimately provide conditions for cell death. In other words, the cell uses this self‐protection mechanism to reduce the negative impact of copper, and in the end, may enter the death process to prevent further damage. The dynamic changes in FDX1 and LIAS reflect the adaptive response and regulatory mechanisms of HSCs when faced with DATs treatment, providing new insights into the application of copper death in the treatment of liver fibrosis.

This study reveals the relationship between elevated copper ions and HSCs‐specific copper death, providing new evidence for the dual role of copper metabolism in liver fibrosis and laying the theoretical foundation for antifibrotic therapy based on copper death mechanisms. Additionally, our research suggests that DATs have potential therapeutic applications in copper death‐related treatments. We found that DATs significantly upregulated the expression of DLD. DLD plays a critical role in maintaining the level and stability of lipoic acid modification by catalyzing the oxidation of dihydrolipoyl to active lipoic acid, allowing the regeneration and re‐entry of lipoic acid into metabolic pathways. By regulating the oxidation and regeneration of lipoic acid, DLD is essential for sustaining the normal process of lipoic acid modification. When copper ion levels are excessively high, these ions bind with lipoic acid‐modified proteins in the mitochondria, leading to protein misfolding and aggregation. The catalytic activity of DLD, by maintaining the functional state of lipoic acid, ensures the proper progression of lipoic acid modification. Therefore, in copper‐induced cell death, DLD becomes a key regulatory factor in copper‐induced cellular damage and death signals through the modulation of lipoic acid levels and the lipoic acid cycle.

TCA cycle abnormalities are a hallmark of copper‐induced cell death, leading to the production of large amounts of succinyl‐CoA. Succinyl‐CoA not only serves as an energy source but also participates in various pathological processes through the regulation of protein succinylation. Studies have shown that under oxidative stress, succinylation of specific sites on glutaminase (GLS), such as K311 and H475, promotes its oligomerization.^[^ ^22^ ^]^ This modification enhances the intermolecular interactions and structural stability of GLS, while also increasing its enzymatic activity, thus optimizing the cell's response to oxidative stress. Additionally, succinylation modifies specific lysine residues on the influenza virus nucleoprotein, promoting its oligomerization and increasing its retention in the nucleus.^[^ ^20^ ^]^ This process interferes with the viral replication cycle, significantly inhibiting influenza virus infectivity. In this study, DATs significantly enhance the expression of CPT1A, promote the succinylation‐ubiquitination site competition at the K320 site of DLD, increase its succinylation, and inhibit ubiquitin‐mediated degradation. Succinylated DLD not only undergoes oligomerization but also enhances its catalytic function, further increasing its succinylation level by providing more lipoic acid. Under these conditions, the oligomerization of DLAT is enhanced, inducing cuproptosis in LX2 cells. These results show that DLD enhances its enzymatic activity by competing between succinylation and ubiquitination sites, boosts lipoic acid modification, and promotes copper death. In conclusion, DLD plays a core role in maintaining the stability of lipoic acid modification and, by providing lipoic acid, drives copper metabolism imbalance and cell death. Proteomics studies reveal that DATs not only induce copper death but also affect cell activity through the regulation of autophagy and lipid synthesis. Further proteomic analysis highlights RAB18's prominent role in interacting with CPT1A, with these proteins being highly related to lipid metabolism and autophagy. We found that inhibiting lipophagy can effectively induce copper death, with RAB18 being a key regulatory target in this process. The Cu/RAB18/CPT1A/DLD axis has been established as the core regulatory pathway in copper metabolism, revealing the mechanistic link between lipophagy and copper‐induced cell death.

Our study also indicates that DATs induce LLPS of RAB18 by binding to its K153 site and simultaneously recruit CPT1A, mediating the formation of MAMs. The formation of MAMs accelerates RAB18 phase separation, potentially due to the high concentration of calcium ions within MAMs. Phase‐separated RAB18 further inhibits lipophagy and promotes cuproptosis. Overall, our study reveals a positive feedback loop: DATs induce RAB18 phase separation, enhance the coupling between the endoplasmic reticulum and mitochondria, promote MAMs formation, and MAMs, in turn, accelerate RAB18 phase separation. Phase‐separated RAB18 recruits CPT1A, enhances the succinylation modification of DLD, and increases its enzymatic activity, thereby inducing copper death in a high copper environment. This process not only deepens our understanding of the regulatory mechanisms of lipophagy and copper death in liver fibrosis but also highlights RAB18 as a key bridge between lipophagy and copper death, playing a crucial role in HSCs.

In conclusion, DATs target RAB18 and induce its phase separation, becoming a key regulatory factor in inhibiting lipophagy and inducing copper death. These results highlight the important role of RAB18 in the pathological process of liver fibrosis and provide a potential target for evaluating the therapeutic effects of DATs. Given that copper accumulation in fibrotic liver tissue is higher than in normal tissue, this strategy offers a new direction for research into copper death mechanisms and presents significant clinical therapeutic prospects for liver fibrosis.

## 3 Experimental Section

### Patient specimens

This study collected liver tissue samples from patients treated at Nanjing Hospital of Traditional Chinese Medicine, Nanjing University of Traditional Chinese Medicine, between February 2013 and June 2017. All patients signed informed consent forms, and the study protocol was approved by the Medical Ethics Committee of Nanjing Hospital of Traditional Chinese Medicine, Nanjing University of Traditional Chinese Medicine. The study adhered to the principles of the Declaration of Helsinki. Histological examinations, including α‐SMA staining and RAB18 staining, were conducted using paraffin sections. Based on the Metavir scoring system, the histological stages of fibrosis were classified as follows: no fibrosis (F0/1, *n* = 3), mild fibrosis (F2, *n* = 3), and severe fibrosis (F3/F4, *n* = 9). Detailed patient characteristics can be found in the previous study.^[^ ^42^ ^]^

### Animals and Grouping

The animal experiments were conducted at the Animal Experiment Center of Nanjing University of Chinese Medicine and were approved by the Ethics Committee of Nanjing University of Chinese Medicine (Approval No.202405A053). The experiment used 8‐week‐old male SPF‐grade Institute of Cancer Research (ICR) mice, weighing ≈20 g, purchased from Hangzhou Medical College. The mice were housed in ultraclean air flow racks with free access to food and water. The AAV8‐ssAAV.TBG669.GFAP.mRab18_miR30 (1E+13 GC mL^−1^) viral vector, targeting liver mRab18 gene knockdown, was provided by PackGene Biotech and administered via tail vein injection in mice with BDL and intraperitoneal injection of carbon tetrachloride (CCl₄). Liver fibrosis models were constructed using two methods: BDL and intraperitoneal injection of CCl₄. For the BDL model, mice were first anesthetized with 3% pentobarbital sodium (40 mg kg^−1^) by intraperitoneal injection, fixed in a supine position on a surgical table, and the surgical area was disinfected. The abdominal cavity was opened along the midline, and the bile duct near the duodenum was isolated. Two 7‐0 sutures were used to ligate the bile duct below and the bile duct was cut in between the ligations. The inner layer was sutured with 7‐0 stitches and the outer layer with 5‐0 stitches. After surgery, the mice were placed on a heating pad to maintain an anal temperature of 37 ± 0.5 °C. The control group did not undergo bile duct ligation, with the remaining procedures the same. Liver fibrosis formation was successfully induced 14 d after surgery. For the CCl₄ model, mice were intraperitoneally injected with 10% CCl₄ (Sigma, St. Louis, Mo, USA) at a dose of 0.5 µL g−1, three times a week for 8 weeks to establish the liver fibrosis model. In this experiment, DATs were dissolved in a mixed solution consisting of 50% vegetable oil, 45% polyethylene glycol 300 (PEG300), and 5% Tween 80 (Tween 80). DATs were first dissolved in PEG300, then vegetable oil was added gradually and mixed thoroughly. Tween 80 was added as an emulsifier to form a stable emulsion. Finally, the solution was mixed thoroughly through ultrasonic treatment or with a high‐speed stirrer to ensure uniform distribution. After 1 week of adaptation feeding, the experimental mice were randomly divided into two main model groups. The first group consisted of normal control, normal control + empty plasmid, and normal control + DATs (high dose) groups. The second group consisted of liver fibrosis model groups, with the BDL model group further subdivided into Sham, BDL + DATs (high dose), BDL + positive drug, BDL + RAB18 shRNA, and BDL + RAB18 shRNA + DATs (high dose) groups. The CCl₄‐induced liver fibrosis model group was further subdivided into CCl₄ + empty plasmid, CCl₄ + DATs (low dose), CCl₄ + DATs (high dose), CCl₄ + positive drug, CCl₄ + RAB18 shRNA, CCl₄ + DATs (high dose) + empty plasmid, and CCl₄ + DATs (high dose) + RAB18 shRNA groups. After the liver fibrosis model was established, mice were administered low‐dose DATs (15 mg kg^−1^), high‐dose DATs (30 mg kg^−1^), or the positive drug GYY4137 (50 mg kg^−1^) by gavage daily for four weeks, with CCl₄ administered every other day during this period. Five ICR mice were used per group. At the end of the experiment, mice were euthanized following animal ethical guidelines, and tissues were collected for further analysis.

### Culture Conditions for Primary Cells and Cell Lines

LX2 and 293T cells (Chinese Academy of Sciences Shanghai Cell Bank, Shanghai) were cultured in incomplete Dulbecco’s Modified Eagle Medium (DMEM), while Thle‐2 cells were cultured in incomplete DMEM‐F12 medium, with 10% fetal bovine serum (FBS) and 1% penicillin‐streptomycin added. Cell line identification was performed for Thle‐2, LX2, and 293T cells. FBS was purchased from Kirgen Bioscience (Shanghai) Co., Ltd.; DMEM was purchased from NEST Biotechnology.

Primary mouse HSCs were isolated from mouse liver using a retrograde stepwise perfusion method. Initially, the liver was treated with a digestion solution containing streptomycin (200 U mL^−1^) and collagenase (0.2%). After digestion, cells were centrifuged to remove the majority of hepatocytes, and washed with Gey's balanced salt solution B (GBSS/B). The centrifugation was set at 100 × *g* for 5–10 min to eliminate most hepatocytes. Following this, the nonparenchymal cell pellet was further centrifuged at 1500 × *g* for 15 min to separate the cells. Next, a GBSS/B solution containing 10% Nycodenz was added, and the mixture was centrifuged at 1500 × *g* for another 15 min, ultimately isolating the primary HSCs. The isolated cells were then cultured in DMEM medium containing 10% FBS. All cell lines were maintained in a humidified incubator at 37 °C with 5% CO_2_. Cells were treated with different concentrations of DATs or other drugs for in vitro studies. Cell culture dishes and others were purchased from Hangzhou Agen biotechnology limited.

### CCK8 Cell Viability Assay

Cells were seeded in 96‐well plates at a density of 3000 cells per well, and after 24 h of drug intervention, cell viability and proliferation were assessed using a CCK8 assay kit. 10 µL of CCK8 reagent was added to each well, and the 96‐well plate was incubated at 37 °C with 5% CO₂ for 1 h. After incubation, absorbance was measured at 450 nm using a microplate reader, and cell viability at each concentration was calculated. The CCK8 assay (ZYCD002‐0100) kit was purchased from ZUNYAN, NanJing, China.

### Copper Ion Detection

After 24 h of drug intervention in LX2 cells, the culture medium was discarded, and cells were washed twice with Phosphate‐Buffered Saline (PBS) at room temperature. Cell pellets were collected by centrifugation. After cell lysis, the supernatant was used for copper ion content measurement, employing a copper ion detection kit (E‐BC‐K775‐M, Elabscience), strictly following the manufacturer's instructions for experimental operation and data collection.

### GSH Content Measurement

Following 24 h of drug treatment in LX2 cells, the supernatant was discarded and cells were washed twice with PBS at room temperature. Cell pellets were collected by centrifugation. After cell lysis, the supernatant was used to measure intracellular reduced GSH levels. GSH levels were determined using a commercial GSH assay kit (BB‐4713, Bestbio), with all procedures strictly adhering to the manufacturer's instructions.

### RT‐qPCR

Total RNA was extracted from LX2 cells and tissues using Trizol reagent, and RNA concentration was measured using a NanoDrop 2000 spectrophotometer. The extracted RNA was reverse‐transcribed and the resulting complementary DNA (cDNA) was diluted tenfold. Quantitative Polymerase Chain Reaction (qPCR) was performed on a QuantStudio 6 Flex system (Thermo Fisher Scientific, USA), using ABScript III RT Master Mix for qPCR with genomic DNA (gDNA) Remover (Accurata Biotechnology, ChangSha, China). The qPCR conditions were as follows: 95 °C predenaturation for 3 min, followed by 40 cycles of 95 °C denaturation for 5 s and 60 °C annealing/extension for 30 s. The 2^‐ΔΔCT method was used to analyze the qPCR results. Primer sequences used were: DLD (GAATGGCTGGTGGTGCTGTG; CCCTCTTCTTTCAACTGCTCTTCG).

### Western Blot

After 24 h of drug intervention, the supernatant was discarded and the cells were washed twice with PBS at room temperature. Cell lysis buffer (containing Phenylmethylsulfonyl Fluoride (PMSF), Phosphatase Inhibitor Solution (PS), etc.) was added and the cells were lysed on ice for 30 min to extract total proteins. The total protein concentration was determined using the BCA assay. Protein samples were denatured by adding sample loading buffer (5×SDS‐PAGE), followed by Sodium Dodecyl Sulfate–Polyacrylamide Gel Electrophoresis (SDS‐PAGE) electrophoresis (90 V for 20 min; 120 V for 120 min). Transfer was performed onto a 0.2 µm Polyvinylidene Fluoride (PVDF) membrane (110 V for 120 min). After the transfer, blocking buffer was used for 2 h to block nonspecific binding. The membrane was then cut according to the molecular weight of the target protein and incubated with the corresponding primary antibody. After the primary antibody incubation, a rabbit or mouse secondary antibody (1:10000) was added and incubated at room temperature for 2 h. Protein expression was detected using Enhanced Chemiluminescence (ECL) chemiluminescence, and protein expression was analyzed using a chemiluminescent gel imaging system. The antibodies used included FDX1 (HA721329) from HUABIO Group; HSP70(RM8223) from Biodragon; LIAS, DLD, CPT1A, SLC7A11, ATP7B, CPT1A, MFN2, PACS‐2, and RAB18 from Proteintech Group; Lipoic Acid from Abcom/Santa Cruz Biotechnology; ATP7A from Santa Cruz Biotechnology; GAPDH(P04406) from Promab Biotechnologies Inc. SuccinyllysinE from ptm‐bio lab. Enhanced Chemiluminescence (BMU102) was purchased from Abbkine; TBST was purchased from Swiss Affinibody LifeScience AG

### siRNA, Overexpression Plasmid, and Site‐Directed Mutant Plasmid Transfection

LX2 or 293T cells were seeded in six‐well plates. When LX2 cells reached 40–50% confluence or 293T cells reached 60%‐70%, SuperKine Lipo3.0 transfection reagent (Abbkine) or LNP30i Mate (LTR0301, BiOligo Biotechnology Shanghai) was mixed with Optimized Minimal Essential medium (Opti‐MEM) as per the manufacturer's instructions, and siRNA was diluted in Opti‐MEM. The diluted siRNA was combined with transfection reagent, incubated at room temperature for 10–15 min to form siRNA‐lipid complexes, and added to the cell culture. After 36 h, the culture medium was replaced, and after 72 h, cells were collected for further experiments. Sequence information:DLD(GCCCACTTATTCAAACAGAAT); CPT1A(CGTAGCCTTTGGTAAAGGAAT);MFN2(GCTCAGTGCTTCATCCCATTT);PACS‐2(GAACGATGACTTCGAGAAGAT);RAB18(GCACGAAAGCATTCCATGTTA);KAT2A(GCTGAACTTTGTGCAGTACAA);HAT1(CGGCGTGTTATTGAACGACTT).

### Transmission Electron Microscopy Characterization

TEM characterization was performed using a JEOL JEM‐2100 instrument operated at an accelerating voltage of 200 kV in bright‐field imaging mode. Samples were prepared as ultrathin sections ≈80 nm thick using an ultramicrotome and subsequently stained with uranyl acetate and lead citrate to enhance contrast. Images were acquired with a Gatan digital camera and processed using DigitalMicrograph software.

### Immunofluorescence and Probe Labeling

Cells were seeded at 2 × 10⁴ cells mL^−1^ on coverslips in 24‐well plates, treated as required, and fixed with paraformaldehyde, followed by membrane permeabilization with 0.5% Triton X‐100. According to experimental needs, appropriate primary antibodies were added and incubated overnight at 4 °C, followed by incubation with corresponding secondary antibodies (1:200) at room temperature for 2 h. Specific probes were used to label target molecules as needed. 4',6‐Diamidino‐2‐Phenylindole (DAPI) was applied to stain nuclei, and images were acquired under a fluorescence microscope at specific wavelengths to analyze the intracellular distribution and expression of target proteins or molecules.

### Co‐IP and MS Analysis

Cells were washed with prechilled PBS and lysed in IP buffer containing glycerophosphate, 1.5 mm magnesium chloride (MgCl₂), and 2 mm ethylene glycol tetraacetic acid. Lysates were centrifuged at 12 000 × *g* at 4 °C for 20 min to remove cell debris and unlysed cells. The collected supernatant was incubated with control IgG or target protein antibody in IP buffer containing protease inhibitors for 24 h. Subsequently, 30 µL of Protein G beads (Roche) was added to the mixture and incubated at 4 °C for 12 h to enrich the antibody‐bound protein complex. After incubation, beads were centrifuged at 2000 × *g* for 5 min at 4 °C, the supernatant was discarded, and beads were washed four times with IP buffer to remove nonspecific binding. Beads were then heated to 95 °C for 10 min to denature the proteins, followed by SDS‐PAGE for protein separation. Coomassie Brilliant Blue R‐250 (Bio‐Rad) was used for staining, and target protein bands were excised, decolorized, and digested with trypsin. The proteins were analyzed and identified on an Orbitrap Velos Pro mass spectrometer (Thermo Fisher Scientific) using liquid chromatography‐tandem mass spectrometry (LC‐MS/MS).

### RNA Sequencing and Cluster Analysis

Total RNA was extracted from mouse liver tissue and sequenced on the Illumina HiSeqTM 2000 platform. After RNA extraction, data were cleaned using Trimmomatic software to remove adapter sequences and low‐quality bases. The cleaned sequences were aligned to the mouse reference genome (e.g., Mus musculus GRCm38) using Burrows‐Wheeler Aligner (BWA) software. Gene expression levels were calculated as fragments per kilobase of transcript per million mapped reads, and DEGs were identified with a fold change ≥ 2 and adjusted *p*‐value < 0.05. DEGs were further subjected to GO functional enrichment analysis and KEGG pathway analysis to identify functional categories associated with biological processes, molecular functions, and cellular components, and to explore significantly enriched signaling pathways. To validate the RNA‐seq results, selected key pathway genes were analyzed by Quantitative Real‐time Polymerase Chain Reaction (qPCR) to confirm gene expression changes.

### Proteomics Analysis and Functional Annotation

Total protein was extracted from cell samples using Radio Immunoprecipitation Assay (RIPA) lysis buffer, and protein concentration was measured by BCA assay. Protein samples were separated by SDS‐PAGE, stained with Coomassie Brilliant Blue R‐250, and stained bands were excised, decolorized, reduced, alkylated, and digested with trypsin to generate peptides. Peptides were separated by high‐performance liquid chromatography and analyzed on an Orbitrap Velos Pro mass spectrometer (Thermo Fisher Scientific) using liquid chromatography‐tandem mass spectrometry (LC‐MS/MS). The data were processed and peptide identification was conducted using MaxQuant software, referencing species‐specific protein sequences in the UniProt database. Differentially expressed proteins were quantified by labeled quantification (such as Tandem Mass Tags (TMT) labeling) or label‐free quantification. Identified differentially expressed proteins were annotated by GO functional annotation and KEGG pathway enrichment analysis to reveal their involvement in biological processes, molecular functions, and cellular components, and to analyze significantly enriched signaling pathways.

### Fluorescence Recovery after Photobleaching (FRAP) Experiment

Fluorescence recovery after photobleaching (FRAP) experiments were performed using a laser scanning confocal microscope (Nikon A1 R HD 25) to study the diffusion behavior of Green Fluorescent Protein (GFP)‐fusion proteins. First, GFP‐fusion proteins were expressed in *E. coli* by genetic engineering, purified, and dissolved in 50 mm HEPES buffer (pH 7.4) and 150 mm NaCl to simulate physiological conditions. The prepared fluorescently labeled protein solution was placed in a glass‐bottomed culture dish and incubated at 37 °C to ensure stability during the experiment. In FRAP experiments, GFP‐fusion proteins were excited with a 488 nm laser, and a 1 s bleach was applied at 50% laser intensity. The recovery of fluorescence after bleaching was monitored in real‐time using time‐lapse imaging, and changes in fluorescence intensity were recorded at different time points. The confocal Petri dish (1050001) was sourced from SAINING Biotechnology.

### Protein Affinity Purification

In this study, recombinant plasmids carrying GFP‐fusion RAB18 protein or RAB18 protein with LYS‐153 site‐directed mutation were transformed into E. coli BL21 (DE3) strain to achieve efficient protein expression. The transformed E. coli was inoculated into Luria‐Bertani (LB) medium with antibiotics and cultured at 37 °C until an OD600 value of 0.6–0.8 was reached. Then, 0.5 mm isopropyl β‐D‐thiogalactopyranoside was added to induce the expression of GFP‐RAB18 or GFP‐RAB18 LYS‐153 mutant proteins, and the cells were further cultured overnight at 16 °C to ensure proper protein folding and retention of fluorescence and mutant protein functionality. After culture, the cells were collected and lysed by ultrasonication in lysis buffer containing 50 mm HEPES (pH 7.4), 150 mm NaCl, and 1 mm PMSF. After high‐speed centrifugation to remove insoluble debris, the supernatant was collected and purified by Ni‐affinity chromatography (Ni‐NTA column). After removing nonspecifically bound proteins with low‐concentration imidazole, the target protein was eluted from the column with elution buffer containing 250 mm imidazole. Purified protein concentration was monitored by fluorescence detection and purity was analyzed by SDS‐PAGE.

### Fluorescent Droplet Formation Assay

Fluorescent droplet formation assays were conducted using a laser scanning confocal microscope (Nikon A1 R HD 25) to investigate the liquid–liquid phase separation behavior of GFP‐fusion proteins. GFP‐fusion proteins were dissolved in 50 mm HEPES buffer (pH 7.4) and 150 mm NaCl to simulate physiological conditions. Protein solution was placed on a glass‐bottomed culture dish and incubated at 37 °C for 10–30 min to allow droplet formation. During the experiment, GFP‐fusion proteins were excited with a 488 nm laser, and the formation of fluorescent droplets was observed in real‐time using confocal microscopy.

### Atomic Force Microscopy (AFM) for Protein Liquid–Liquid Phase Separation and Drug Action Analysis

Purified proteins were dissolved in 50 mm HEPES buffer (pH 7.4) and 150 mm NaCl at a final concentration of 50 µm to simulate physiological intracellular conditions. A 10–20 µL protein solution was placed on a clean mica surface and left in a humid environment for 10–30 min to form protein droplets. The mica was then placed in an AFM liquid cell, and the same HEPES buffer was added to ensure complete immersion of the sample. Small molecule drugs were added during imaging to study their effect on protein liquid–liquid phase separation. The drug concentration was set at 10 µm. After adding the drug, AFM imaging in tapping mode was performed in liquid, with a scan range of 100 nm and scan speed of 0.5–1 Hz. Phase images were recorded, and differences in the mechanical properties of protein droplets before and after drug treatment were analyzed to evaluate viscoelastic changes within droplets and with surrounding solutions.

### Extraction of MAMS Structure

The extraction process for mitochondrial‐associated membranes (MAMs) involves two primary steps: isolation of crude mitochondrial particles and subsequent fine separation of MAMS, followed by protein quantification using the BCA method. Initially, an adequate quantity of cell samples or liver tissue was collected, minced, and washed, then centrifuged at 740 × *g*, 4 °C for 5 min to collect the supernatant. This supernatant underwent another 740 × *g* centrifugation for 5 min at 4 °C, followed by 9000 × *g* for 10 min, and 20 000 × *g* for 30 min, removing cell debris and obtaining crude mitochondrial particles. Lastly, the crude mitochondrial particles were pelleted at 100 000 × *g* for 1 h at 4 °C.

In the fine separation of MAMS, a Percoll density gradient solution was employed, and centrifugation at 95 000 × *g* at 4 °C for 30 min yielded five distinct layers, with the upper two containing MAMS. These layers were resuspended in Mitochondria Resuspension Buffer (MRB) solution and centrifuged again at 6300 × *g*, 4 °C, for 10 min for further purification. Subsequently, MAMS were pelleted again by centrifugation at 100 000 × g, 4 °C for 1 h, resuspended in MRB solution, and centrifuged at 6300 × g, 4 °C for 10 min to obtain purified MAMS. Protein quantification was conducted via the BCA method to ensure purity and accuracy for further analysis.

### HE Staining for Histological Analysis

Samples were fixed in a paraformaldehyde solution for 48 h, followed by dehydration in 70% ethanol for 12 h. The tissues were trimmed to suitable dimensions (0.5 cm × 0.5 cm × 0.5 cm), further dehydrated, and embedded in paraffin. Paraffin‐embedded samples were sectioned, baked, and deparaffinized, then sequentially stained with **hematoxylin and eosin** (H&E), air‐dried, and cover‐slipped. Finally, six different fields of view were randomly selected for observation and imaging under a microscope.

### Oil Red O Staining

Samples or cell sections were first fixed with 4% paraformaldehyde and pretreated with 60% isopropanol to optimize the staining environment. Samples were then immersed in a 0.5% Oil Red O staining solution for 10–15 min, allowing lipid droplets to stain red. Following staining, a mild destaining with 60% isopropanol was performed, and the samples were rinsed with PBS or distilled water. Hematoxylin was applied to stain nuclei, rendering them blue‐purple, and cover‐slipped for microscopic observation, where lipid droplets appeared red, and nuclei appeared blue‐purple.

### Immunohistochemistry (IHC)

Fresh tissue blocks were fixed in 4% paraformaldehyde, sequentially dehydrated in graded ethanol, cleared with xylene, and embedded in paraffin. After deparaffinization, antigen retrieval, and blocking of nonspecific sites, primary and secondary antibodies were sequentially added. DAB was used for chromogenic detection, and hematoxylin was used for nuclear counterstaining. Microscopy was used for imaging, followed by quantification in ImageJ. Threshold values were set to extract 3,3'‐Diaminobenzidine (DAB) signals, calculating the area proportion of positive cells. Hematoxylin staining identified total cell counts, ensuring comprehensive analysis across the entire tissue region. Measurements from multiple fields were averaged to provide representative quantitative data for statistical analysis.

### Coimmunoprecipitation (IP)

Following 24 h of drug treatment, cells were washed twice with PBS at room temperature, and lysed on ice with lysis buffer (containing PMSF, PS, etc.) for 30 min to extract total protein. After preclearing with Protein A/G agarose or magnetic beads, specific antibodies for target proteins were added to the lysate and incubated for antigen‐antibody binding. The antigen‐antibody complexes were captured with Protein A/G beads, followed by centrifugation or magnetic separation to collect the beads. After multiple washes to remove unbound proteins and nonspecific interactions, the complexes were denatured by boiling or eluted with buffer. Immunoprecipitated proteins were separated by SDS‐PAGE and identified by Western blot analysis.

### CETSA

CETSA is an experimental method used to evaluate the binding efficiency of drugs to intracellular target proteins. The principle of this assay is that target proteins generally stabilize when they bind with drug molecules. As the temperature rises, proteins tend to denature; however, when proteins are bound to drugs, the amount of undenatured protein at the same temperature increases, causing a rightward shift in the thermal melting curve of the protein‐drug complex. Specifically, after LX2 cells are treated or not treated with DATs for 3 h, the incubated cells are divided into eight aliquots. Each aliquot is then heated for 3 min at different temperatures (43, 46, 49, 52, 55, 58, 61, and 64 °C). The heated cells are subsequently stored at −80 °C for 12 h, then left at room temperature for 5 min, and this freeze–thaw cycle is repeated twice to ensure complete cell lysis. Following this, the cell lysate is centrifuged at 16 000 × *g* for 20 min, and the supernatant is collected for protein denaturation analysis. The denatured protein is then assessed by Western blot to measure RAB18 levels, and the thermal melting curve is plotted for comparison.

### Bioinformatics Analysis

In this study, the GSE84044 dataset, a gene expression dataset associated with hepatitis B virus‐related liver fibrosis, was utilized. Gene expression profiling was conducted using the Affymetrix Human Genome U133 Plus 2.0 microarray platform. Data preprocessing included background correction, normalization, and summarization using the Robust Multiarray Average algorithm. Low‐expressed and nonspecific probes were filtered, and batch effects were corrected with ComBat. Data quality was assessed through boxplot and principal component analysis to ensure sample distribution was reasonable. Postcorrection, differential gene expression analysis was performed using the limma package, with criteria set to FDR < 0.05 and |log2 Fold Change| > 1, with multiple testing corrections by Benjamini‐Hochberg. WGCNA was conducted to identify gene modules associated with fibrosis progression. GSEA was performed on differentially expressed genes with KEGG and GO gene sets from MSigDB, using FDR < 0.05 as a significance threshold. All analyses were completed in the R environment.

### Fluorescence Colocalization and Manders’ Coefficient Calculation

Manders’ coefficient was used to quantify the degree of colocalization between two fluorescent markers within cells. Samples were imaged using a confocal laser scanning microscope, ensuring minimal crosstalk between fluorescence channels. The ImageJ software JACoP plugin was used for colocalization analysis, with background correction applied to exclude nonspecific fluorescence. Two parameters, M1 and M2, were calculated, with M1 representing the overlap proportion of the first marker in relation to the second, and M2 representing the reverse. Fluorescence intensity correlations across all pixels were analyzed, with multiple fields sampled to ensure reliable data.

### SPR Affinity Assay

SPR technology was employed to assess the binding affinity and kinetic parameters between the RAB18 protein and DATs drugs. In this experiment, the RAB18 protein was immobilized on the surface of an SPR sensor chip, and DATs were injected into the flow cell at varying concentrations (6.25, 12.5, 25, 50, 100, and 200 µm). As DATs bound to the RAB18 protein, changes in the surface refractive index were observed, resulting in variations in the SPR signal. The signal changes were quantified in relative units (RU), with the *y*‐axis representing the signal change (RU) and the *x*‐axis representing time. By monitoring the binding and dissociation processes in real time, data fitting was performed using the multicycle kinetics method, yielding the association rate constant (kon), dissociation rate constant (koff), and affinity constant (Kd). This method, through multiple cycles of experiments and concentration response curves, combined with the Langmuir adsorption model or other adapted models, precisely calculated the binding affinity between DATs and the RAB18 protein. Finally, by analyzing the binding and dissociation curves, the binding strength and specificity of the drug to the target protein were comprehensively evaluated.

### Statistical Analysis

All experiments were independently conducted in triplicate, with each experiment containing at least three technical replicates. Data are expressed as mean ± standard deviation (SD), and the exact sample sizes for each analysis are detailed in the corresponding figure legends. Prior to statistical testing, the Shapiro–Wilk test was used to assess the normality of data distribution. For datasets exhibiting a normal distribution, one‐way ANOVA followed by Tukey's post hoc test was employed to evaluate differences among multiple groups. When the data did not satisfy the assumption of normality and the sample size was limited, nonparametric methods such as the Kruskal–Wallis H test were applied. All statistical analyses were performed using SPSS software (version 17.0). Statistical significance was defined as follows: ns (not significant), *p < 0.05, **p < 0.01.

## Conflict of Interest

The authors declare no conflict of interest.

## Author Contributions

H.T. was involved in the experiments and analysis of the data. S.S. was responsible for the construction of the BDL (Bile Duct Ligation) liver fibrosis mouse model and the extraction of primary HSCs. J.C. was involved in providing technical support for atomic force microscopy operation. X.Q., J.W., Y.G., X.H., Z.B., X.G., M.H., Y.S., and Y.L. participated in experiments. F.Z., Z.Z., and F.W. helped in the experimental design and methodology. J.S. and S.Z. were involved in writing and revising the article as well as organizing and analyzing the data.

## Supporting information



Supporting Information

## Data Availability

Research data are not shared.
