# Supplementary material for: Diallyl Trisulfide From Garlic Regulates RAB18 Phase Separation to Inhibit Lipophagy and Induce Cuproptosis in Hepatic Stellate Cells for Antifibrotic Effects
Source: Adv Sci (Weinh). 2025 Apr 11;12(21):2415325. doi: 10.1002/advs.202415325 (PMC12140386; doi:10.1002/advs.202415325)
Supplement: Supplementary file 1 — Supporting Information [file ADVS-12-2415325-s001.docx]

Supporting Information

Diallyl Trisulfide from Garlic Regulates RAB18 Phase Separation to Inhibit Lipophagy and Induce Cuproptosis in Hepatic Stellate Cells for Anti-Fibrotic Effects

Haoyuan Tian, Shujiang Sun, Xinran Qiu, Junrui Wang, Yuanyuan Gao, Jianmei Chen, Xiang Han, Zhengyang Bao, Xiaohan Guo, Yuqi Sun, Yuxin Lin, Mengru Hu, Zili Zhang, Feng Zhang, Feixia Wang, Shizhong Zheng*, Jiangjuan Shao*

Figure S1


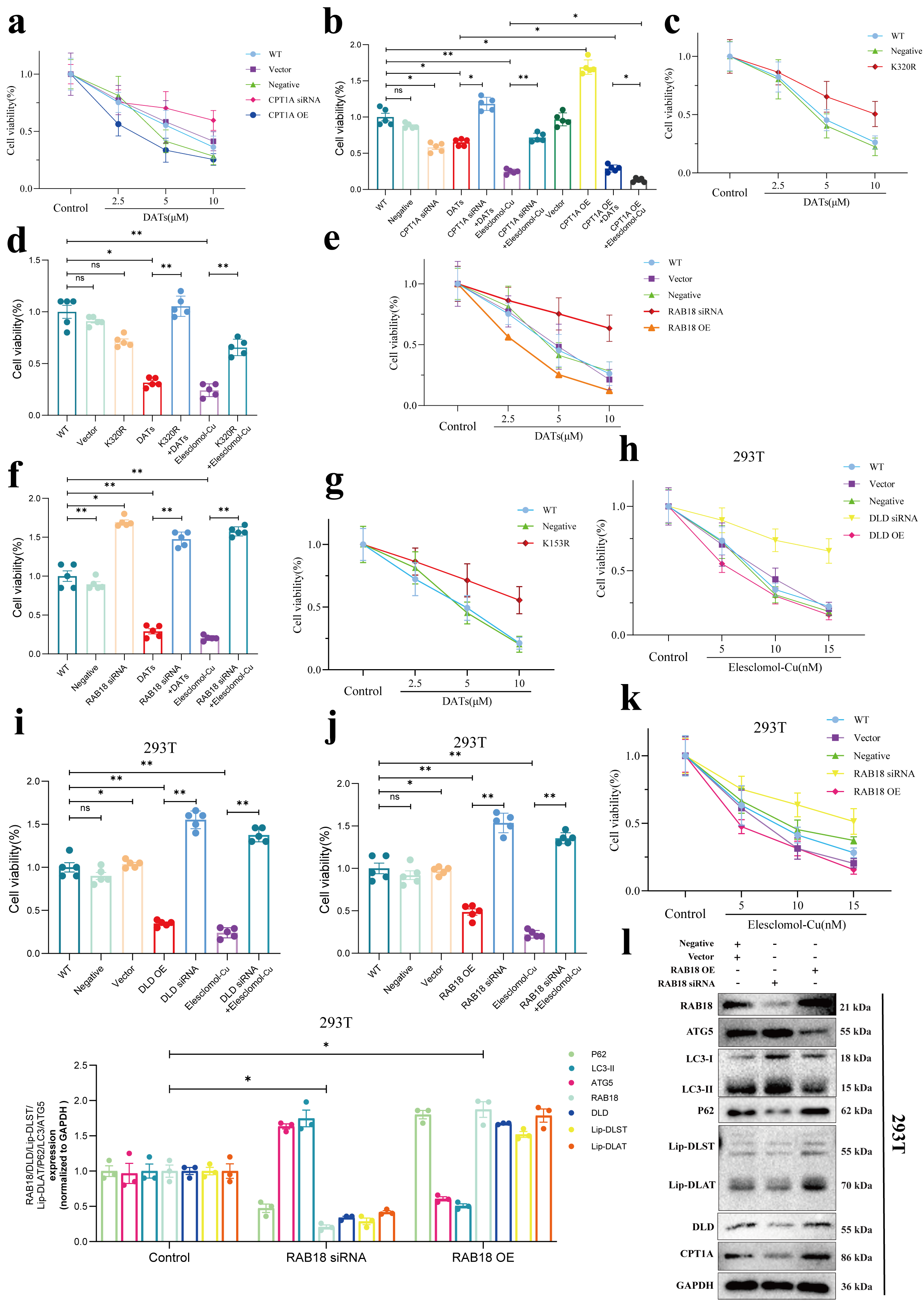


**Figure S1.** (a) LX-2 cells were treated with DATs (0-10 μM) for 24 hours. CCK8 assay was performed to evaluate the cell viability of LX-2 cells transfected with CPT1A siRNA, negative control siRNA/Vector, or CPT1A OE (n = 5). **(b)** CCK8 assay was performed to evaluate the cell viability of LX-2 cells transfected with CPT1A siRNA or negative control siRNA, and treated with DATs (10 μM) or Elesclomol-Cu (10 nM) (n = 5).  **(c)** LX-2 cells were treated with DATs (0-10 μM) for 24 hours. CCK8 assay was performed to evaluate the cell viability of LX-2 cells transfected with DLD-K320R or negative control siRNA (n = 5). **(d)** CCK8 assay was performed to evaluate the cell viability of LX-2 cells transfected with DLD-K320R or negative control siRNA, and treated with DATs (10 μM) or Elesclomol-Cu (10 nM) (n = 5). **(e)** LX-2 cells were treated with DATs (0-10 μM) for 24 hours. CCK8 assay was performed to evaluate the cell viability of LX-2 cells transfected with RAB18 siRNA, negative control siRNA/Vector, or RAB18 OE (n = 5). **(f)** CCK8 assay was performed to evaluate the cell viability of LX-2 cells transfected with RAB18 siRNA, negative control siRNA/Vector, and treated with DATs (10 μM) or Elesclomol-Cu (10 nM) (n = 5).  **(g)** LX-2 cells were treated with DATs (0-10 μM) for 24 hours. CCK8 assay was performed to evaluate the cell viability of LX-2 cells transfected with RAB18-K153R or negative control siRNA (n = 5). **(h)** 293T cells were treated with Elesclomol-Cu (0-10 μM) for 24 hours. CCK8 assay was performed to evaluate the cell viability of 293T cells transfected with DLD siRNA, negative control siRNA/Vector, or DLD OE (n = 5).  **(i)** CCK8 assay was performed to evaluate the cell viability of 293T cells transfected with DLD siRNA, negative control siRNA/Vector, or DLD OE, and treated with Elesclomol-Cu (10 nM) (n = 5). **(j)** 293T cells were treated with Elesclomol-Cu (0-10 μM) for 24 hours. CCK8 assay was performed to evaluate the cell viability of 293T cells transfected with RAB18 siRNA, negative control siRNA/Vector, or RAB18 OE (n = 5). **(k)** CCK8 assay was performed to evaluate the cell viability of 293T cells transfected with RAB18 siRNA, negative control siRNA/Vector, or RAB18 OE, and treated with Elesclomol-Cu (10 nM) (n = 5).  **(l)** Immunoblotting was performed to assess the expression levels of RAB18, ATG5, LC3, DLD, CPT1A, and lipoic acid-modified proteins in 293T cells under conditions of RAB18 siRNA or OE RAB18, followed by grayscale analysis for quantification (n = 3). Data are presented as mean ± SD, with p-values calculated using one-way ANOVA. ns, not significant; *p < 0.05, **p < 0.01.

Figure S2


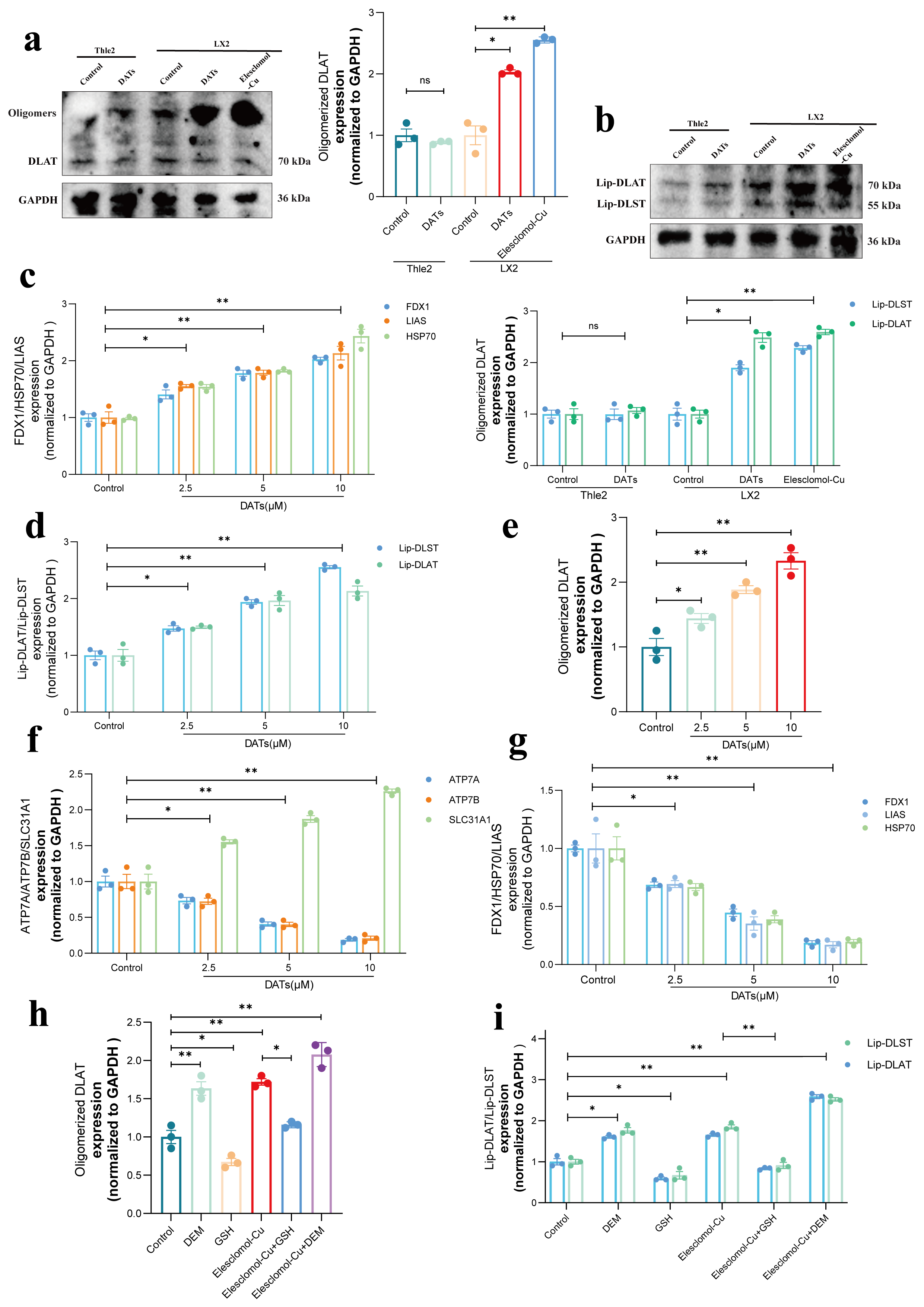


**Figure S2. (a)** LX-2 cells were treated with DATs (10 μM) for 24 hours. The expression levels of oligomerized DLAT protein were evaluated and quantified using grayscale analysis in Thle-2 cells treated with DATs (10 μM), or LX-2 cells treated with DATs (10 μM) or Elesclomol-Cu (10 nM) for 24 hours (n = 3). **(b)** LX-2 cells were treated with DATs (10 μM) for 24 hours. The expression levels of lipoic acid-modified proteins were evaluated and quantified using grayscale analysis in Thle-2 cells treated with DATs (10 μM), or LX-2 cells treated with DATs (10 μM) or Elesclomol-Cu (10 nM) for 24 hours (n = 3). **(c)** The expression levels of FDX1, LIAS, and HSP70 proteins were quantified using grayscale analysis in LX-2 cells treated with DATs (0-10 μM) for 24 hours (n = 3).  **(d)** The expression levels of lipoic acid-modified proteins were quantified using grayscale analysis in LX-2 cells treated with DATs (0-10 μM) for 24 hours (n = 3).  **(e)** The expression levels of oligomerized DLAT protein were quantified using grayscale analysis in LX-2 cells treated with DATs (0-10 μM) for 24 hours (n = 3). **(f)** The expression levels of SLC31A1, ATP7A, and ATP7B proteins were quantified using grayscale analysis in LX-2 cells treated with DATs (0-10 μM) for 24 hours (n = 3).  **(g)** The expression levels of FDX1, LIAS, and HSP70 proteins were quantified using grayscale analysis in LX-2 cells treated with DATs (0-10 μM) for 48 hours (n = 3). **(h)** Grayscale analysis was performed to quantify the expression levels of lipoic acid-modified proteins in LX-2 cells treated with DEM (200 μM) or GSH (1 mM) for 24 hours, in the presence or absence of Elesclomol-Cu (10 nM) (n = 3). **(i)** Grayscale analysis was performed to quantify the expression levels of oligomerized DLAT protein in LX-2 cells treated with DEM (200 μM) or GSH (1 mM) for 24 hours, in the presence or absence of Elesclomol-Cu (10 nM) (n = 3). Data are presented as mean ± SD, with p-values calculated using one-way ANOVA. ns, not significant; *p < 0.05, **p < 0.01.

Figure S3

**
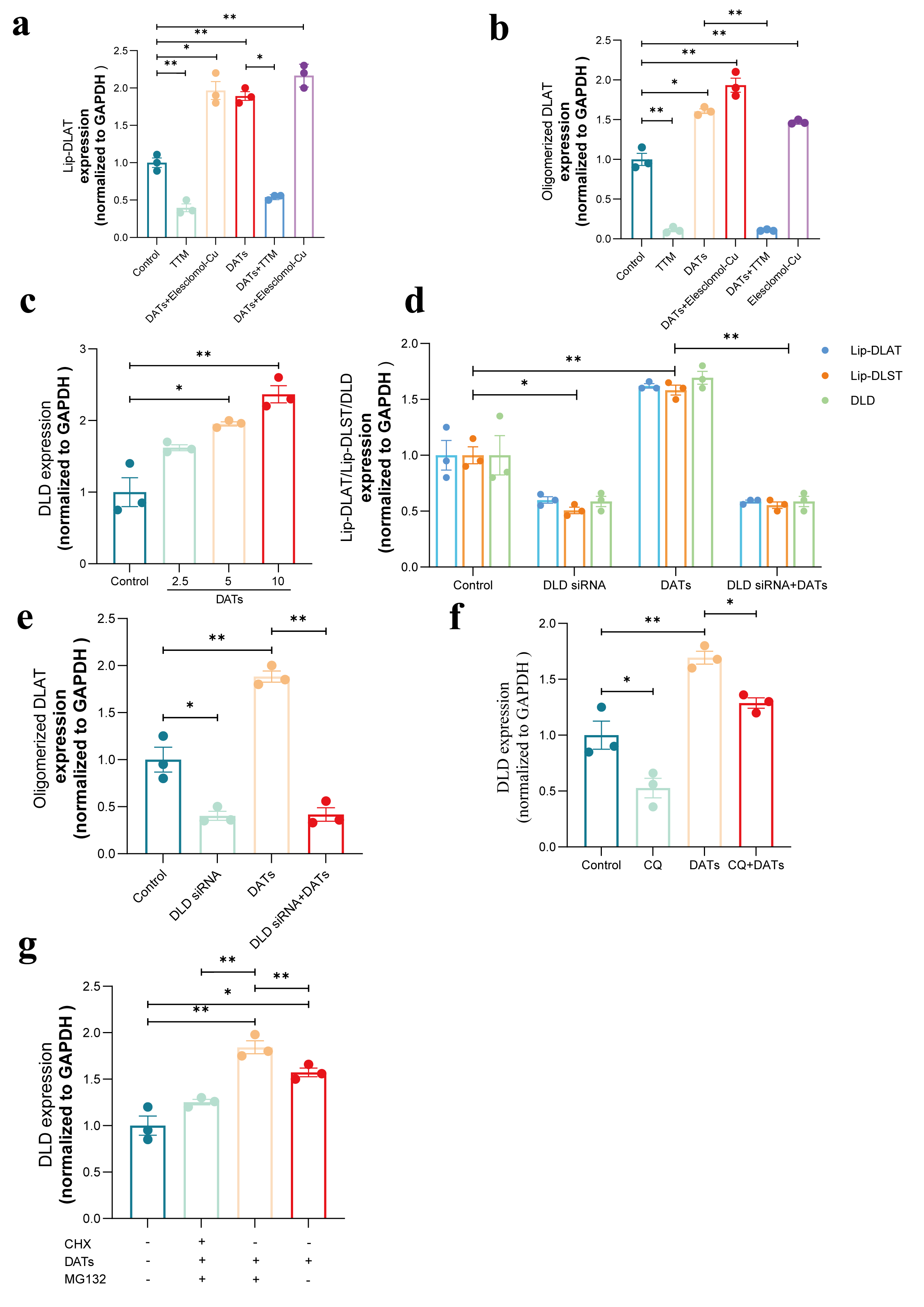
Figure S3. (a)** LX-2 cells were treated with Elesclomol-Cu (10 nM) or TTM (5 μM) for 24 hours, in the presence or absence of DATs (10 μM). The expression levels of oligomerized DLAT protein were quantified using grayscale analysis (n = 3).  **(b)** LX-2 cells were treated with Elesclomol-Cu (10 nM) or TTM (5 μM) for 24 hours, in the presence or absence of DATs (10 μM). The expression levels of lipoic acid-modified DLAT protein were quantified using grayscale analysis (n = 3).  **(c)** LX-2 cells were treated with DATs (0-10 μM) for 24 hours. The expression levels of DLD protein were quantified using grayscale analysis (n = 3). **(d)** LX-2 cells transfected with DLD siRNA or negative control siRNA were treated with DATs (10 μM) for 24 hours. The expression levels of lipoic acid-modified proteins were quantified using grayscale analysis (n = 3).  **(e)** LX-2 cells transfected with DLD siRNA or negative control siRNA were treated with DATs (10 μM) for 24 hours. The expression levels of oligomerized DLAT protein were quantified using grayscale analysis (n = 3).  **(f)** LX-2 cells were treated with CQ (5 μM) for 24 hours, in the presence or absence of DATs (10 μM). The expression levels of DLD protein were quantified using grayscale analysis (n = 3).  **(g)** LX-2 cells were treated with CHX (20 μg/ml), MG132 (10 μM) alone or in combination for 24 hours, in the presence or absence of DATs (10 μM). The expression levels of DLD protein were quantified using grayscale analysis (n = 3). Data are presented as mean ± SD, with p-values calculated using one-way ANOVA. ns, not significant; *p < 0.05, **p < 0.01.

Figure S4


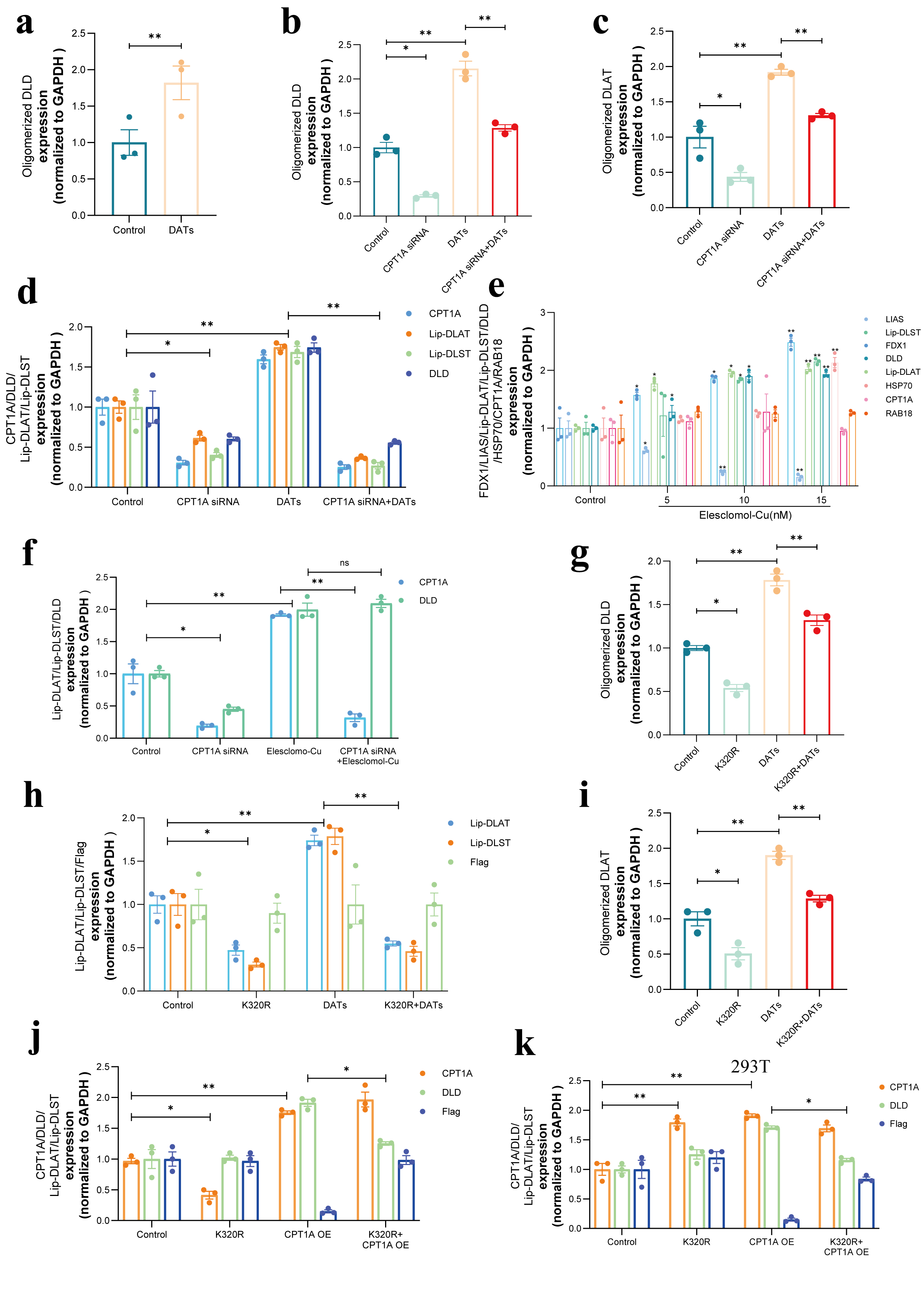


**Figure S4. (a)** Grayscale analysis quantification of oligomerized DLD protein expression levels in LX-2 cells treated with DATs (10 μM) (n = 3).  **(b)** Grayscale analysis quantification of oligomerized DLD protein expression levels in LX-2 cells treated with DATs (10 μM) and transfected with CPT1A siRNA or negative control siRNA (n = 3). **(c)** Grayscale analysis quantification of oligomerized DLAT protein expression levels in LX-2 cells treated with DATs (10 μM) and transfected with CPT1A siRNA or negative control siRNA (n = 3).  **(d)** Grayscale analysis quantification of CPT1A, DLD, and lipoic acid-modified protein expression levels in LX-2 cells treated with DATs (10 μM) and transfected with CPT1A siRNA or negative control siRNA (n = 3).  **(e)** Grayscale analysis quantification of CPT1A, DLD, FDX1, LIAS, HSP70, and other protein expression levels in LX-2 cells treated with Elesclomol-Cu (0-10 nM) for 24 hours (n = 3). **(f)** Grayscale analysis quantification of DLD protein and lipoic acid-modified protein expression levels in LX-2 cells treated with Elesclomol-Cu (10 nM) for 24 hours and transfected with CPT1A siRNA or negative control siRNA (n = 3).  **(g)** Grayscale analysis quantification of oligomerized DLD protein expression levels in LX-2 cells treated with DATs (10 μM) and transfected with FLAG-tagged DLD-320R (n = 3). **(h)** Grayscale analysis quantification of lipoic acid-modified protein expression levels in LX-2 cells treated with DATs (10 μM) and transfected with FLAG-tagged DLD-320R (n = 3). **(i)** Grayscale analysis quantification of oligomerized DLAT protein expression levels in LX-2 cells treated with DATs (10 μM) and transfected with FLAG-tagged DLD-320R (n = 3).  **(j)** Grayscale analysis quantification of oligomerized CPT1A and DLD protein expression levels in LX-2 cells treated with CPT1A OE and transfected with DLD-320R (n = 3). **(k)** Grayscale analysis quantification of oligomerized CPT1A and DLD protein expression levels in 293T cells treated with CPT1A OE and transfected with DLD-320R (n = 3). Data are presented as mean ± SD, with p-values calculated using one-way ANOVA. ns, not significant; *P < 0.05, **P < 0.01.

Figure S5


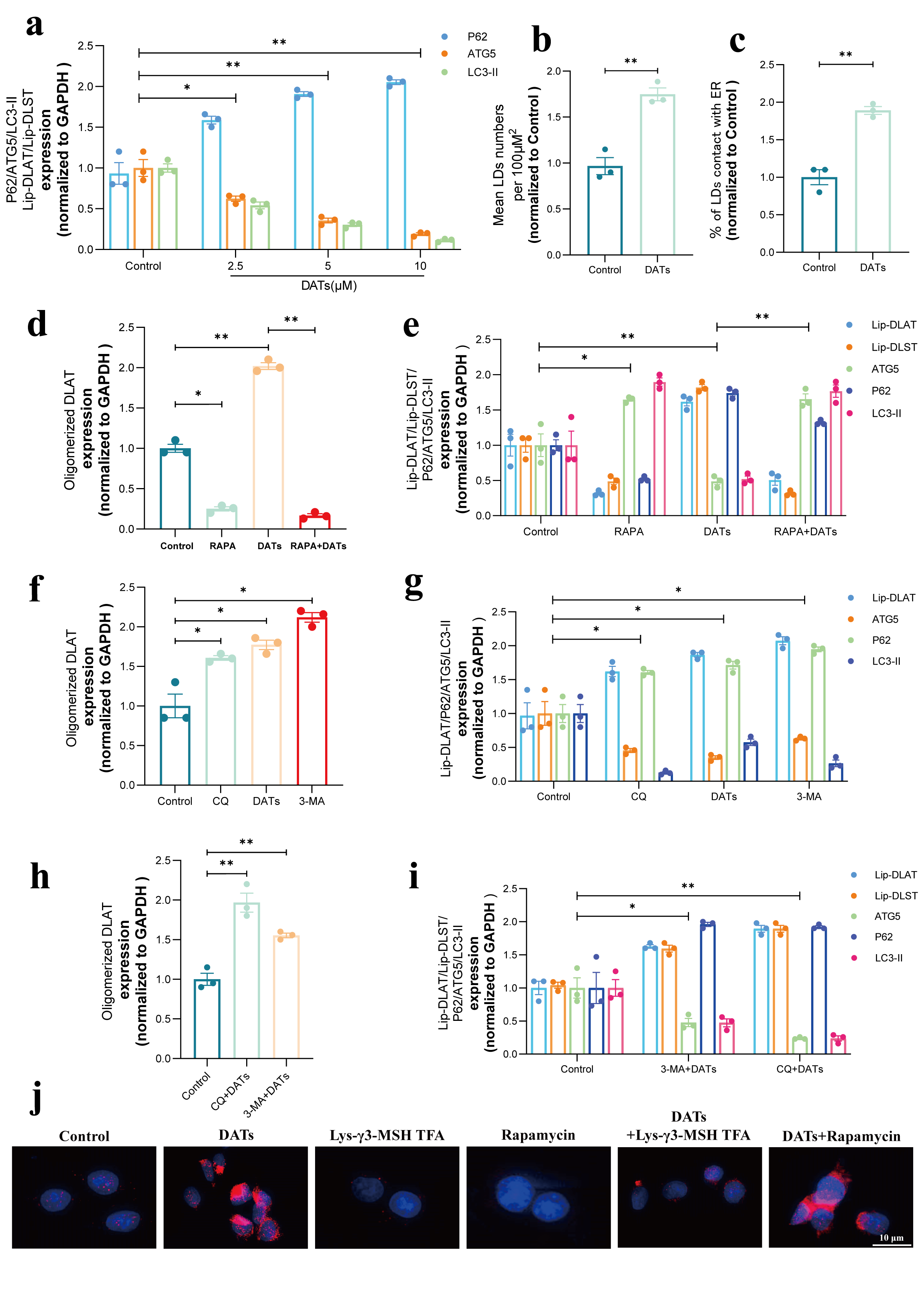


**Figure S5. (a)** Grayscale analysis quantification of ATG5, LC3-II, and p62 expression levels in LX-2 cells treated with DATs (0-10 μM) for 24 hours (n = 3). **(b)** Quantification of lipid droplet numbers per 100 μm² in transmission electron microscope images (n = 3).  **(c)** Quantification of lipid droplet numbers on the endoplasmic reticulum in transmission electron microscope images (n = 3). **(d)** Grayscale analysis quantification of lipoic acid-modified proteins, P62, ATG5, and LC-3 expression levels in LX-2 cells treated with DATs (10 μM) for 24 hours and autophagy activator intervention (n = 3). **(e)** Grayscale analysis quantification of oligomerized DLAT protein expression levels in LX-2 cells treated with DATs (10 μM) for 24 hours and autophagy activator RAPA intervention (n = 3). **(f, g)** Grayscale analysis quantification of lipoic acid-modified proteins, P62, ATG5, and LC-3 expression levels in LX-2 cells treated with DATs (10 μM) for 24 hours, in the presence or absence of CQ (5 μM) or 3-MA (10 μM) (n = 3).  **(h, i)** Grayscale analysis quantification of oligomerized DLAT protein expression levels in LX-2 cells treated with DATs (10 μM) for 24 hours, in the presence or absence of CQ (5 μM) or 3-MA (10 μM) (n = 3).  **(j)** Fluorescence images of lipid droplet numbers in LX-2 cells treated with Lys-γ3-MSH TFA (1 μM) or Rapamycin (200 nM) for 24 hours, in the presence or absence of DATs (10 μM) (n = 3). Scale bar: 10 μm. Data are presented as mean ± SD, with p-values calculated using one-way ANOVA. ns, not significant; *P < 0.05, **P < 0.01.

Figure S6


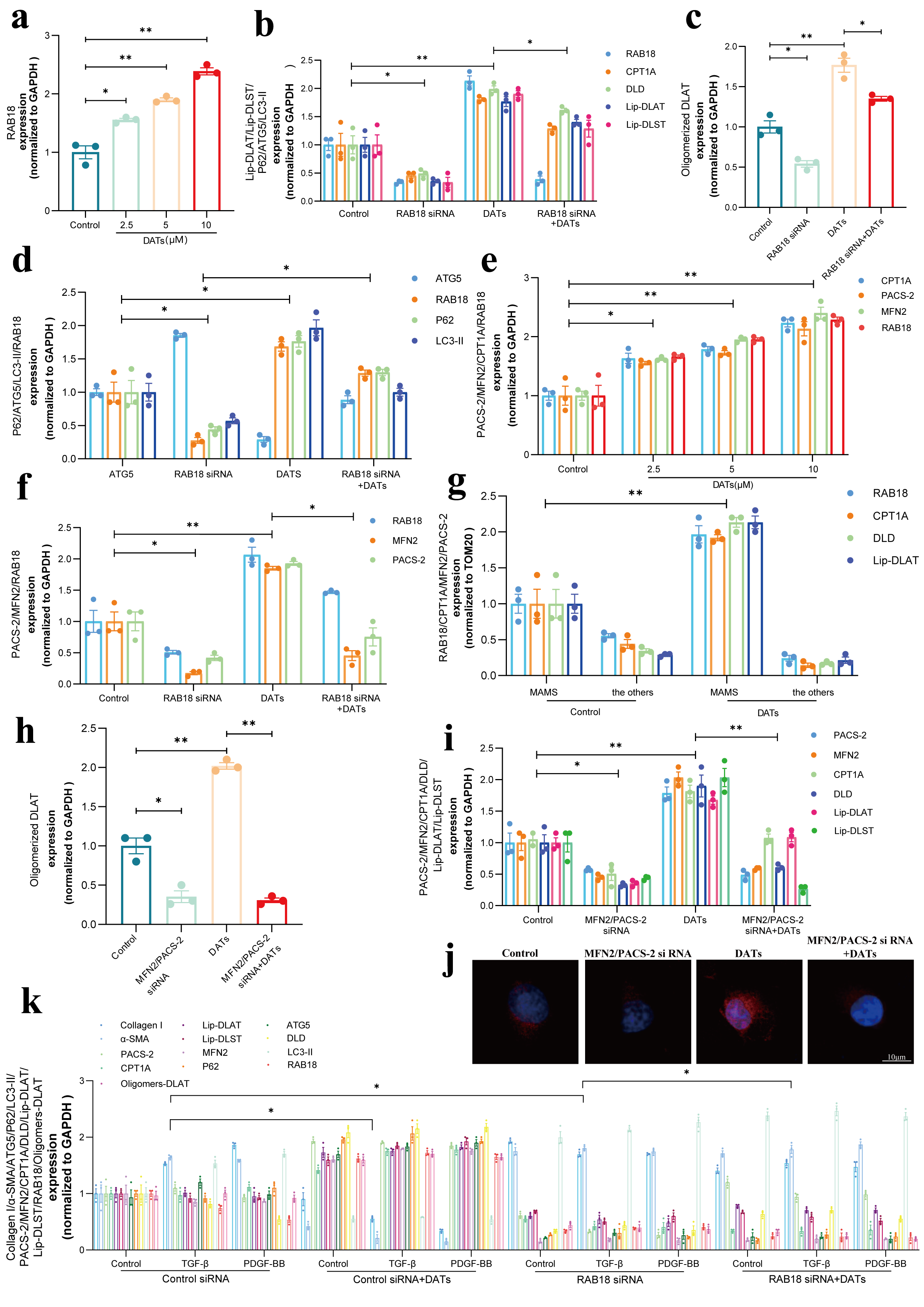


**Figure S6. (a)** Grayscale analysis quantification of RAB18 protein expression levels in LX-2 cells treated with DATs (0-10 μM) for 24 hours (n = 3). **(b)** Grayscale analysis quantification of CPT1A, DLD, and lipoic acid-modified protein expression levels in LX-2 cells treated with DATs (10 μM) and transfected with RAB18 siRNA or negative control siRNA (n = 3).  **(c)** Grayscale analysis quantification of oligomerized DLAT protein expression levels in LX-2 cells treated with DATs (10 μM) and transfected with RAB18 siRNA or negative control siRNA (n = 3). **(d)** Grayscale analysis quantification of ATG5, P62, and LC-3 protein expression levels in LX-2 cells treated with DATs (10 μM) and transfected with RAB18 siRNA or negative control siRNA (n = 3). **(e)** Grayscale analysis quantification of CPT1A, PACS-2, MFN2, and RAB18 protein expression levels in LX-2 cells treated with DATs (0-10 μM) for 24 hours (n = 3). **(f)** Grayscale analysis quantification of PACS-2 and MFN2 protein expression levels in LX-2 cells treated with DATs (10 μM) for 24 hours and transfected with RAB18 siRNA or negative control siRNA (n = 3). **(g)** Grayscale analysis quantification of CPT1A, PACS-2, MFN2, and RAB18 protein expression levels in MAMs structures isolated by gradient centrifugation from LX-2 cells treated with DATs (10 μM) for 24 hours (n = 3).  **(h)** Grayscale analysis quantification of oligomerized DLAT protein expression levels in LX-2 cells treated with DATs (10 μM) for 24 hours and transfected with MFN2/PACS-2 siRNA or negative control siRNA (n = 3).  **(i)** Grayscale analysis quantification of CPT1A, PACS-2, MFN2, DLD, and lipoic acid-modified protein expression levels in LX-2 cells treated with DATs (10 μM) for 24 hours and transfected with MFN2/PACS-2 siRNA or negative control siRNA (n = 3).  **(j)** Immunofluorescence images of lipid droplet numbers in LX-2 cells treated with DATs (10 μM) for 24 hours and transfected with MFN2/PACS-2 siRNA or negative control siRNA (n = 3). Scale bar: 10 μm.  **(k)** Grayscale analysis quantification of RAB18, ATG5, P62, LC-3, CPT1A, PACS-2, MFN2, DLD, α-SMA, Collagen I, lipoic acid-modified proteins, and oligomerized DLAT protein expression levels in LX-2 cells activated with TGF-β/PDGF-BB and treated with DATs (10 μM) for 24 hours (n = 3). Data are presented as mean ± SD, with p-values calculated using one-way ANOVA. ns, not significant; *P < 0.05, **P < 0.01.

Figure S7


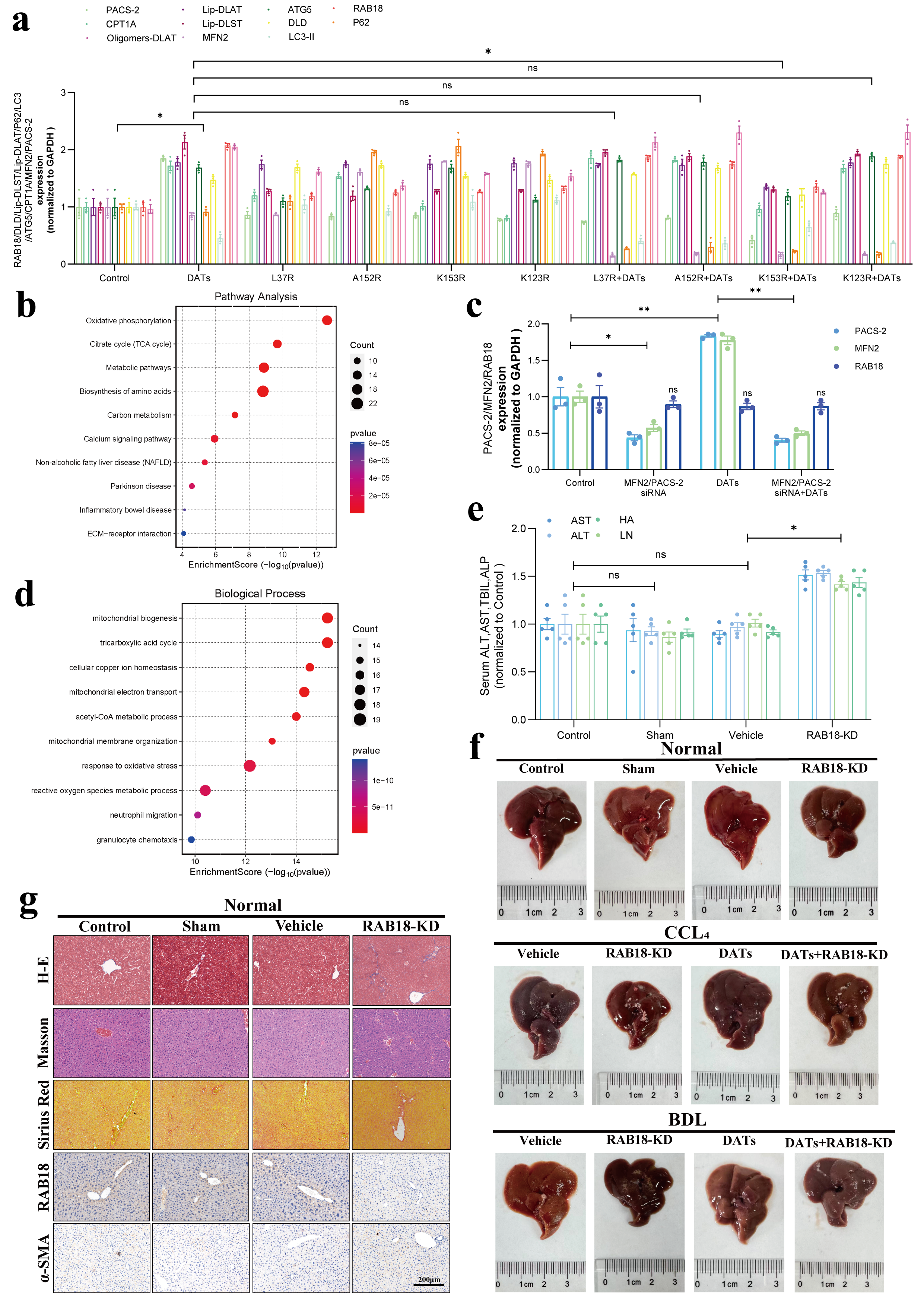


**Figure S7**. **(a)** Immunoblotting was performed to assess the expression levels of RAB18, ATG5, P62, LC-3, CPT1A, PACS-2, MFN2, DLD, lipoic acid-modified proteins, and oligomerized DLAT proteins in RAB18 amino acid point-mutated LX-2 cells treated with DATs (10 μM) for 24 hours, with quantification using grayscale analysis (n = 3).  **(b)** Proteomic analysis was conducted to identify differentially expressed proteins in LX-2 cells overexpressing RAB18, followed by KEGG pathway enrichment analysis (n = 3). **(c)** Grayscale analysis quantification of MFN2, PACS-2, and RAB18 protein expression levels in LX-2 cells treated with DATs (10 μM) and transfected with MFN2/PACS-2 siRNA or negative control siRNA (n = 3). **(d)** Proteomic analysis was performed to identify differentially expressed proteins in LX-2 cells overexpressing RAB18, followed by Biological Process enrichment analysis of mitochondrial-related processes (n = 3).  **(e)** Serum analysis was performed to evaluate the expression levels of ALT, AST, TBIL, and ALP in serum collected from normal mice (control group, Sham group, Vehicle group, and RAB18-KD group, n = 5).  **(f)** Liver photos were collected from normal mice (control group, Sham group, Vehicle group, and RAB18-KD group) and CCl4/BDL-induced mouse liver fibrosis models (Vehicle group, RAB18-KD group, DATs-treated group, and RAB18-KD + DATs-treated group, n = 5).  **(g)** H&E staining, Masson, Sirius Red, and α-SMA, RAB18 IHC analysis were performed on liver tissue collected from normal mice (control group, Sham group, Vehicle group, and RAB18-KD group) (n = 5). Scale bar: 200 μm. Data are presented as mean ± SD, with p-values calculated using one-way ANOVA. ns, not significant; *P < 0.05, **P < 0.01.

Figure S8


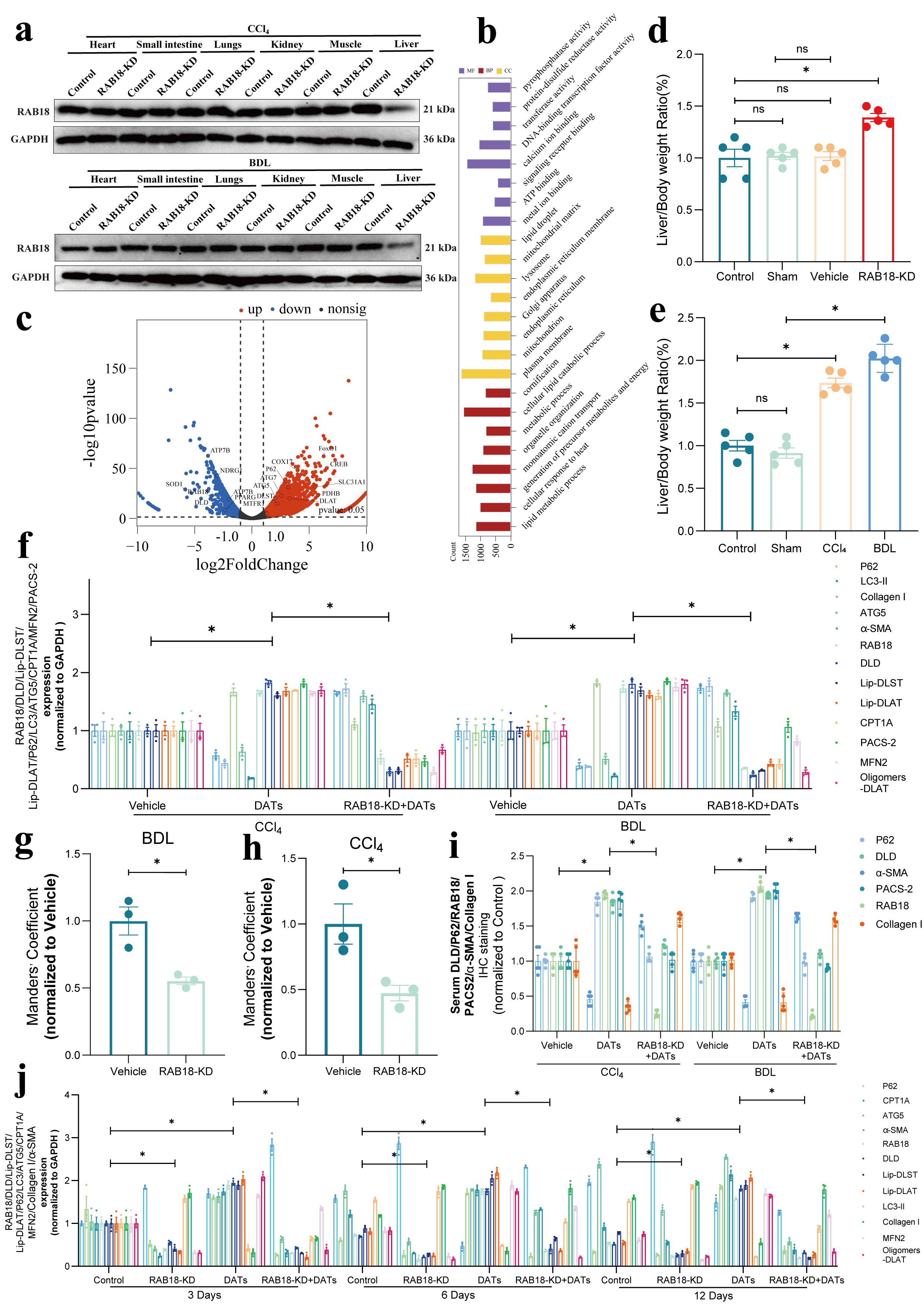


**Figure S8.** **(a)** Grayscale analysis quantification of RAB18 protein expression levels in heart, liver, lung, kidney, muscle, and small intestine from CCl4/BDL-induced mouse liver fibrosis models (Vehicle and RAB18-KD groups, n = 5) (n = 3). **(b, c)** Transcriptomic analysis was performed to identify gene expression differences between normal mouse liver tissue and fibrotic liver tissue, with volcano plot and KEGG pathway analysis (n = 3).  **(d)** Liver weight-to-body weight ratio from normal mice (control group, Sham group, Vehicle group, and RAB18-KD group) (n = 5). **(e)** Liver weight-to-body weight ratio from normal mice (control group and Sham group) and CCl4/BDL-induced mouse liver fibrosis models (Vehicle group and RAB18-KD group) (n = 5). **(f)** Grayscale analysis quantification of α-SMA, Collagen I, RAB18, ATG5, P62, LC-3, CPT1A, PACS-2, MFN2, DLD, lipoic acid-modified proteins, and oligomerized DLAT protein expression levels in liver tissue collected from CCl4/BDL-induced mouse liver fibrosis models (Vehicle group, DATs-treated group, and RAB18-KD + DATs-treated group, n = 5) (n = 3). **(g)** Co-localization analysis of LDs (red) and α-SMA (green) in liver tissue from CCl4-induced mouse liver fibrosis models treated with Vehicle or DATs (n = 3) **(h)** Co-localization analysis of LDs (red) and α-SMA (green) in liver tissue from BDL-induced mouse liver fibrosis models treated with Vehicle or DATs (n = 3). **(i)** α-SMA, Collagen I, RAB18, DLD, PACS-2, and P62 IHC analysis were performed on liver tissue collected from CCl4/BDL-induced mouse liver fibrosis models treated with Vehicle, DATs, or RAB18-KD + DATs (n = 5). **(j)** Grayscale analysis quantification of α-SMA, Collagen I, RAB18, ATG5, P62, LC-3, CPT1A, PACS-2, MFN2, DLD, lipoic acid-modified proteins, and oligomerized DLAT protein expression levels in primary HSCs isolated from control and RAB18-KD mouse livers and treated with DATs (10 μM) (n = 3). Data are presented as mean ± SD, with p-values calculated using one-way ANOVA. ns, not significant; *P < 0.05, **P < 0.01.

Figure S9


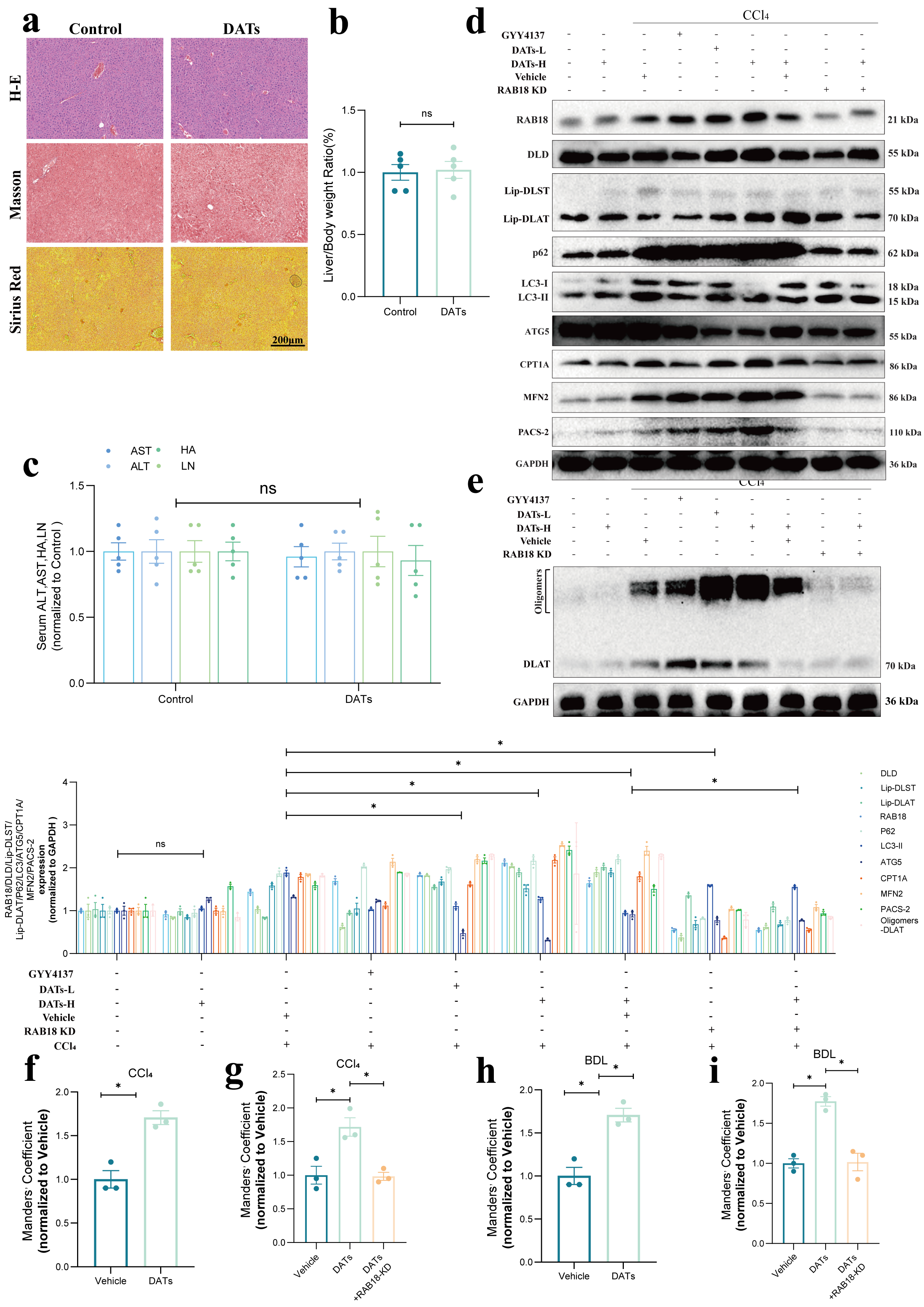


**Figure S9.** **(a)** H&E staining, Masson, and Sirius Red analysis of liver tissue collected from normal mice in the control group and DATs-treated group (n = 5). Scale bar: 200 μm. **(b)** Liver weight-to-body weight ratio from normal mice in the control group and DATs-treated group (n = 5).  **(c)** Serum analysis was conducted to assess the expression levels of ALT, AST, TBIL, and ALP in serum collected from normal mice in the control group and DATs-treated group (n = 5). **(d, e)** Liver tissue collected from CCl4-induced mouse liver fibrosis models treated with low-dose DATs (15 mg/kg), high-dose DATs (15 mg/kg), and GYY4137 as a positive drug, combined with AAV8-RAB18KD pre-treatment. Grayscale analysis quantification of α-SMA, Collagen I, RAB18, ATG5, P62, LC-3, CPT1A, PACS-2, MFN2, DLD, lipoic acid-modified proteins, and oligomerized DLAT protein expression levels (n = 3). **(f)** Co-localization analysis of α-SMA (red) and Copper (green) in liver tissue from CCl4-induced mouse liver fibrosis models treated with Vehicle or DATs (n = 3). **(g)** Co-localization analysis of α-SMA (green) and LDs (red) in liver tissue from CCl4-induced mouse liver fibrosis models treated with Vehicle, DATs, or RAB18-KD + DATs (n = 3). **(h)** Co-localization analysis of α-SMA (red) and Copper (green) in liver tissue from BDL-induced mouse liver fibrosis models treated with Vehicle or DATs (n = 3).  **(i)** Co-localization analysis of α-SMA (green) and LDs (red) in liver tissue from BDL-induced mouse liver fibrosis models treated with Vehicle, DATs, or RAB18-KD + DATs (n = 3). Data are presented as mean ± SD, with p-values calculated using one-way ANOVA. ns, not significant; *P < 0.05, **P < 0.01.

Figure S10


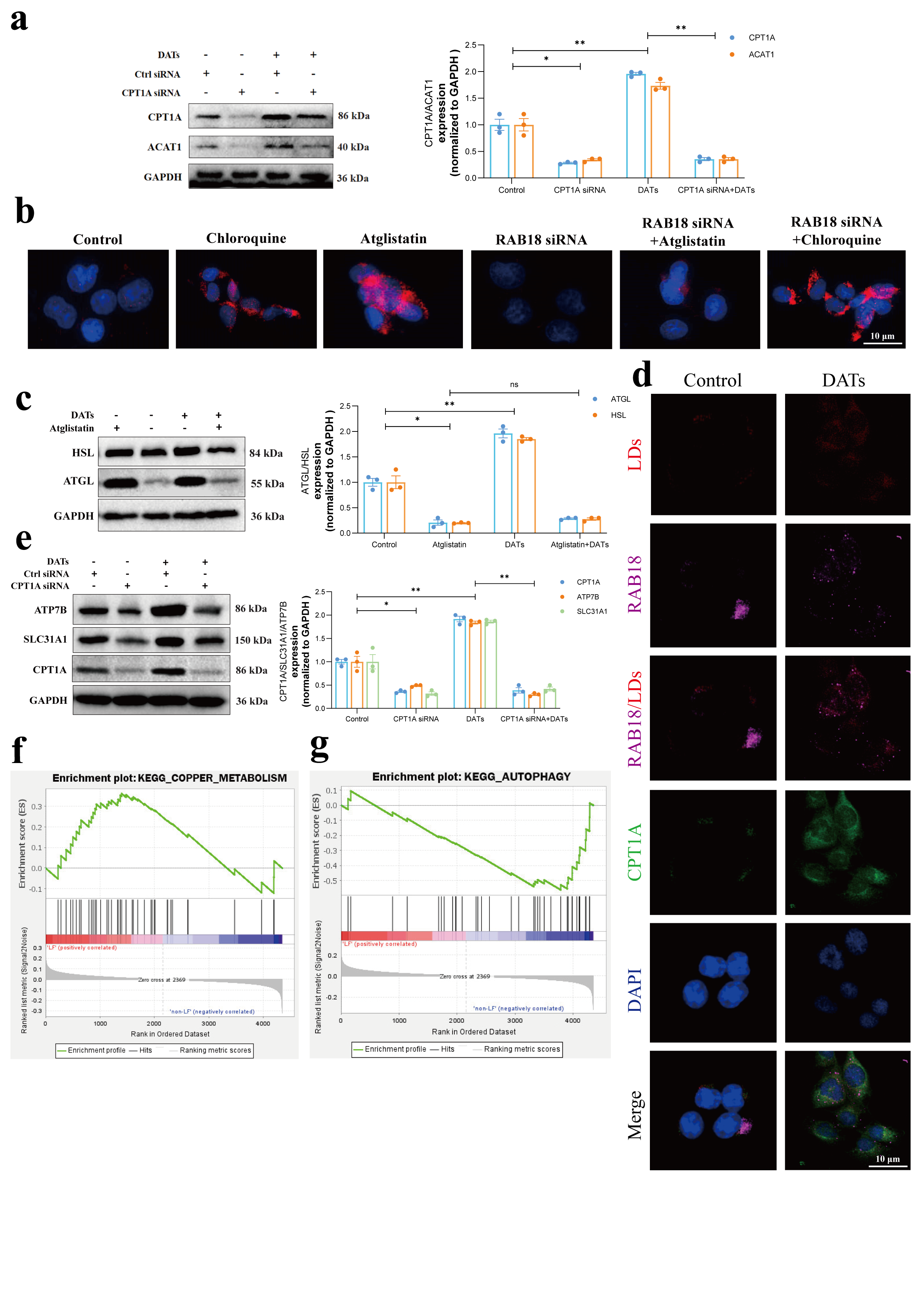


**Figure S10.** **(a)** Immunoblotting was performed to assess the expression levels of CPT1A and ACAT1 proteins in LX-2 cells treated with DATs (10 μM) for 24 hours and transfected with CPT1A siRNA or negative control siRNA, with quantification using grayscale analysis (n = 3). **(b)** Fluorescence images of lipid droplet numbers in LX-2 cells treated with Chloroquine (5 μM) or Atglistatin (5 μM) for 24 hours, and transfected with RAB18 siRNA or negative control siRNA (n = 3). Scale bar: 10 μm.  **(c)** Immunoblotting was performed to assess the lipid droplet numbers in LX-2 cells treated with Atglistatin and DATs (10 μM) for 24 hours, with fluorescence images taken (n = 3).  **(d)** Fluorescence images of RAB18/LDs/CPT1A multi-labeling in LX-2 cells treated with DATs (10 μM) for 24 hours (n = 3). Scale bar: 10 μm. **(e)** Immunoblotting was conducted to assess the expression levels of CPT1A, ATP7B, and SLC31A1 proteins in LX-2 cells treated with DATs (10 μM) for 24 hours and transfected with CPT1A siRNA or negative control siRNA, with quantification using grayscale analysis (n = 3). **(f, g)** Protein expression analysis through proteomics and GSEA analysis was performed on LX-2 cells treated with DATs (0-10 μM) for 24 hours, revealing protein enrichment related to copper metabolism and autophagy (n = 3). Data are presented as mean ± SD, with p-values calculated using one-way ANOVA. ns, not significant; *P < 0.05, **P < 0.01.
